# FGFR2 fusion-driven cholangiocarcinoma is characterized by a distinct neutrophil-enriched tumor microenvironment in a syngeneic murine model

**DOI:** 10.1016/j.jhepr.2026.101964

**Published:** 2026-07-21

**Authors:** Nugzar Lekiashvili, Trinh Kieu Dinh, Alexander Olkus, Yu-Le Wu, Chun-Shan Liu, Daria Komkova, Albrecht Stenzinger, Felix J. Hartmann, Thomas Longerich, Michael T. Dill

**Affiliations:** 1 German Cancer Research Center (DKFZ) Heidelberg, Experimental Hepatology, Inflammation and Cancer, Heidelberg, Germany; 2 Medical Faculty Heidelberg, Heidelberg University, Heidelberg, Germany; 3 Faculty of Biosciences, Heidelberg University, Heidelberg, Germany; 4 Department of Medicine IV, Gastroenterology, Hepatology and Infectious Diseases, Heidelberg University, Heidelberg, Germany; 5 Liver Cancer Center Heidelberg (LCCH), Heidelberg, Germany; 6 German Cancer Research Center (DKFZ) Heidelberg, Systems Immunology and Single-Cell Biology, Heidelberg, Germany; 7 German Cancer Research Center (DKFZ) Heidelberg, Computational and Molecular Prevention, Heidelberg, Germany; 8 Institute of Pathology, Heidelberg University Hospital, Heidelberg, Germany; 9 German Cancer Consortium (DKTK), DKFZ Core Center, Heidelberg, Germany

**Keywords:** Liver cancer, Tumor microenvironment, Immune escape, FGFR2 fusion, Kras mutation, Organoids, Cytotoxic T cells, Neutrophil granulocytes

## Abstract

**Background & Aims:**

Fibroblast growth factor receptor 2 gene (*FGFR2*) rearrangements are among the most common oncogenic drivers in intrahepatic cholangiocarcinoma (iCCA). Although FGFR inhibitors are clinically approved, primary and secondary resistance remain major limitations. Preclinical investigation of resistance mechanisms, including cancer cell-extrinsic crosstalk, is hampered by current models that rely on human *FGFR2* fusion transgenes in immunodeficient hosts. We therefore aimed at generating an entirely murine FGFR2 fusion-driven iCCA (Ff-iCCA) model to study immunomodulatory mechanisms in the tumor microenvironment (TME).

**Methods:**

A syngeneic cholangiocyte organoid-based iCCA mouse model was engineered via endogenous chromosomal rearrangement of the *Fgfr2* gene combined with *Trp53* deletion (PFf) and other co-occurring genetic alterations. *Kras*^*G12D*^-mutated lines (PK) served as comparison. TME characterization was performed using 30-plex spatial proteomics on ∼250,000 cells. Bulk RNA sequencing was conducted on FGFR inhibitor-treated PFf and PK organoids. Pharmacodynamics of FGFR inhibition on Ff-iCCA were assessed by immunostaining and quantitative RT-PCR.

**Results:**

Intrahepatic implantation produced well-differentiated Ff-iCCA with morphologic features resembling human small duct type iCCA. In immunocompetent hosts, additional co-alterations were required for tumor penetrance, with *Pten* deletion being most robust with 75%. Compared with KRAS-driven iCCA, Ff-iCCA showed a significantly increased infiltration by Ly-6C/G+ neutrophils (19-fold, *p* = 0.004) and CD8+ T cells (eightfold, *p* <0.001). Transcriptome analysis revealed increased chemokine expression in PFf *vs*. PK organoids, which was not reversed by FGFR inhibition. Ff-iCCA responded to FGFR inhibition with a 6.5-fold reduced proliferation *in vivo* (*p* = 0.029), without observation of significant TME remodeling.

**Conclusions:**

Our syngeneic murine Ff-iCCA model recapitulates hallmarks of human Ff-iCCA, providing a platform for functional investigation of cancer cell–TME crosstalk in this molecular subtype.

**Impact and implications:**

This study introduces the first fully syngeneic, endogenously engineered murine model of FGFR2 fusion-driven iCCA, overcoming the key limitation of prior models relying on human transgenes in immunodeficient hosts. The model faithfully recapitulates hallmarks of human FGFR2 fusion-driven iCCA, including a small duct type morphology and a distinct neutrophil-enriched tumor immune microenvironment, validating its translational relevance. The finding that an upregulated chemotaxis signature in FGFR2 fusion persists despite FGFR inhibition, alongside upregulation of interferon-stimulated genes upon treatment, points to compensatory immunomodulatory mechanisms that remain mechanistically unresolved. Overall, this work provides a physiologically relevant platform to interrogate cancer cell–tumor microenvironment crosstalk in FGFR2 fusion-driven iCCA.

## Highlights

• First syngeneic murine iCCA model based on endogenous *Fgfr2* chromosomal rearrangement.
• Recapitulates small duct type morphology of human FGFR2 fusion-driven iCCA.
• FGFR2 fusion-driven iCCA shows distinct neutrophil and CD8+ T-cell enrichment.
• Chemotaxis gene upregulation in FGFR2 fusion-driven iCCA is not reversed by FGFR inhibition.

## Graphical abstract

**Figure:**
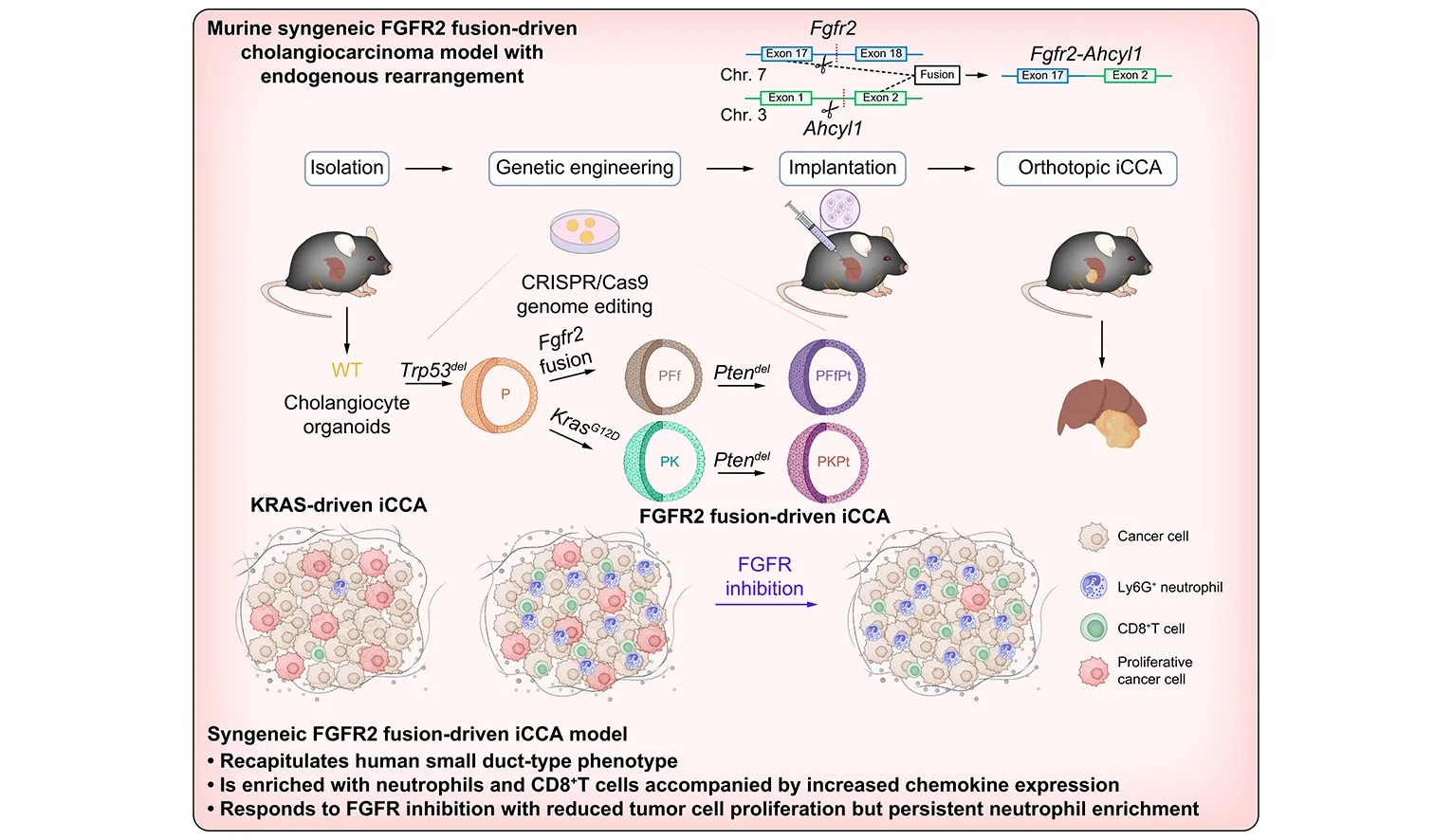

## Introduction

Intrahepatic cholangiocarcinoma (iCCA) is a primary malignancy of the liver, with a rising global incidence and mortality.^1^ The majority of patients are not eligible for curative resection at diagnosis and will eventually require palliative systemic therapy with a very poor prognosis and a 5-year-survival rate of 7–20%.^2^ Over the past decade, intense tumor genome sequencing efforts have strongly increased our understanding of the genomic landscape in iCCA, and the entity has become exemplary for routine molecular diagnostics and targeted therapy.^2^^,^^3^ Chromosomal rearrangement of the intronic region between exon 17 and 18 of the fibroblast growth factor receptor 2 (*FGFR2*) gene is among the most prevalent alterations in iCCA occurring with a frequency of 9–18%.[3], [4], [5] *FGFR2* can rearrange with dozens of diverse fusion gene partners that lead to a constitutive activation of FGFR2 signaling.^4^^,^^6^^,^^7^ Consequently, the fusion partner enables ligand-independent receptor dimerization, leading to constitutive autophosphorylation of FGFR2 chains and the activation of downstream signaling pathways, such as RAS-MAPK, JAK-STAT, and PI3K-AKT-mTOR.^8^ This oncogenic activation of FGFR2 signaling not only has cell-autonomous effects, including enhanced proliferation and invasion, but also immunomodulating and neoangiogenic effects.^8^^,^^9^ Importantly, as iCCA comprises two histomorphologically distinct subtypes, defined as large duct and small duct type iCCA, *FGFR2* fusions occur almost exclusively in the small duct type.^10^ This raises the question of whether the cell of origin underlying small duct type iCCA exerts a selective pressure favoring *FGFR2* rearrangement during malignant transformation, or whether FGFR2 pathway activation itself drives this distinct morphological phenotype.

The high prevalence of *FGFR2* fusion in iCCA has led to the successful clinical testing of FGFR inhibitors, which are now approved by the U.S. FDA and the European Medicines Agency for second-line palliative therapy. These inhibitors have shown effectiveness with objective response rates of 38% (pemigatinib) and 42% (futibatinib).^11^^,^^12^ However, these data imply that primary resistance mechanisms occur in a considerable proportion of patients; secondary resistance also remains a relevant challenge, with sustained disease control >12 months being very rare.^13^ *FGFR2* fusion commonly co-occurs in up to 63% with alterations in other genes, such as *TP53*, *BAP1*, *CDKN2A* and *CDKN2B*, *PBRM1*, and *PTEN*.^6^^,^^12^ A statistically significant association with reduced objective response and progression-free survival has only been described for *TP53* in the FIGHT-202 trial, but overall case numbers for these subtypes were low.^6^^,^^12^ Taken together, these clinical data underscore the need to improve inhibitor treatment and reduce resistance development, for example, by identifying rational synergistic therapy approaches.

Multiple oncogenic signaling pathways reportedly influence the tumor microenvironment (TME) and thus evade immunosurveillance.^14^ To what extent FGFR2 signaling affects TME in iCCA remains systematically unexplored. Preclinical models are crucial to functionally assess mechanisms within the complex intercellular interplay of TME and could help inform rational treatment combination choices to be investigated clinically. Apart from patient-derived xenografts,[15], [16], [17] three different murine models of engineered FGFR2 fusion-driven iCCA (Ff-iCCA) have been described till date.[18], [19], [20] All models rely on transgenic introduction of a human Ff transcript, and successful tumor growth has only been demonstrated in immunodeficient mouse hosts, therefore limiting the potential to investigate immunomodulatory mechanisms.

Here, we report a novel Ff-iCCA model engineered with an endogenous, murine genome-based *Fgfr2* rearrangement. This model reveals immune cell infiltration patterns driven by FGFR2 activation and offers a physiologically relevant platform to mechanistically interrogate cancer cell–TME crosstalk.

## Materials and methods

### Organoid isolation and intrahepatic implantation

Wildtype cholangiocytes were isolated from C57BL/6J mice aged 4–6 weeks, adapting a published protocol,^21^ and cultured as organoids. Genetically engineered organoid lines were then injected intrahepatically into mice via midline laparotomy under general anesthesia. All experiments were conducted in accordance with the German Animal Protection Law and approved by the Regierungspräsidium Karlsruhe (approval no. G34/22).

### Human CCA study population

Suitable iCCA tissue samples with available genetic information were identified from the Liver Cancer Center Heidelberg (LCCH) registry. Written informed consent was obtained from all patients prospectively enrolled in the LCCH registry, which was approved by the Heidelberg University ethics committee (ethical permit number S-693/2019).

Comprehensive descriptions of all materials and methods are presented in the Supplementary Material.

## Results

### Generation of endogenous FGFR2 fusion in murine cholangiocyte organoids

**Fig. 1:**
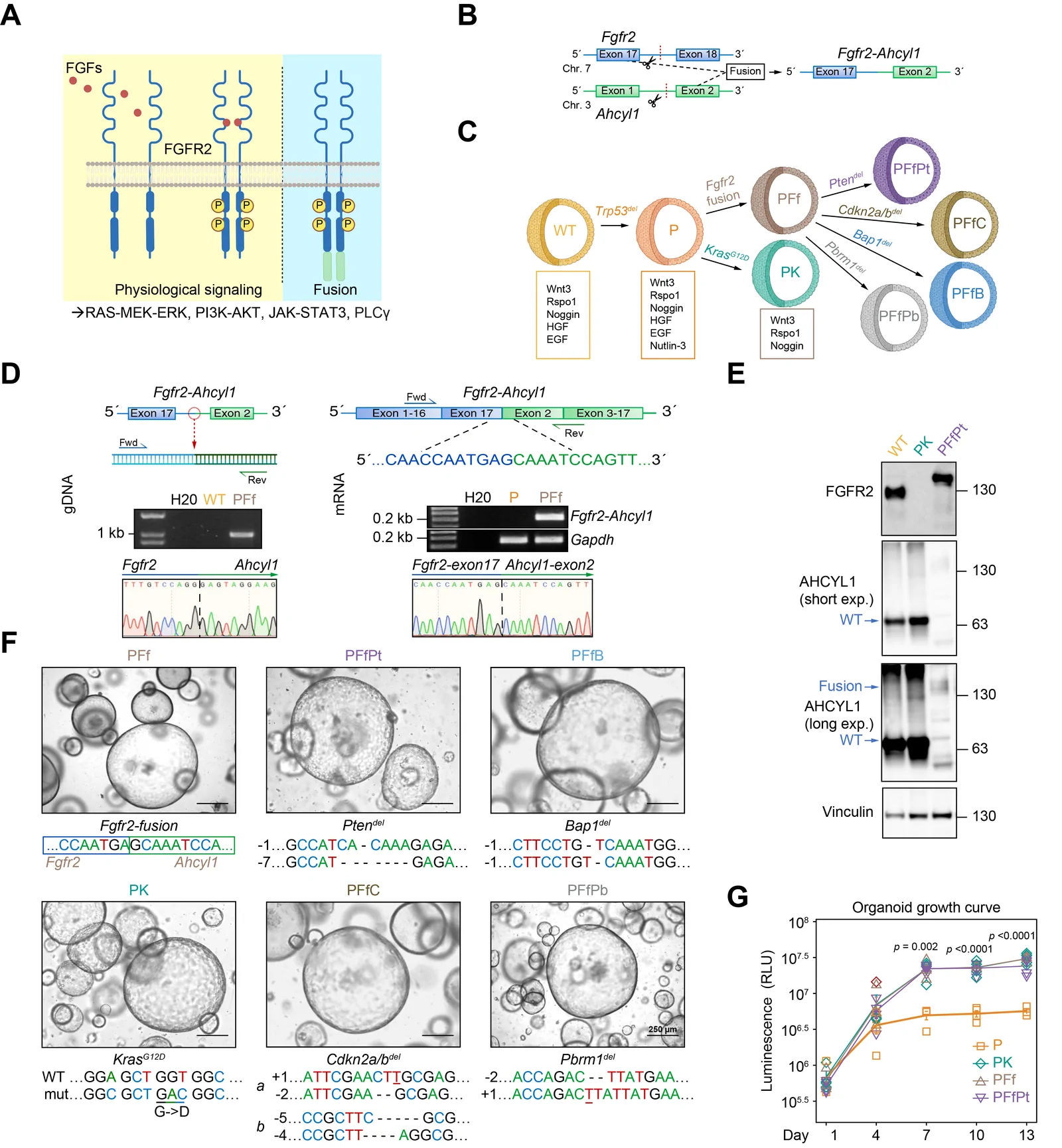
Generation of endogenous FGFR2 fusion in murine cholangiocyte organoids. (A) Illustration of FGFR2 activation physiologically or via ligand-independent receptor dimerization and autophosphorylation due to the fusion partner. (B) Schematic of the CRISPR/Cas9 intron-targeting of murine *Fgfr2* and *Ahcyl1* to generate the chromosomal rearrangement that results in the *Fgfr2-Ahcyl1* fusion gene. (C) Illustration of the sequential genome editing of wildtype murine cholangiocyte organoids via plasmid transfection to engineer FGFR2 fusion-driven CCA organoids. Abbreviations of altered genes: *Trp53* deletion (P), *Fgfr2-Ahcyl1* fusion (Ff), *Kras*^*G12D*^ (K), deletion of *Pten* (Pt), combined *Cdkn2a/b* (C), *Bap1* (B), and *Pbrm1* (Pb). (D) PCR and Sanger sequencing results of genomic DNA and mRNA to confirm the rearrangement at the expected exon-exon junction. (E) Western blot analysis of FGFR2 and fusion partner AHCYL1 indicating the respective size shift in the PFPt organoid line (exp. = exposure). (F) Microscopic bright-field images of generated CCA organoid lines as indicated, with similar morphological aspect and respective genetic alterations specified below. (G) Cell viability assay of indicated organoid lines upon withdrawal of growth factors over time. n = 4 biological replicates; the connecting line indicates mean ± SEM; significance was considered with threshold *p* <0.05; exact *p* values are shown in the figure (one-way ANOVA test to compare lines P *vs*. PFf).

To create endogenous murine *Fgfr2* fusion, we selected three common fusion partners in human Ff-iCCA (*Ahcyl1*, *Bicc1*, and *Tacc3*),^6^ and targeted the respective introns to recreate rearranged exon-exon junctions commonly occurring in humans and allowing for ligand-independent FGFR2 activation (Fig. 1A,B). Wildtype cholangiocyte organoids from C57Bl/6J mice were generated to sequentially introduce alterations able to transform them into CCA organoids (Fig. 1C). The parental organoid line harboring *Trp53* deletion (P) was used to generate a monoclonal line with *Fgfr2* chromosomal rearrangement leading to a FGFR2 fusion protein upon functional selection through withdrawal of growth factors, which was interestingly only successful with the AHCYL1 fusion partner (Fig. S1). Of the three selected fusion partners, the murine AHCYL1 protein sequence is the most highly conserved in comparison to humans with 100% homology this may explain why this fusion was particularly successful. (Fig. S2). Accordingly, the rearrangement with the expected fused sequences was confirmed on both genomic DNA and mRNA level (Fig. 1D). Detection of the FGFR2 and AHCYL1 proteins by Western immunoblotting revealed the expected shifts in size, further confirming the presence of the gene fusion (Fig. 1E).

To reflect the molecular heterogeneity of human Ff-iCCA, alterations in eight different genes were induced including *Trp53* deletion (P), *Fgfr2-Ahcyl1* fusion (Ff), *Kras*^*G12D*^ (K), *Bap1* deletion (B), *Pbrm1* deletion (P), *Pten* deletion (Pt), and combined *Cdkn2a/b* deletion (C), thereby creating six different CCA organoid lines: PK, PFf, PFfPt, PFfC, PFfB, and PFfPb (Fig. 1C,F). The selected genes were chosen based on the most common co-occurring mutations in human Ff-iCCA specimens.^5^^,^^6^ These lines were morphologically indistinguishable *in vitro* and maintained a spheric monolayer growth pattern as observed in their wildtype parental line. However, upon *Kras*^*G12D*^ mutation (line PK) or FGFR2 fusion (PFf), the cell lines continued to proliferate at a high rate despite the withdrawal of growth factors. This differed substantially from the growth rate of the cholangiocyte organoid line harboring only *Trp53* deletion, indicating the expected growth factor-independent activation of pro-proliferative pathways (Fig. 1G). In summary, we successfully created a range of cholangiocyte-derived murine CCA organoid lines based on an endogenous *Fgfr2* gene fusion.

### Murine FGFR2 fusion-driven organoids develop small duct type iCCA in syngeneic hosts

**Fig. 2:**
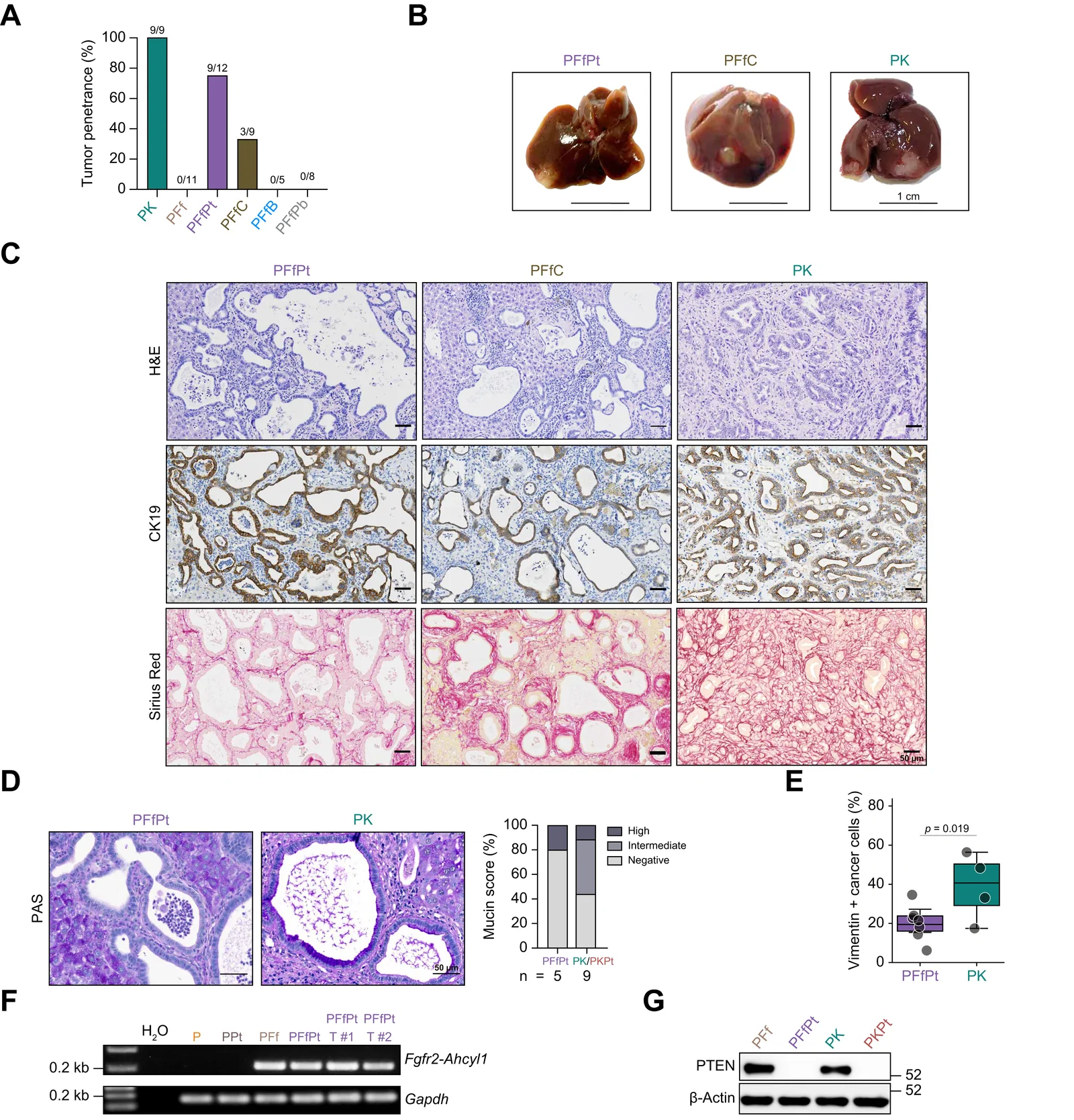
Murine FGFR2 fusion-driven organoids develop iCCA in syngeneic hosts. (A) Bar plot representing percentage of tumor penetrance for indicated lines upon intrahepatic implantation with 2–5 × 10^5^ cells for PK and 0.5–1 × 10^6^ cells for other lines (absolute tumor numbers displayed on top of the bar). (B) Representative macroscopic images of iCCA development for the respective lines. (C) Representative histologic pictures of PFfC, PFfPt, and PK iCCA with stains as indicated. (D) Representative PAS stain images of PK and PFfPt iCCA displaying mucin production at the apical membrane of PK cancer cells. Stacked bar plots of semiquantitative assessment of mucin in PFfPt and KRAS-driven (PK and PKPt) iCCA (n = 5 and 9). All scale bars indicate 50 μm. (E) Comparison of vimentin-positive cancer cell percentage. Box indicates IQR and whiskers 1.5xIQR with bold line median; each data point indicates a tumor; *p* = 0.019 (binomial generalized linear mixed-effects model on four animal replicates each). (F) Gel electrophoresis of successfully amplified *Fgfr2-Ahcyl1* mRNA by RT-PCR in original and re-cultured tumor-derived (T#1 and 2) organoid lines indicating persistent expression of the FGFR2 fusion. (G) Western blot confirming PTEN deletion in the indicated tumor-derived organoid lines.

We next systematically assessed the tumorigenic potential of each organoid line upon standardized intrahepatic implantation into the left liver lobe in syngeneic C57Bl/6J mice. The mice injected with the PK line showed rapid tumor formation (PK iCCA) within 4–10 weeks at a 100% penetrance. Conversely, the PFf line failed to induce liver tumors in this model by its own (zero of 11); of all the PFf lines harboring additional co-alterations, only PFfPt with *Pten* deletion and PFfC containing *Cdkn2a/b* deletion led to successful Ff-iCCA formation at a rate of 75% (nine of 12) and 33% (three of nine), respectively (Fig. 2A,B). Compared with PK tumors, the growth rate was slower for PFfPt and PFfC tumors, and they were typically harvested at 20–30 weeks following injection. In the mice that developed tumors, typically from one to five separate tumor nodules could be microscopically observed. A thorough histopathologic analysis revealed that all tumors were consistent with iCCA, presenting with a well-differentiated glandular growth pattern in a desmoplastic stroma reaction and a strong positivity of cancer cells for the cholangiocellular marker CK19 (Fig. 2C). Compared with PK iCCA, Ff-iCCAs showed a more pronounced glandular pattern with sometimes larger lumina, reminiscent of small duct type iCCA, and an increased accumulation of immune cells at the tumor–liver interface. Mucin production, a typical feature of large duct type iCCA, was almost exclusively prevalent in PK iCCA (Fig. 2D). The percentage of vimentin-positive cancer cells as a marker for epithelial-to-mesenchymal transition was markedly increased in PK iCCA compared with PFfPt iCCA (Fig. 2E). Upon tumor harvesting, the isolated cancer cells cultured as tumor organoids showed continuous growth in cell culture; furthermore, a sustained expression of the *Fgfr2* fusion transcript as well as PTEN knockout could be confirmed (Fig. 2F,G). Generally, these lines were stable and remained implantable after repeated freeze–thaw cycles.

For further analyses, we mainly focused on the PFfPt iCCA model in comparison with PK. To exclude that the tumorigenesis of PFfPt was primarily driven by *Pten* or *Cdkn2a/b* deletion, we created new lines that only shared *Tp53* and *Pten* or *Cdkn2a/b* deletion without *Fgfr2* fusion (PPt and PC) (Fig. S3A). Upon implantation, these lines did not result in liver tumor formation within an observation period of 20–30 weeks (zero of four and zero of six, respectively) (Fig. S3B), suggesting that these tumor suppressors are not the main oncogenic drivers in our Ff-iCCA model.

### FGFR2 fusion-driven iCCA is less immune-evasive than KRAS-driven iCCA

**Fig. 3:**
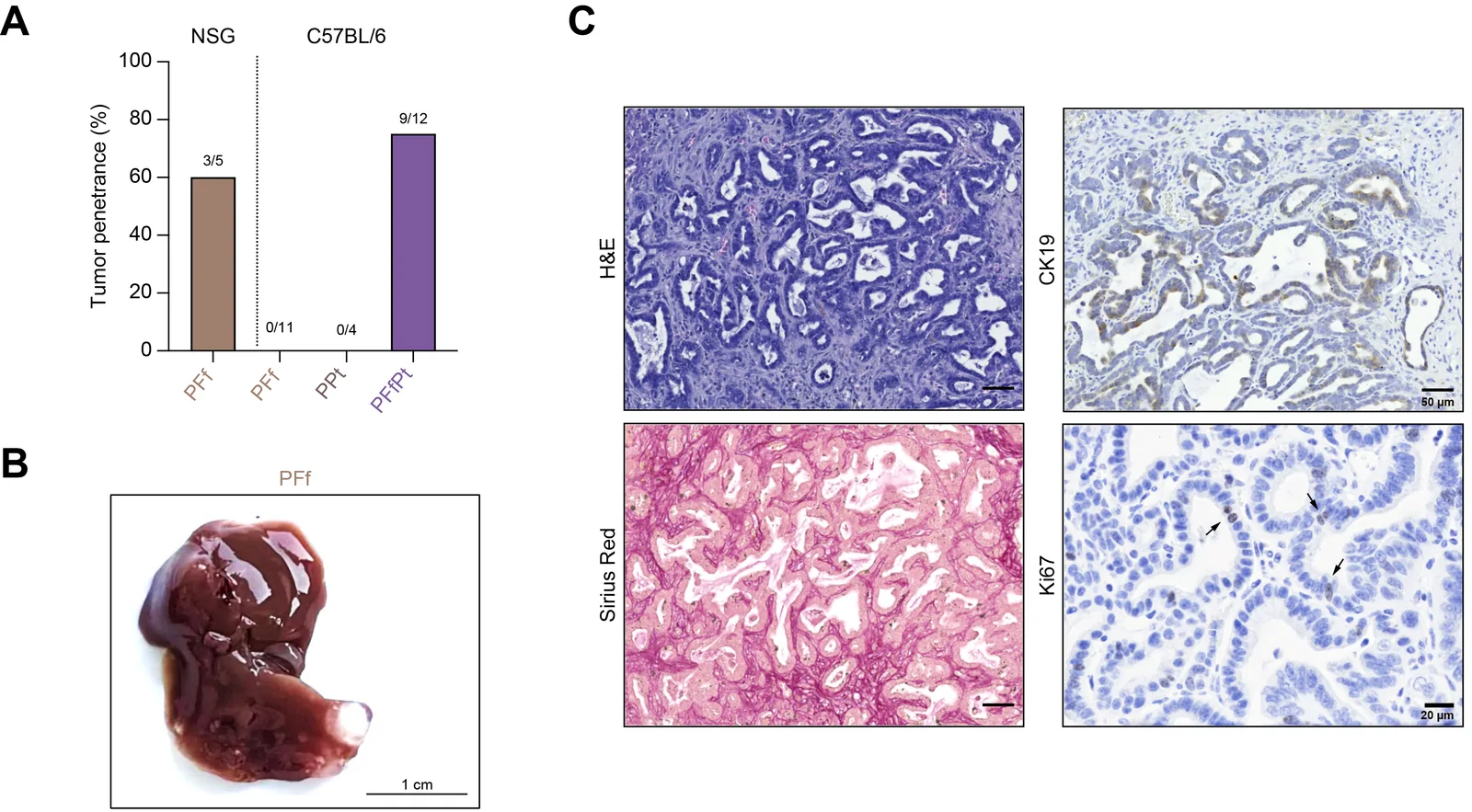
FGFR2 fusion-driven iCCA is less immune-evasive than KRAS-driven iCCA. (A) Bar plot representing percentage of tumor penetrance for indicated lines upon intrahepatic implantation; 0.5–1 × 10^6^ cells were used for implantation. (B) Representative macroscopic image of PFf iCCA in the liver of NOD scid gamma (NSG) mice. (C) Representative histologic pictures of PFf iCCA in NSG liver with stains as indicated. Black arrows indicate Ki67-positive cancer cells. Scale bars = 50 or 20 μm.

As the growth of Kras-mutated and Ff-driven cholangiocyte organoid lines in cell culture without supplements was indistinguishable (Fig. 1G), we hypothesized that either host factors or the interplay with TME was responsible for the lack of tumorigenicity of PFf organoids. Therefore, we implanted PFf organoids in the livers of immunodeficient NOD scid gamma (NSG) mice and observed tumor formation at a penetrance of 60% (3/5) within 12–20 weeks (Fig. 3A). The morphology of the cancer epithelium was comparable to the PFfPt line in wildtype C57Bl/6J mice, with a pronounced glandular growth pattern that occasionally exhibited a prominent cystic aspect; the mean proliferation rate was 3.5%, as assessed via Ki67 positivity (Fig. 3B,C).

### Multiplexed spatial TME profiling reveals oncogenic pathway-specific immune infiltration architecture

**Fig. 4:**
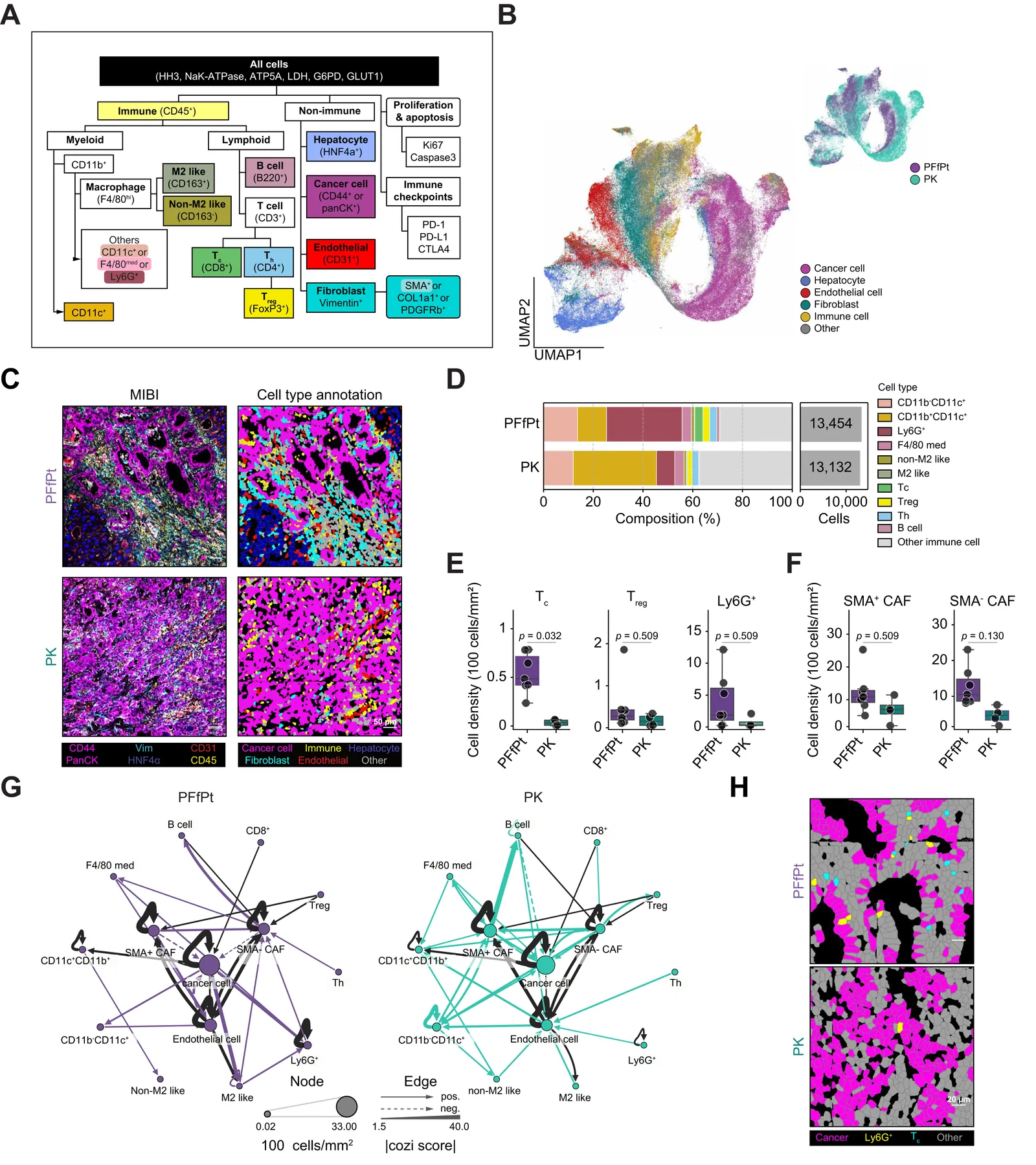
Multiplexed spatial profiling reveals oncogenic pathway-specific immune infiltration architecture. (A) Hierarchical MIBI panel overview comprising 30 antibodies targeting cellular structures, cell lineage and phenotypic markers, cell fate indicators, and immune checkpoint molecules. (B) UMAP projection of single cells based on lineage marker expression profiles. Each point represents one cell (∼248,600 cells in total from seven PFfPt (purple) and four PK (turquoise) tumors from four mice each), colored by major cell lineage annotation as indicated. (C) Representative tissue images of PFfPt and PK tumors displaying lineage markers (left) and the corresponding cell lineage annotations (right). (D) Cellular composition of the immune compartment in PFfPt and PK tumors. (E, F) Cell densities of indicated immune cell (E) and CAF (F) populations. Box indicates IQR and whiskers 1.5xIQR with bold line median; each data point indicates a tumor; exact *p* values are shown in the figure (binomial generalized linear mixed-effects model on four animal replicates each and a significance threshold of *p* <0.05). (G) Cell-type interaction networks for PK and PFfPt tumors, constructed from pairwise COZI scores (after exclusion from hepatocytes and other uninformative cell populations). Edges represent statistically significant interactions. For PK tumors, the analysis was restricted to the tumor periphery, where immune cells were predominantly enriched relative to the tumor core. (H) Representative tissue images highlighting the spatial arrangement of cancer cells, Ly6G^+^ neutrophils, and CD8^+^ T cells.

As the results from the implantation experiment in NSG mice suggest that the immune system plays a crucial role in preventing tumorigenesis of PFf iCCA, and as more immune cell presence was observed in PFfPt iCCA, we aimed at investigating the tumor immune microenvironment (TIME) in more detail. We performed multiplexed ion beam imaging (MIBI), a spatial proteomic approach, with a 30-plex antibody panel including key lineage markers for immune cells, cancer-associated fibroblasts (CAFs), endothelial cells, and cancer cells (Fig. 4A). We acquired 327 fields of view each at a resolution of 390 nm from seven PFfPt and four PK iCCA tumors from four mice, yielding a dataset of ∼248,600 cells with a mean of ∼22,500 cells per tumor (Fig. S4A). More than 80% of cells per section were confidently assigned to a defined cell type, and segmentation maps closely reflected marker intensities (Fig. S4B). Cell lineage identities were assigned hierarchically based on the lineage marker expression patterns (Fig. 4A,B and Fig. S4A and B), enabling a comprehensive analysis of TME composition.

First, we compared the two tumor types in terms of spatial organizations at the tissue level (Fig. 4C and Fig. S5A). PK iCCA tumors were larger, with a well-defined tumor border and a distinct periphery and core tissue architecture (described in detail by Liu *et al.*^22^), where immune and stromal cells were predominantly enriched in the tumor periphery. In the generally smaller PFfPt iCCA tumors, cancer cells formed slightly more cystic structures surrounded by immune and stromal cells. Within the immune compartment, CD11b^+^ CD11c^+^ dendritic cells accounted for a greater proportion in PK tumors, whereas Ly6G^+^ neutrophils and cytotoxic T (T_c_) cells were more represented in PFfPt tumors (Fig. 4D, Fig. S4C). Among immune cells, only non-M2 macrophages showed a significantly higher Ki67^+^ proportion in PK tumors (Fig. S4E). Spatial cell density analysis revealed directionally consistent differences for these populations, although only T_c_ cells reached statistical significance, likely reflecting the limited sample size (Fig. 4E). Both SMA^+^ and SMA^-^ CAFs were present at higher densities in PFfPt than in PK tumors, with the difference being slightly more pronounced for SMA^-^ CAFs, though neither reached statistical significance (Fig. 4F). Despite these compositional differences, expression levels of PD-L1 in cancer cells, PD-1 in T_c_ and regulatory T (T_reg_) cells, and CTLA-4 in T_reg_ cells were comparable between PFfPt and PK tumors (Fig. S4F), suggesting that the two tumor types share a similar immune checkpoint expression landscape.

Conversely, the two iCCA subtypes displayed strikingly divergent cellular interaction landscapes (Fig. 4G). Shared interactions in both cancer types were largely confined to stromal populations, including fibroblast and endothelial cell pairs, as well as cancer cell interactions with the immediate stromal niche. Immune and CAF interactions in PFfPt iCCA tumors were predominantly mediated by SMA^-^ CAFs, whereas SMA^+^ CAFs drove the corresponding interactions in PK tumors. Among rewired interactions between immune cells, CD11c^+^ CD11b^+^/^-^ dendritic cells acquired numerous interaction partners in PK tumors, whereas Ly6G^+^ neutrophils gained interaction partners predominantly in PFfPt tumors. With respect to cancer and immune crosstalk, T_c_ cells targeted cancer cells in both cancer types, although at markedly higher densities in PFfPt tumors (Fig. 4E,H). Cancer cell interactions were further distinguished by cancer type: in PFfPt tumors, cancer cells specifically interacted with M2-like macrophages, Ly6G^+^ neutrophils, and CD11c^+^ cells, whereas in PK tumors, cancer cell interactions involved CD11c^+^ cells in the reverse direction and F4/80^med^ myeloid cells. Collectively, Ly6G^+^ neutrophils stood out as a population differing between tumor types in both interaction connectivity and cell density (Fig. 4E,G,H and Fig, S5B,C).

### Neutrophil infiltration is not driven by *Pten* deletion

**Fig. 5:**
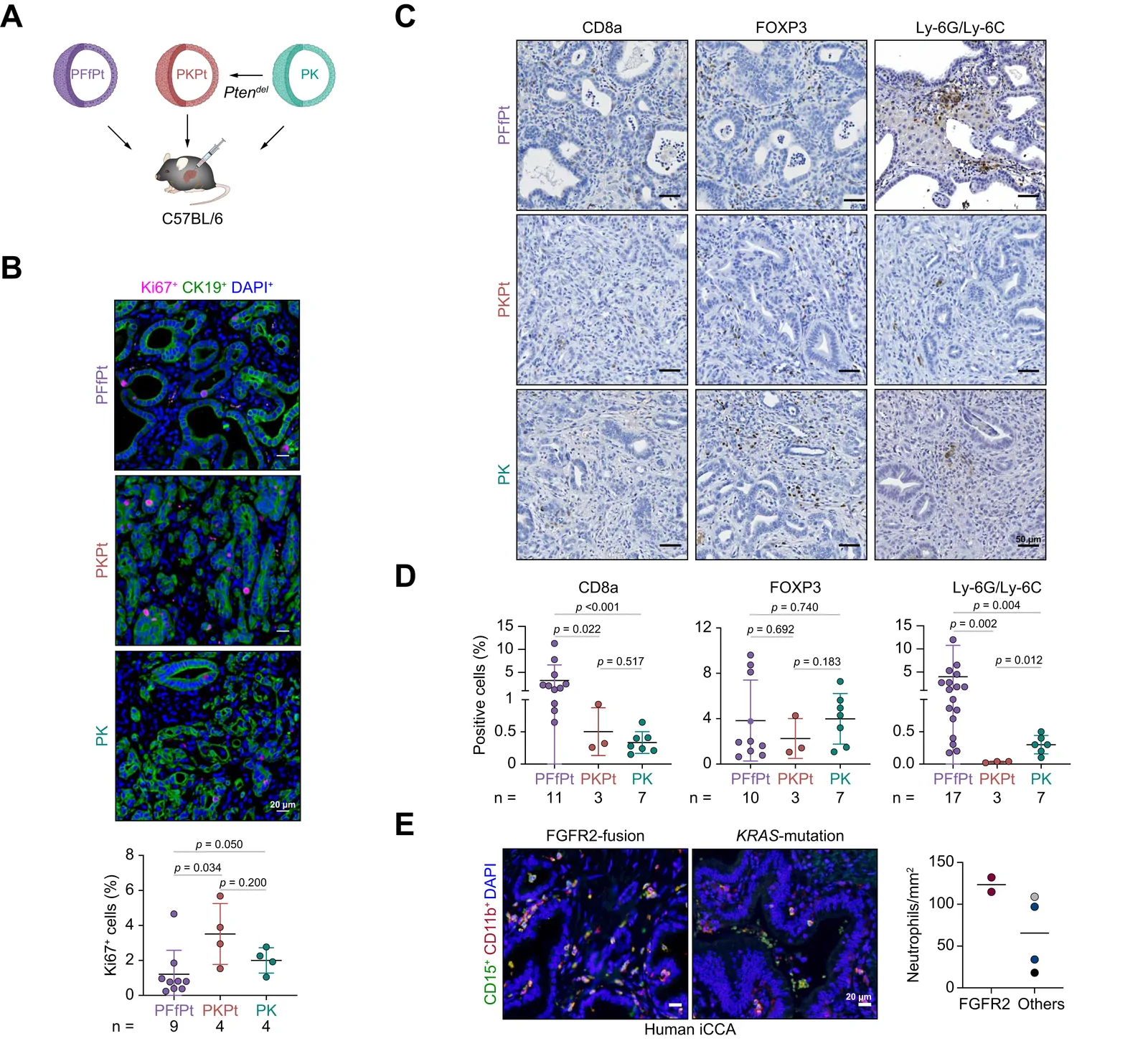
FGFR2 fusion-driven iCCA show more immune cell infiltrates than KRAS-driven iCCA. (A) Schematic illustrating iCCA models; 2–5 × 10^5^ cells were implanted for PK and PKPt lines and 0.5–1 × 10^6^ for PFfPt. (B) Representative immunofluorescence images for Ki67 and Ck19 with DAPI. Scale bars = 20 μm. Quantification of Ki67^+^ cancer cells depicted as scatter plot with each data point indicating one animal replicate: PFfPt (n = 9), PKPt (n = 4), PK (n = 5). (C) Immunostaining images for CD8a, FOXP3, and Ly-6G/Ly-6C in PFfPt, PKPt, and PK iCCA. Scale bars = 50 μm. (D) Scatter plots depicting percentage of cells positive for the indicated marker whole tumor areas per section with each data point indicating one animal replicate: PFfPt (n = 11), PKPt (n = 3), and PK (n = 7). (E) Representative immunostaining images of human FGFR2 fusion-driven iCCA and one with *KRAS* mutation. CD11b^+^/CD15^+^ cells were counted as neutrophils. Dot plot showing the mean neutrophil density of 10 high-power fields per tumor section, comparing iCCA harboring a FGFR2 fusion or other mutations. Each data point represents one tumor. Lines in plots of (B) and (D) indicate mean ± SD; exact *p* values are shown in the figure (Mann-Whitney *U* test).

We next generated a PK line harboring an additional *Pten* deletion, which readily developed iCCA upon implantation in C57Bl/6J mice. We used this line to control for PFfPt and potential immune-modulating effects of aberrant PI3K/Akt signaling in these *Pten* knockout cells (Fig. 5A and Fig. S6A–C). We first assessed the proliferation rates in all three lines upon implantation, which were significantly increased in the two KRAS-driven iCCA subtypes (mean proliferation rates of 1.2% in PFfPt, 2% in PK, and 3.5% in PKPt; Fig. 5B). Similar to our comparisons with PK iCCAs, we observed strong differences in immune cell infiltrates between PFfPt and PKPt iCCAs already by light microscopy. Consistent with the observations in the sample subset from MIBI analysis, both KRAS-driven iCCA typically showed lower infiltrates of T_c_ cells and Ly-6G/Ly-6C+ neutrophils (Fig. 5C,D). Infiltration by neutrophils has recently been identified as an exclusive TME feature in Ff-iCCA in humans.^23^^,^^24^ We aimed at independently validating this association in a retrospective analysis from the LCCH patient registry, comparing Ff-iCCA with iCCA harboring other drivers. In accordance with prior descriptions, we could observe higher neutrophil cell densities in human Ff-iCCA (Fig. 5E). Owing to low sample size, statistical testing was not applied. Thus, we could validate our MIBI-based observations of an increased infiltration of T_c_ cells and neutrophils and confirm this effect as independent of *Pten* deletion.

### PFf organoids induce a chemotaxis-enriched transcriptional program and respond to FGFR inhibitors

**Fig. 6:**
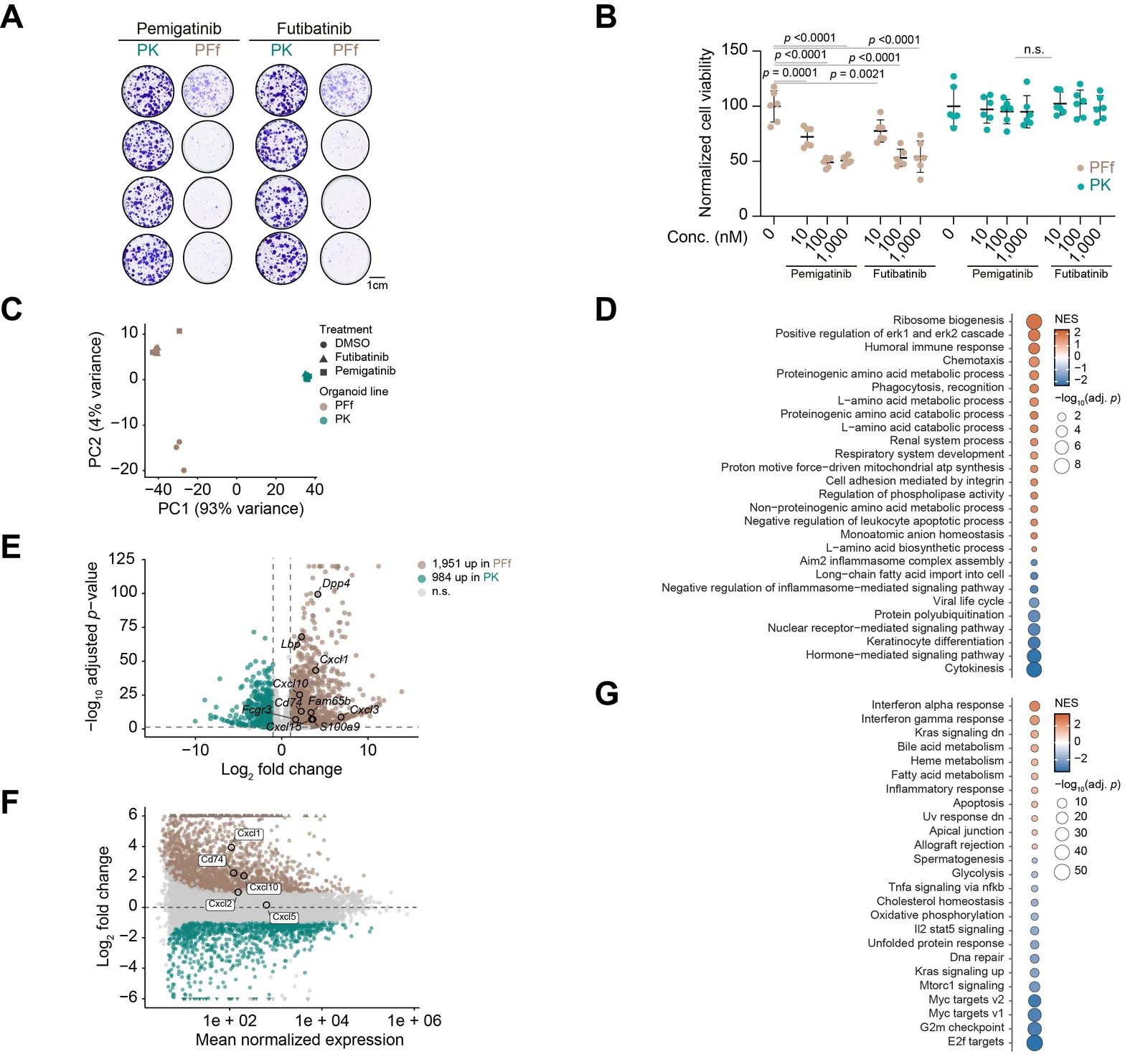
FGFR2 fusion-driven CCA organoids show an increased chemotaxis signature and are susceptible to FGFR inhibitors. (A) Representative crystal violet-stained images of wells from colony-forming assay of PFf and PK cell lines seeded as a monolayer and treated with the indicated concentrations of pemigatinib and futibatinib for 10 days. (B) Cell viability assay of PFf and PK organoids treated with the indicated concentrations of pemigatinib and futibatinib normalized to DMSO control. n = 6 per condition with line indicating mean ± SD; exact *p* values are shown in the figure, with *p* ≥0.05 considered not significant (one-way ANOVA with Welch’s correction). (C) Principal component analysis (PCA) plot of variance-stabilized expression profiles from the indicated lines and treatment conditions. PC1 (93% variance) and PC2 (4%) separate samples primarily by oncogenic pathway activation and, to a lesser extent, by FGFR inhibition (in PFf only). (D) Gene set enrichment analysis (GSEA) of Gene Ontology (GO) Biological Process terms, reduced by semantic similarity, highlights enrichment of immune and chemotaxis-related processes in PFf alongside metabolic and proliferative differences between DMSO-treated PFf and PK. (E) Differential gene expression between DMSO-treated PFf and PK shown as volcano plot of protein-coding genes ranked by log_2_ fold change and -log_10_ adjusted *p* value. Selected leading-edge neutrophil chemotaxis genes are labeled. Values above -log_10_ adjusted *p* value = 120 were capped for visualization. (F) MA plot of differential expression between DMSO-treated PFf and PK organoids, showing mean normalized expression *vs.* log_2_ fold change. Selected chemotaxis-associated genes are highlighted. Log_2_ fold changes were capped at ±6 for visualization. (G) Hallmark GSEA reveals a PFf-selective shift after FGFR inhibition, with reduced growth and cell cycle programs and relative enrichment of immune and inflammatory signatures.

The murine amino acid sequence in the kinase domain of FGFR2 shares a >99% homology with the human FGFR2 protein (Fig. S7). Accordingly, treatment with the FGFR inhibitors pemigatinib and futibatinib led to a statistically significant reduction of PFf cholangiocyte survival in a colony-forming assay in all tested dosages compared with DMSO control, whereas PK CCA cells remained unaffected (Fig. 6A and Fig. S8A). An FGFR2 fusion-specific inhibition could also be observed in a cell viability assay of these two organoid lines (Fig. 6B). The inhibitory efficacy was hereby similar to that described for some patient-derived Ff-iCCA cell lines.^15^^,^^25^^,^^26^ To explore molecular differences between PFf and PK, we performed bulk RNA sequencing across six different organoid conditions: PFf and PK organoids treated either with DMSO as a control, pemigatinib, or futibatinib (n = 3 per condition). Principal component analysis revealed that the dominant axis of variation (PC1) separated organoids by oncogenic activation (93%), whereas PC2 (4%) separated PFf, but not PK organoids by FGFR inhibition (Fig. 6C). Moreover, 3,735 genes were differentially expressed in PFf relative to PK (Fig. 6D,E and Table S4). FGFR inhibition revealed 1,742 genes with a PFf-specific transcriptional response, which we considered as *bona fide* FGFR2 target genes, including well-described targets such as *Etv4* and *Dusp6* (Table S5). Demonstrating the specificity of the FGFR inhibitors, only one gene was significantly differentially expressed in PK organoids upon treatment. Gene set enrichment analysis revealed in PFf an enrichment of basic cellular and metabolic processes (RNA/protein processing and metabolism); interestingly, the terms “humoral immune response” and “chemotaxis” ranked as the third and fourth most significant hallmarks after reducing semantic redundancy among the enriched terms (Fig. 6D). Focusing in more detail on chemotaxis-related pathway terms, all of them displayed as significantly enriched in PFf over PK, and the top 10 leading-edge genes of the “chemotaxis” gene set were all significantly overexpressed in PFf, demonstrating a robust phenotype (Fig. S8B). We next specifically investigated most common chemokines for T_c_ cells (*Cxcl9* and *Cxcl10*) and neutrophils (*Cxcl1*, *Cxcl2,* and *Cxcl5*).^27^ Among them, *Cxcl10*, *Cxcl2*, and especially *Cxcl1* were robustly and significantly overexpressed in PFf (Fig. 6F). When looking at the interaction with FGFR inhibition, to identify the key cellular programs affected via FGFR2, interferon alpha and gamma response were the two top upregulated terms, whereas expectedly cell cycle program (G/2M, E2F targets) as well as Myc activation and Kras signaling were among the top downregulated terms (Fig. 6G). Interestingly, neither “chemotaxis” term nor expression of its related genes was significantly altered upon FGFR inhibition, suggesting that the upregulation of these genes in PFf *vs*. PK cannot be explained directly via canonical signaling pathway, or that compensatory mechanisms are in play.

### FGFR inhibition in PFfPt iCCA is antiproliferative but without apparent immunomodulatory effects

**Fig. 7:**
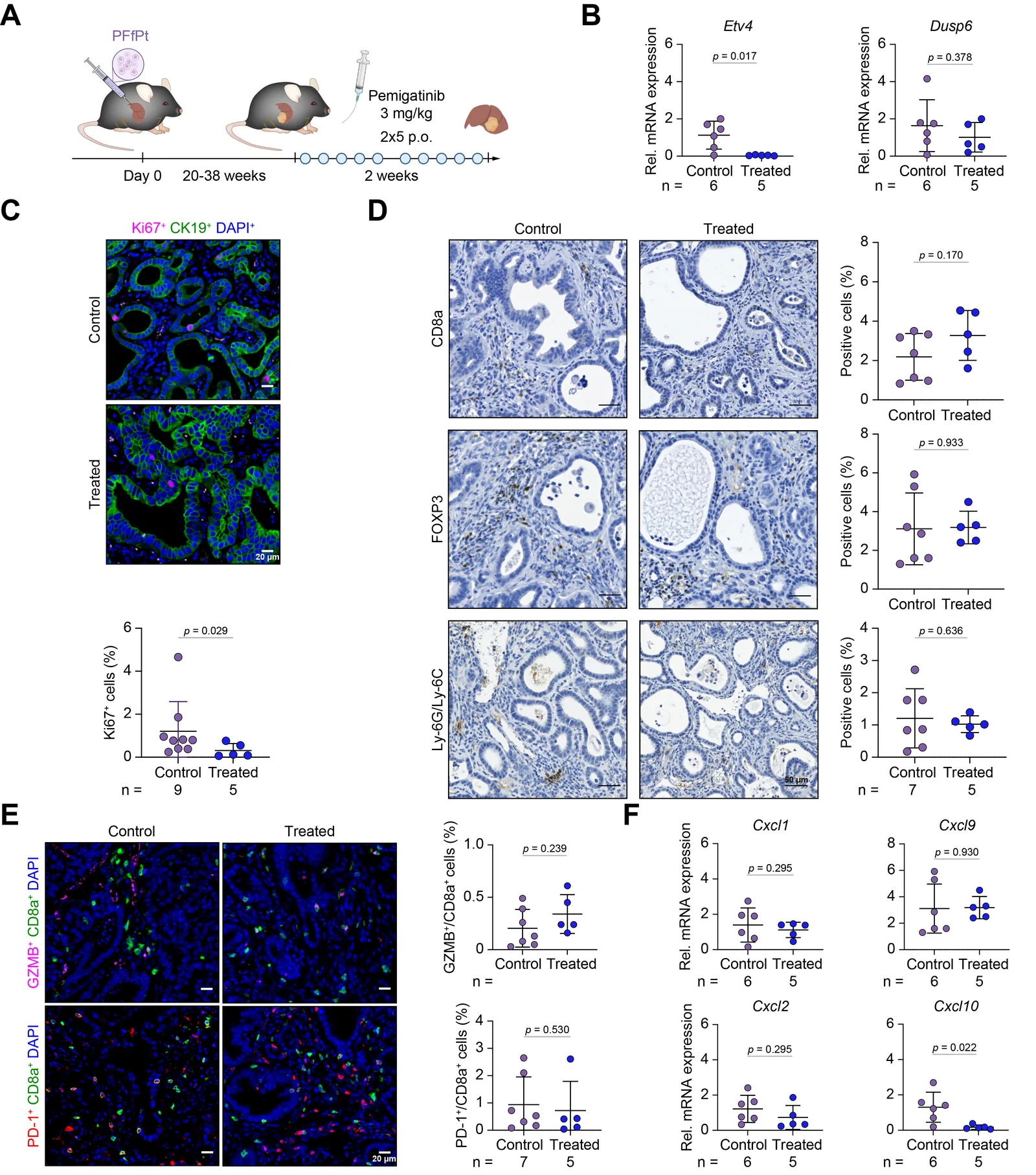
FGFR inhibition in PFfPt iCCA is antiproliferative but without apparent immunomodulatory effects. (A) Schematic of experimental treatment design. (B) Normalized FGFR2 target gene expression from control PFfPt iCCA and treated with pemigatinib. Control (n = 6), treated (n = 5); exact *p* values are shown in the figure (Welch’s *t* test). (C) Representative immunofluorescence images for Ki67 and Ck19 with DAPI. Scale bars = 20 μm. Quantification of Ki67-positive cancer cells depicted as scatter plot. Control (n = 9), treated (n = 5); *p* = 0.029 (Mann-Whitney *U* test). (D) Representative immunostaining images for CD8a, FOXP3, and Ly-6G/Ly-6C. Scale bars = 50 μm. Quantification of respective cell type abundance shown as a scatter plot. Control (n = 9), treated (n = 5); exact *p* values are shown in the figure (Welch’s *t* test). (E) Immunostaining for T_c_ cells (CD8a^+^) co-expressing either granzyme B (GZMB) or PD-1. Scale bars = 20 μm. Scatter plots depict percentage of respective double-positive T_c_ cells per mouse. Control (n = 7), treated (n = 5); exact *p* values are shown in the figure using Welch’s *t* test (GZMB) and Mann-Whitney *U* test (PD-1). (F) Intratumoral normalized mRNA expression of indicated chemokines shown as scatter plot. Control (n = 7), treated (n = 5); exact *p* values are shown in the figure (Welch’s *t* test). For all plots, each data point represents one animal as a biological replicate, and lines indicate mean ± SD.

To assess the effects of FGFR inhibition in our Ff-iCCA model, we treated mice with pemigatinib for 2 weeks once tumors had developed (n = 7 control and 5 treated; Fig. 7A). Although we observed no differences in tumor morphology or increased necrotic areas between treated and untreated PFfPt tumors, we confirmed a pharmacodynamic effect via significantly reduced proliferation rates and intratumoral downregulation of the canonical FGFR target gene *Etv4*, which was among the top downregulated genes in the bulk RNA sequencing analysis (Fig. 7B,C and Table S5). Focusing our immune infiltrate analysis on T_c_ and T_reg_ cells, as well as Ly-6G/Ly-6C+ neutrophils, we could not detect significant differences between the control and treated tumors (Fig. 7D). In parallel, there was also no evidence of increased T_c_ cell activity or checkpoint engagement based on granzyme B or PD-1 positive T_c_ cell assessment upon FGFR inhibition (Fig. 7E). Consistent with our results from the bulk RNA sequencing on treated PFf organoids and the observed lack of immune infiltration patterns upon FGFR inhibition, the mRNA expression of most chemokines we assessed as relevant for T_c_ cell and neutrophil attraction were intratumorally not changed (Fig. 7F). The only robustly downregulated chemokine was *Cxcl10*, an effect we did not observe in PFf organoids *in vitro* and potentially explained via another cellular source in TME. In summary, FGFR inhibition *in vivo* leads to measurable cancer cell-specific effects in this endogenously engineered murine FGFR2 fusion model. However, we did not observe any major changes in neutrophil and T-cell infiltration and activity as a surrogate for an immunomodulatory effect.

## Discussion

Here, we introduce a new organoid-based iCCA preclinical model that allows us to reproduce co-alterations frequently observed in human iCCA through systematic genetic engineering. This approach offers versatility and uses cholangiocytes as the cell of origin in contrast to many widely used iCCA models relying on transforming hepatocytes via transposon- and viral vector-based systems.^28^ Typically, these systems also have to rely on oncogenes that drive towards biliary lineage, which limits the range of alterations that can be explored. The organoid-engineering approach used here profits from genetic flexibility with additive mutations, as first described in human cholangiocyte organoids.^29^ However, it requires orthotopic implantation and, therefore, may be less suitable for assessing the aspects of early carcinogenesis compared with autochthonous models.

Since the first conception of small duct and large duct type iCCA based on pathomorphological criteria in 2010,^30^ they have become widely accepted as biologically distinct and prognostically relevant entities, now incorporated into the 5th WHO classification and clinical practice guidelines.^31^ The genomic landscape of iCCA has further strengthened this classification by reflecting the enrichment of different driver mutations.^10^^,^^31^ *FGFR2* rearrangements are considered exclusive to small duct type iCCA, whereas KRAS-driven iCCA is enriched in the large duct type.^10^ Consistently, our model recapitulates these hallmarks in the respective cancer genotypes. Mucin production, for example, was almost exclusive to the KRAS-driven iCCA, which was overall the more aggressive tumor model. The prevailing hypothesis to explain the distinction of small and large duct type iCCA holds that they arise from different cells of origin reflecting the morphology and function of cholangiocytes in small *vs.* large ducts. This would in turn imply that these originally transformed cells have a propensity for specific genetic alterations, such as *FGFR2* rearrangements or *KRAS*-mutations, respectively. Interestingly, because our iCCA subtypes derive from the same parental cholangiocyte, our findings suggest that specific pathway activation alone can influence the pathomorphological phenotype. Interpretation is nonetheless limited, as transformation occurs in cholangiocyte organoids cultured *in vitro*, outside their physiological niche, and given the known plasticity of hepatobiliary epithelium.^21^^,^^32^ Nevertheless, we strongly encourage further investigation into the relationship between cell of origin and oncogenic pathway activation in small and large duct type iCCA.

By achieving an endogenous rearrangement of *Fgfr2* in the mouse genome, our model closes an important gap in Ff-iCCA preclinical modelling as it enables investigations in a syngeneic system and detailed analysis of the tumor and TIME interplay. Although it seems theoretically advantageous to systematically study common co-occurring mutation patterns, such as *Bap1*, *Pbrm1*, *Cdkn2a/b*, and *Pten*, our efforts in this regard were substantially hampered by the lack of tumorigenicity of lines with co-deletion of *Bap1* or *Pbrm1*, and by variable penetrance rates (33–75%) in lines with co-deletion of *Cdkn2a/b* or *Pten*. Our *in vitro* data did not hint at any cell autonomous disadvantage compared with *Kras*^*G12D*^-mutation driven iCCA lines; similarly, proliferation rates were only slightly increased in our KRAS-driven iCCA tumors. As PFf cholangiocyte organoids could grow in NSG mice, the key hurdle to overcome in our model may be antitumor immunity.

The increased immunogenicity of Ff-iCCA could be due to the neoantigen presentation of the fusion protein epitopes, which has been demonstrated in at least one human case.^33^ However, it is more likely that oncogenic KRAS signaling, which was our standard comparative model in this work, has superior mechanisms for immune escape. *Kras*^*G12D*^ mutation-harboring lines had a higher tumor penetrance and possibly generated very immune-excluded, *i.e.* “immunologically cold” iCCAs in our model. Differences in tumor growth kinetics or penetrance represent a possible alternative explanation for this finding. However, this is unlikely because our KRAS-driven iCCA model showed consistently low immune infiltrates across multiple time points, whereas genetic manipulation of KRAS effector genes was sufficient to reverse this low-infiltrate phenotype independent of changes in growth kinetics.^22^ This immune-excluded phenotype is consistent with findings in human CCA, where co-occurrence of *Trp53*-and *Kras*-mutations were mostly associated in low immune enrichment classes (*i.e.* tumor classical, and desert-like).^34^ Moreover, several immune escape mechanisms have been described for oncogenic KRAS signaling. These include the secretion of chemokines and cytokines to create an immunosuppressive TME, or the repression of interferon signaling, which may be relevant in our model.^35^^,^^36^

Ff-iCCA is a frequent CCA subtype amenable to targeted therapy. Although a subset of patients can profit from FGFR inhibitors with objective response rates observed in up to 42%, the duration of response is usually limited to less than a year and many patients remain primary non-responders.^11^^,^^12^ Secondary resistance is mainly attributed to the acquisition of resistance mutations in the *FGFR2* gene,^6^^,^^7^^,^^37^ but the reason for the variability of initial response remains unclear. With genomic data from the FIGHT-202 and FOENIX trials, comprehensive analyses of the prognostic value of co-mutations provided few insights.^6^^,^^7^ One limitation is clearly that such analyses could be underpowered, particularly when looking at possibly weak effects of different co-alterations of a subtype of a relatively rare cancer. However, it may also reflect the actual predictive value of the cancer genome by itself, as it insufficiently informs on the downstream biological effects and the associated host responses that could influence treatment response. This is of particular importance when trying to develop combination treatment including immunotherapy strategies.

Interestingly, of all co-alterations tested in our Ff-iCCA model, additional *Pten* deletion was most successful to overcome this obstacle to tumor formation. Because *Pten* knockout itself is not a main oncogenic driver in our model, it is likely that this co-alteration supports immune escape mechanisms. *Pten* deficiency reportedly leads to an immunosuppressive TME and is associated with various immune-evasive pathways, such as reducing T-cell infiltration, interferon response, or activating STAT3-mediated signaling via PI3Kβ,[38], [39], [40] effects which could be reversed pharmacologically with PI3Kβ inhibitors or re-introduction of PTEN via lipid nanoparticles.^39^^,^^41^ PTEN co-alterations in Ff-iCCA are not the most common ones with a frequency of ∼3–5%,^5^^,^^6^ but a combinatory targeted approach could be worth exploring in this subgroup.

One of the major phenotypes observed in our Ff-iCCA model is the strong infiltration of myeloid cells positive for Ly6G/C, a classic marker for neutrophils. Most importantly, neutrophil infiltration has very recently been identified as an exclusive TME feature in Ff-iCCA in humans, a trend that was similarly observed in our own human cohort.^23^^,^^24^ The association of neutrophil infiltration with human Ff-iCCA has yet to be functionally proven. Our transcriptome analysis revealed that an increased expression of common chemotaxis genes was one of the most distinct features of PFf *vs.* PK CCA organoids. However, FGFR inhibition did not affect their expression levels; accordingly, we did not detect a difference in neutrophil infiltration upon pemigatinib treatment *in vivo*. Interestingly, FGFR inhibition in PFf led to a significant upregulation of interferon-stimulated genes. These findings are consistent with a recent report finding that activated FGFR signaling is blocking the Jak-STAT pathway downstream of IFNγ in renal cell carcinoma, which can be reactivated upon treatment with lenvatinib, a receptor tyrosine kinase inhibitor with partial anti-FGFR activity.^42^ Given that interferon signaling is among the most potent inducers of chemotaxis genes,^43^ whether such a compensatory mechanism could explain the persistently high expression of chemotaxis genes in Ff-iCCA despite FGFR inhibition remains to be investigated. Taken together, although our results cannot establish a definitive causal relationship between FGFR2 signaling and neutrophil recruitment, they confirm this strong association in an immunocompetent preclinical model and we encourage further investigation.

In summary, we have generated a syngeneic Ff-iCCA model that allows the functional study of cancer cell and TIME interplay. Our tumor model recapitulates key morphologic features of human Ff-iCCA and reproduces a neutrophil-enriched TME. Notably, this neutrophil association was not acutely reversed by FGFR inhibition. How oncogene-specific cancer cell programs establish and sustain this functional state of the TIME, and whether it can be therapeutically modulated, remain open questions that this model is positioned to address.

## Abbreviations

CAFs, cancer-associated fibroblasts; Ff-iCCA, FGFR2 fusion-driven iCCA; FGFR2, fibroblast growth factor receptor 2; iCCA, intrahepatic cholangiocarcinoma; LCCH, Liver Cancer Center Heidelberg; MIBI, multiplexed ion beam imaging; NSG, NOD scid gamma; T_c_ cells, cytotoxic T cells; TIME, tumor immune microenvironment; TME, tumor microenvironment; T_reg_ cells, regulatory T cells.

## Authors’ contributions

Conceptualization: MTD. Methodology: NL, TKD, AO, YLW, CSL, MTD. Validation: NL, TKD, AO, YLW, CSL, MTD, AS, TL. Formal Analysis: NL, TKD, AO, YLW, CSL, DK. Investigation: NL, TKD, AO, YLW, CSL. Resources: AS, FJH, TL, MTD. Writing – original draft: NL, MTD. Writing – review and editing: NL, TKD, AO, YLW, CSL, DK, AS, FJH, TL, MTD. Visualization: NL, TKD, AO, YLW, CSL, MTD, DK. Supervision and project administration: MTD. Funding acquisition: NL, AO, YLW, FJH, MTD.

## Data availability

The bulk RNA sequencing data generated in this study have been deposited in the European Nucleotide Archive (ENA) under accession number (PRJEB113177) and the MIBI spatial proteomics data have been deposited in the BioImage Archive under accession number (S-BIAD2557).

## Declaration of generative AI and AI-assisted technologies in the manuscript preparation process

During the preparation of this work, the authors used Anthropic Claude and ChatGPT for copy editing to check grammar and spelling and improve language and readability. After using this tool, the authors reviewed and edited the content as needed and take full responsibility for the content of the publication.

## Financial support

NL was supported by a Heinrich F. C. Behr scholarship. YLW was supported by a DKFZ Postdoctoral Fellowship. This work was supported by the Clinician Scientist Program of the Medical Faculty of Heidelberg University to AO; by the Initiative and Networking Fund of the Helmholtz Association under the Helmholtz Young Investigator Program (VH-NG-1605), the European Commission (ERC Starting grant (101116823), the State Parliament of Baden-Württemberg for the Innovation Campus Health + Life Science Alliance Heidelberg Mannheim, the Rolf M. Schwiete Stiftung, the Hector Foundation, and the Deutsche Forschungsgemeinschaft (DFG, German Research Foundation) under Germanys Excellence Strategy – EXC-3018/1 – 533587280 to FJH; the Rolf M. Schwiete Stiftung, and a Max Eder grant (70113858) from the German Cancer Aid to MTD.

## Conflict of interest

The authors declare no conflicts of interest that pertain to this work.

Please refer to the accompanying ICMJE disclosure forms for further details.
