# Supplementary material for: FGFR2 fusion-driven cholangiocarcinoma is characterized by a distinct neutrophil-enriched tumor microenvironment in a syngeneic murine model
Source: JHEP Rep. 2026 Jul 21;8(10):101964. doi: 10.1016/j.jhepr.2026.101964 (PMC13598188; doi:10.1016/j.jhepr.2026.101964)
Supplement: Supplementary file 1 [file mmc1.pdf]

FGFR2 fusion-driven cholangiocarcinoma is characterized by a distinct neutrophil-enriched tumor microenvironment in a syngeneic murine model

Nugzar Lekiashvili, Trinh Kieu Dinh, Alexander Olkus, Yu-Le Wu, Chun-Shan Liu, Daria Komkova, Albrecht Stenzinger, Felix J. Hartmann, Thomas Longerich, Michael T. Dill

Table of contents

Supplementary Materials and Methods…………………………...………..…………2
Supplementary References. ……………………………………………………….18
Supplementary Figures………………………………………………...……...……...21
Table S1. …………………………………………………………………………….31
Table S2.……..…..……………………………………………………………………33
Tables S3-5…………………………………………………......…separate excel files

Supplementary Materials and Methods

Organoid media generation and composition

Organoid media consisted of a 1:1 mixture of L-WRN conditioned media generated as described previously,[1] and advanced DMEM/F12 (Thermo Fisher Scientific, #12634010) supplemented with 1% (vol/vol) Glutamax (ThermoFisher Scientific, #35050038), 10 mM HEPES (ThermoFisher Scientific, #35050038), 1:50 B27 supplement (without vitamin A) (ThermoFisher Scientific, #12587010), 1:100 N2 supplement (ThermoFisher Scientific, #17502048), 10 mM Nicotinamide (Sigma-Aldrich, #N0636), 50 ng/ml rmEGF (ThermoFisher Scientific, #31509), 50 ng/ml rhHGF (ThermoFisher Scientific, #10039-500), 1% (vol/vol) penicillin/streptomycin (ThermoFisher Scientific, #15140122), and 100 µg/ml Primocin (Invivogen, #ant-pm1) and - upon initial derivation and upon passage - 10 µM Y27632 (Holzel, #TMO-T1725-50mg).

Murine cholangiocyte isolation and organoid culture

Wild-type cholangiocytes were isolated from 4-6 week-old C57BL/6J mice, adapting a published protocol.[2] In short, after euthanization via cervical dislocation, a midline laparotomy was performed and mouse livers immediately perfused via portal vein cannulation by a pre-warmed (37°C) perfusion buffer (50 mM EDTA, 10 mM HEPES in 1X HBSS [GIBCO]) at 6 mL/min for 2 min, then 8 mL/min for another 2 min, followed by collagenase IV buffer (Collagenase IV:150 U/mL, Invitrogen, #17104019; in 1X HBSS with 1.25 mM $CaCl_2$, 4 mM $MgCl_2$, 10 mM HEPES) for 5–10 min at 8 mL/min. After removal of the gallbladder, the liver capsule was removed and the tissue gently agitated to remove hepatocytes. The remaining liver scaffold underwent sequential digestions with collagenase IV buffer (150 U/mL), accutase, and 0.25% trypsin for 30, 30, and 20 min respectively at 37°C. Cells were collected after each step, filtered (100 µm), washed, and resuspended in ice-cold resuspension buffer (2% FBS, 1.25 mM $CaCl_2$, 4 mM $MgCl_2$, 10 mM HEPES, 5 mM glucose in 1X HBSS). The suspensions were then combined and spun at 50 G for 5 min at 4°C. The supernatant was then transferred and spun at 300 G for 5 min at 4°C. The cell pellet was then resuspended in

basement membrane extract (BME, Cultrex Reduced Growth Factor Basement Membrane Extract, Type 2, R&D Systems, #3533-010-02) and plated in 24-well plates (50 µl droplet per well). After solidification, BME droplets were overlaid with 500 µl of organoid media.

After 7-14 days, outgrowing cholangiocyte organoid clones were picked for further passage. For expansion and passaging, organoids were mechanically disrupted with a 25G needle on a 1ml syringe, the cell-suspension supplemented with fresh ice-cold DMEM and spun at 300 G for 5 min at 4°C. The cell pellet was resuspended in fresh BME. Media was changed two to three times per week. Bright-field images of organoids were taken with a DM IL LED Inverted Laboratory Microscope (Leica).

Cloning of sgRNA constructs

For single guide (sgRNA) constructs, the pX458 pSpCas9(BB)-2A-GFP and pX459 pSpCAs9(BB)-2A-Puro V2.0 backbone plasmids were used (Addgene, #48138 and #62988).[3] The pX458-sgRNA(*Trp53*) plasmid was constructed by integrating the published *Trp53* sgRNA oligos into the pX458 vector as described.[4] The sgRNA(*Kras*)-*KrasG12D*_HDR plasmid was generated by modifying the parental plasmid AAV-KPL (Addgene #60224).[4] Briefly, AAV-KPL was digested with BamHI and KpnI to excise the additional sgRNA cassette. The linearized backbone was blunt-ended by Klenow and subsequently ligated to re-circularize the plasmid. Additional sgRNA were designed using Benchling (Biology Software, 2022) and the SYNTHEGO CRISPR design tool (https://design.synthego.com) and oligos cloned into the pX458 or pX459 vector. All oligo sequences are listed in **Table S1**.

Genetic engineering of monoclonal organoid lines

For transfection, organoids were dissociated by incubating with 500 µL/well of Cell Recovery Solution (Corning. #354253) on ice for 20 min, followed by centrifugation at 500 G for 5 min at 4°C. The cell pellets were resuspended in TrypLE (Thermo Fisher Scientific, #12605010) at RT for 5-8 min with intermittent shaking, then

mechanically dissociated with a 25G needle. After quenching the digestion reaction with ice-cold DMEM and centrifugation, cells were resuspended in organoid medium with 10µM of Y-27632 (TargetMol, cat#TMO-T1725-50mg) and plated at 450 µL/well in a 48-well plate. For transfection, Lipofectamine 2000 (Invitrogen, cat# 11668019) mixed with plasmid DNA (1.5 µg/reaction) was prepared according to manufacturer's instruction and added dropwise to the cells. Cell plates were centrifuged at 600 G for 60 min at 32°C, then incubated at 37°C for 4 hours. Cells were collected, centrifuged (845 G, 5 min, 4°C), resuspended in BME, and seeded in 50 µl droplets into a 24-well plate. Organoid media was added after solidification of the BME.

To generate cholangiocyte organoids carrying *Trp53* deletion, wildtype organoids were transfected with pX458-sgRNA(*Trp53*) plasmid and treated with 7.5 µM Nutlin-3 (Biotrend #A4228-S) starting 2 days post-transfection for functional selection. Outgrowing clones were picked between 14-20 days after transfection. To generate cholangiocyte organoids carrying a $Kras^{G12D}$ mutation, cells were co-transfected with pX459-(BB) and sgRNA(*Kras*)-$Kras^{G12D}$_HDR plasmids. The day after transfection, 1 µM of Alt-R™ HDR Enhancer V2 (IDT, #10007921) was added for 24 hours to promote homology-directed repair. On day 3, cells were treated with 1 µg/mL puromycin (Sigma-Aldrich, # P9620-10ML) in organoid media for 2 days to select for transfected cells. Beginning 3 days post-transfection, cells were switched to organoid media lacking mEGF and hHGF supplemented with 1 µM gefitinib (Biotrend, #A8219-S) for functional selection. Outgrowing clones were picked approximately 14 days after transfection.

The generation of *Fgfr2* rearrangements was done in a similar fashion with co-transfection of organoids with pX459-sgRNA(*Fgfr2*) and either pX459-sgRNA(*Ahcyl1*), pX459-sgRNA(*Bicc1*), or pX459-sgRNA (*Tacc3*) template, with selection with puromycin and functional selection under growth factor withdrawal as described for $Kras^{G12D}$. Outgrowing clones were picked approximately 5-7 days after transfection (**Fig. S1**). *Pten*-, *Ckdn2a/b*-, *Bap1*- and *Pbrm1*-deleted organoids were generated by transfection with pX459 plasmid with the respective sgRNA,

treated with 1 µg/mL puromycin in organoid media on day 3-4 and outgrowing organoids picked to generate monoclonal lines and screened for deletion.

To confirm successful genome editing of the picked organoid clones, genomic DNA was isolated with Quick-DNA Miniprep Kit (Zymo Research) and the targeted genomic region amplified by a standard PCR protocol using 2X Taq Master Mix (Genaxxon, #3029) and the primers indicated in **Table S1**. PCR products were verified by Sanger sequencing (Microsynth AG, Balgach, Switzerland). Indel mutation frequencies were analyzed using EditCo's ICE Analysis tool (https://ice.editco.bio/#).[5] For $Kras^{G12D}$ mutation in chol-PK, PCR amplicons were further confirmed by TA-cloning (PCR Cloning, NEB, cat#E1203S) and Sanger sequenced.

Lentivirus production and transduction

The Lenti-luciferase-P2A-Neo plasmid (Addgene #105621) was co-transfected with packaging plasmid psPAX2 and pMD2.G (PlasmidFactory GmbH & Co. KG, Bielefeld, Germany) into HEK293T cells by TransIT LT1 transfection reagent (Mirus Bio, Madison, WI, USA) according to the manufacturer's instructions. The supernatant containing lentivirus was harvested 2 days after transfection, filtered, and stored at -80°C until usage. Organoids were infected with the lentivirus supernatant and selected by 500 µg/ml G418 (ThermoFisher Scientific, #10131027) for at least 2 weeks.

RNA extraction and reverse transcription quantitative PCR

RNA from cells or tumors was isolated with Direct-zol RNA Microprep kit (Zymo Research, #R2062) with on-column DNase I digestion according to manufacturer's instructions. RNA concentration was measured using NanoDrop (Thermo Fisher Scientific, Waltham, MA, USA).

RNA extraction from FFPE sections was performed as previously described.[6] Briefly, tumor areas were marked on H&E-stained slides and corresponding tissue areas were macrodissected, marking the tumor area and scratching material from subsequent unstained 5 µm thick sections. RNA extraction was conducted automatically using the Maxwell RSC FFPE RNA Kit on

a Maxwell RSC Benchtop Instrument (Promega, Madison, WI, USA), in accordance with the manufacturer's instructions. The RNA concentration was measured fluorometrically (QuBit 2.0 RNA high sensitivity kit, Thermo Fisher Scientific, Waltham, MA, USA) following the manufacturer's instructions.

Reverse transcription was carried out using iScript cDNA Synthesis kit (Bio-Rad, # 1708891) with 500ng or 1µg RNA as template and cDNA amplified using 2X Taq Master Mix (Genaxxon, #3029) with target region-specific primers (**Table S1**). Quantitative polymerase chain reaction (qPCR) was performed with 384-well or 96-well plates using 10ng cDNA input and Luna Universal qPCR Master Mix SYBR Green (New England Biolabs, #M3003E) with gene specific custom made primers (Sigma Aldrich) (**Table S1**) on a Quant Studio 6Pro Cycler (Therma Fisher Scientific, Waltham, MA, USA) or a CFX-Maestro (BIO-RAD, Feldkirchen Germany), respectively.

Initial denaturation was performed for 10s at 95°C. Amplification was performed in a two-step cycle for 15s at 95°C followed by 1 min at 60°C. Primers were evaluated by analysis of melting curves and amplification size of the PCR products using 1.5 % agarose gel electrophoresis. The primer efficiency was tested by serial dilution. Relative mRNA expression was analysed using the ΔΔ-CT method by normalisation to the housekeeping genes (glyceraldehyd-3-phosphat-dehydrogenase (GAPDH) or ribosomal protein 14 (RPS14).

Western blotting

Organoids and 2D cells were starved for 48 hours in basal media without supplements before protein extraction. For lysis, 4–5 confluent 50 µl BME domes were processed using Cell Recovery Solution (Corning #354253) and ice-cold DMEM. Cell pellets were lysed in RIPA buffer (ThermoFisher Scientific, #89900) supplemented with phosphatase inhibitor (ThermoFisher Scientific, #89900) and protease inhibitors (Hölzel Diagnostika, # HY-K0011-25pcs), incubated on ice for 30 min, and centrifuged at 16'000 G for 20 min at 4°C. Protein concentrations were determined using the BCA assay (ThermoFisher Scientific, #23225). 20 µg of protein were mixed with Laemmli buffer (Alfa Aesar, #J61337-AC), boiled, and

separated by SDS-PAGE using 7.5% TGX™ gels (Bio-Rad, #1610180). Proteins were transferred to 0.45 µm PVDF membranes (Millipore, # IPVH00010) overnight at 10 mA in cold conditions. Membranes were blocked in 3% BSA (Sigma-Aldrich, # A7030-100G) and incubated with primary antibodies overnight at 4°C (**Table S2**). After washing, HRP-conjugated secondary antibodies were applied, and chemiluminescent signals (Millipore, WBKLS0500) were developed using an Amersham Imager 680. For reprobing, membranes were stripped in glycine-based stripping buffer (3% glycine, 0.2% SDS and 2% Tween 20 at pH 2.2) and re-blocked.

Organoid viability assay

Organoids were mechanically and enzymatically dissociated as described above and seeded 1200 cells in 10 µl BME per well in white 96-well microplates. After BME solidification, 100 µl of organoid media without mEGF and hHGF was added. Media was renewed every 3rd day. Cell viability was assessed at the indicated time points using CellTiter-Glo® 3D Cell Viability Assay (Promega, #G9683), according to the manufacturer's instructions. Growth curves are presented as mean of 4 biological replicates, and each biological replicate contains 2 technical replicates.

Animal experiments

C57BL/6J mice were obtained from Janvier Labs and NOD.Cg-*Prkdc*^{scid} *Il2rg*^{tm1Wjl} (NOD scid gamma, NSG) internally from the German Cancer Research Center (DKFZ). All mice were housed under specific pathogen-free (SPF) conditions in individually ventilated cages on a 12-hour light/dark cycle at 21–23 °C, with ad libitum access to food and water. All experiments were conducted in accordance with German law and approved by the Regierungspräsidium Karlsruhe (approval no. G34/22). For orthotopic tumorigenicity assays, mice older than 8 weeks were used. Mice were anesthetized with isoflurane (0.5-1% with oxygen), a small midline laparotomy and intrahepatic implantation via 28-gauge syringe into the left liver lobe was performed. The injection site was then compressed with a cotton-top to avoid leakage or bleeding, and peritoneum and skin closed with absorbable suture

(Ethicon, VCP303H) and 9 mm clips (Fine Science Tools, 12022-09), respectively. Proper peri- and postoperative analgesia was applied with metamizole and lidocaine. For implantation, typically 5-10 × 10^5 cells in 25 µl DMEM:BME mixture (1:1) were used**.**

The *in vivo* imaging System (IVIS) was used for tumor growth surveillance. To this end, the abdominal area of the mice was shaved and 150 mg/kg D-Luciferin (Promega, E1605) injected intraperitoneally, and imaging performed under anesthesia with isoflurane (1–2 vol.% in oxygen).

For the *in vivo* treatment experiments of total 2 weeks, pemigatinib (Biosynth, #NKC85777) dissolved in 1% Tween-80 (Sigma-Aldrich, P1754) was administered at 3 mg/kg body weight per oral gavage once daily 5 out of 7 days for a total of 2 weeks to PFfPt tumor-bearing C57BL/6J mice.

Human CCA study population

For the histological analysis of human FGFR2 fusion-driven and other control iCCA, patients were identified from the Liver Cancer Center Heidelberg (LCCH) registry, where data on molecular alterations is typically available for patients undergoing palliative systemic therapy. Samples were included only from treatment-naïve patients and where enough FFPE block material containing tumor tissue was available for sectioning.

Written informed consent was obtained from all patients prospectively enrolled in the LCCH registry, which was approved by the Heidelberg University ethics committee (ethical permit number S-693/2019), complied with the provisions of the Good Clinical Practice guidelines and the Declaration of Helsinki and local laws and fulfilled the Regulation (EU) 2016/679 of the European Parliament and of the Council of 27 April 2016 on the protection of natural persons with regard to the processing of personal data (ID number DSAN854-A-OS/5).

Histopathology and immunostaining

Tissue specimens were fixed in 4% paraformaldehyde or 10% formalin solution over night at room temperature, washed with PBS and 70% ethanol, and

embedded in paraffin. Serial sections of 3-5 µm thickness were generated and rehydrated. Hematoxylin and eosin and periodic acid-Schiff (PAS) staining was performed following routine histological protocols. For immunohistochemical analyses, sections were treated with citric-acid based pH 6.0 Antigen Unmasking Solution (Biozol, H-3300) in a steamer for 20 min. Slides were then treated with 0.3% hydrogen peroxide to quench endogenous peroxidase activity. To prevent non-specific antibody binding, slides were incubated in protein blocking solution (5% donkey Serum [Biozol, LIN-END9010] in 1% BSA/PBS/0.1% Tween 20) for 1 hour before overnight incubation with primary antibody at 4°C (**Table S2**). After washing, appropriate biotinylated secondary antibodies (Vector Labs) were used with the Vectastain Elite ABC-HRP reagent (Vector Labs, PK-7100) and Diaminobenzidine-reaction kit (Vector Labs, SK-4100) for visualisation of bound antibodies. Harris modified hematoxylin (Carl Roth, T864.1) was used to counterstain nuclei. Finally, slides were washed, dehydrated, and mounted using Vectamount (Vector Labs, H-5700-60).

Stained sections were scanned on a Zeiss Axio Scan.Z1 slide scanner at the DKFZ Light Microscopy Facility. Tumors were assessed qualitatively by an expert liver pathologist in a blinded fashion.

For immunofluorescence stains, 2-3 µm thick FFPE serial sections were cut and deparaffinized and rehydrated. For human tissue sections, antigen retrieval was performed using the Epredia™ Dewax and HIER Buffer H (#TA999DHBH) and slides were boiled at 102°C for 1h. For murine tissue sections antigen retrieval was performed with EDTA based buffer (Leica, #AR9640) for 30min at 100°C or with a TRIS based buffer for 20 min in a steamer. Slides were washed and blocked with 5% donkey Serum (Biozol, LIN-END9010) in 1% BSA/PBS/0.1% Tween-20 for 30min. Afterwards cells were incubated with primary antibodies (**Table S2**) in blocking solution overnight at 4°C. After washing, slides were incubated with appropriate diluted fluorophore-conjugated secondary antibodies for 1h at RT, and then after washing, incubated with DAPI (1:100) for 5 min and mounted with of Prolong Gold antifade with DAPI.

Tyramide signal amplification (TSA) was performed using the Bond Polymer Refine Detection Kit (Leica: DS9800) and reagents FP1487001KT Opal 520 Reagent (FITC), FP1495001KT Opal 620 Reagent (Texas red), and FP1488001KT Opal 570 Reagent (Cy3) (Akoya) with antibodies and dilutions listed in **Table S2**. For PD-1 stainings a secondary antibody incubation was performed using a rabbit anti-goat antibody (Dako, P04990) before Opal reagents were added. Stained sections were scanned using Nano Zoomer S60 (Hamamatsu)

Tissue mounting and immunostaining for multiplexed ion beam imaging (MIBI)

Formalin-fixed and paraffin-embedded tissue blocks were sectioned at 5 µm and mounted onto gold-coated conductive slides (Ionpath, 567001). To minimize batch effects, all samples were processed together in parallel and stained with common antibody master mixes, following established protocols.[7] Briefly, sections were deparaffinized and rehydrated as for IHC preparation, then subjected to antigen retrieval. Non-specific binding was blocked for 1 h at room temperature, followed by M.O.M. reagent (Vector, MKB-2213-1) to prevent background from endogenous mouse IgG. Slides were then incubated overnight at 4 °C with a filtered primary antibody master mix for indirect staining. The following day, sections were washed and incubated with a filtered secondary antibody master mix for 1 h at room temperature. After washing, slides were incubated overnight at 4 °C with a filtered direct-staining antibody master mix. All antibodies used for MIBI staining are listed in **Table S3**. Following a final wash, sections were fixed in 2% (v/v) glutaraldehyde for 5 min at room temperature and dehydrated through a graded ethanol series. Prior to acquisition, stained slides were stored in a vacuum cabinet to prevent metal oxidation and preserve tissue integrity.

Multiplexed imaging and data preprocessing

H&E images of serial sections corresponding to the MIBI slides were used to identify tumor boundary regions for imaging. For each sample, one region was manually selected in DUEfiner, a customized interactive image viewer implemented in Python, and subdivided into 400 × 400 µm fields of view (FOVs).

Each FOV was acquired at a dwell time of 0.5 ms using the “Coarse” preset, yielding a pixel size of 390 nm.

Images were extracted from mass spectrometry data using Toffy (https://github.com/angelolab/toffy). For each pixel, the intensity of a given mass m was obtained by integrating ion counts over the range $[m - 0.3, m]$. To correct for systematic intensity gradients across FOVs — analogous to illumination correction in light and fluorescence microscopy —, all images were examined for such patterns. A gradient was defined as a consistent intensity variation present across channels and multiple FOVs. For each channel, the gradient was estimated by summing Gaussian-blurred versions of its FOVs, thereby reducing contributions from biological signals. Gradient magnitude scaled with channel mass, and a per-channel scaling factor was derived by linear regression between a proxy of gradient magnitude and channel mass. To further suppress biological signal, a two-dimensional correction map was generated by summing the scaled gradients across all channels. Correction was applied by dividing each channel by this map, adjusted for the channel-specific gradient magnitude across all FOVs.

Gradient-corrected images were subsequently processed with Rosetta compensation to reduce cross-channel contamination, as previously described.[8] The mass and relative abundance (coefficient) of interfering species in the source reagent are well characterized,[9] enabling estimation of their contribution by scaling the source channel intensity before subtracting it from the target channel. A coefficient matrix for all source–target channel pairs is available at https://doi.org/10.1101/2025.01.26.634557.

To remove gold-signal-based background, bare slide regions were identified and excluded based on elevated gold (Au) ion signal from the conductive slide coating. Pixel intensities in the Au channel were normalized to the 99.9th percentile and arcsinh-transformed (cofactor = 100). The transformed intensity distribution was modeled with a double-Gaussian fit to distinguish gold-negative and gold-positive populations. The intersection of the two Gaussian components was defined as the threshold for gold-positive pixels. This threshold was then back-transformed into raw intensity space and applied to each FOV to generate binary masks of gold-

positive pixels. These masks were subsequently used to exclude bare slide regions from downstream analysis.

Image-based single cell data quantification and filtering

FOV tiles were stitched into whole-region images. Cells were segmented in the stitched images using the Cellpose TN model,[10] re-trained on unpublished MIBI datasets comprising 335 cells from human normal liver, normal lung, colorectal cancer, and lung cancer tissues. The model was applied with a cell diameter of 30 pixels, a flow threshold of 0.7, and a cell probability threshold of –5. Cell masks were filtered based on size, nuclear intensity, and gold-pixel content to retain only high-confidence single cells. Masks smaller than 100 pixels were removed as over-segmented. For retained cells, features including spatial location in the whole-region image, cell size, and cell-size–normalized (mean) channel intensities were calculated. Histone H3 (HH3) intensities were either summed or averaged per cell, percentile-scaled, arcsinh-transformed, and thresholded using the intersection of a double-Gaussian fit to identify nuclei-negative populations. Cells below this threshold were flagged as out-of-plane. Similarly, cells with >50% of their area overlapping bare-slide pixels were flagged as incomplete. All flagged cells were excluded from downstream analysis.

Data annotation and tissue zone identification

To generate robust and accurate cell labels, we employed an iterative clustering strategy with human-in-the-loop curation across multiple levels, defined by the hierarchy of lineage markers (**Figure 4**). At each level, FlowSOM [11] was used to cluster cells into self-organizing map (SOM) nodes based on lineage markers relevant for that level. Nodes were then merged to align with defined cell lineages according to marker positivity and visual inspection. Nodes with ambiguous marker profiles were re-clustered and annotated; cells that remained ambiguous were labeled as “other.” Importantly, at each level, cells belonging to different higher-level lineages were classified separately to maintain lineage-specific resolution.

At level 1, cells were broadly classified into hepatocytes, cancer, endothelial, CAFs, and immune cells. At level 2, CAFs were further classified according to Vimentin (Vim), αSMA, COL1a1, and PDGFRb positivity, while immune cells were subdivided into $CD3^+$, $CD20^+$, $CD11c^+$, $CD11b^+$, and $CD11b^+CD11c^+$ populations. At level 3, $CD3^+$ cells were further divided into $CD4^+$, $CD8^+$, and $CD4^+FOXP3^+$ subsets, and $CD11b^+$ cells into $F4/80^{med}$, $Ly6G^+$, and $F4/80^{hi}$ subsets. At level 4, $F4/80^{hi}$ macrophages were split into $CD163^-$ and $CD163^+$ populations. Immune cells with ambiguous finer lineage profiles were marked as “other immune.” All annotation steps were performed using our custom viewer, UELer (https://github.com/HartmannLab/UELer).

PK tissues were divided into three zones: adjacent normal, tumor periphery, and tumor core. The tumor border was manually marked as the interface where hepatocytes confronted tumor cells, using tissue images with level 1 markers rendered in UELer. Relative to this border, the hepatocyte side was defined as adjacent normal, while the opposite side was defined as tumor. The tumor region was further subdivided into periphery and core based on distance from the border, using a cutoff of 150 µm.

MIBI statistical analysis

Cell densities were calculated as the counts of a specified cell type divided by the total area of all cells within a tumor. Cell densities between groups were compared via linear mixed-effects model with tumor type as a fixed effect and animal as a random intercept to account for multiple nodules per animal and a significance threshold of $p < 0.05$. Marker abundance between groups was compared via linear mixed-effects model with tumor type as a fixed effect, animal as a random intercept, and tumor nodules nested within animal as an additional random intercept to account for multiple cells per tumor per animal, with a significance threshold of $p < 0.05$. The proportion of Ki67-positive and Vimentin-positive cells within each cell population was determined by applying a predefined intensity threshold to classify individual cells as positive or negative, with per-tumor percentages calculated as the number of positive cells divided by the total cell

count. The positivity of Ki67 and Vimentin between groups was compared via binomial generalized linear mixed-effects model with group (PFfPt vs. PK) as a fixed effect and animal as a random intercept to account for multiple nodules per animal. All significance values were determined from permutation-based p-values corrected for multiple testing using the false discovery rate (FDR) method, applied across comparisons within each data type (i.e., cell density, Ki67 positivity, Vimentin positivity, and marker abundance separately).

Spatial Interaction Analysis

To characterize cell-type co-localization patterns in the tumor microenvironment, we performed spatial interaction analysis using the Conditional Z-score Interaction (COZI) method implemented within the SCIMAP framework (https://github.com/SchapiroLabor/scimap_COZI/tree/master).[12] Prior to analysis, hepatocytes, uninformative cell populations ("other immune cell," "other"), and in the PK samples, cells residing in the core zone were excluded. For each condition, spatial interactions were computed using a K-nearest-neighbor graph (K = 5) with 300 permutations per tumor nodule, applying conditional normalization and Z-score-based significance estimation. For each directed cell-type pair (source → target), Z-scores were summarized at the per-condition level by taking the median across tumor nodules, and permutation-based *p*-values were averaged accordingly. Cell-type pairs with a mean *p*-value ≥ 0.05 were considered non-significant and excluded from the interaction network.

FGFR inhibitor treatment in vitro

For the colony forming assay, PK and PFf organoids were first grown as a monolayer on the cell culture dish for several passages with media transition to DMEM with 10% FBS and 1% Penicillin/Streptomycin(P/S). Cells below passage 20 were used during the experiments and were seeded in 6-well plates at 500 cells/well for PK and 1000 cells/well for PFf with 2 ml of DMEM with 10% FBS. On the following day, cells were treated with the indicated concentrations of pemigatinib (MedChemExpress, #HY-109099), futibatinib (MedChemExpress,

#HY-100818) or vehicle (DMSO). Maximal DMSO concentration used was 560 nM. Media was renewed every 3 days. After 15 days for PK and 22 days for PFf, colonies were fixed with 70% ethanol for 10 minutes at room temperature and stained with 1% (w/v) crystal violet in distilled water for 10 minutes. Excess dye was removed by rinsing plates with tap water, followed by air-drying overnight. For quantification, crystal violet was extracted using 1 mL of 10% acetic acid (Thermo Fisher, #A38212) on a shaker for 1 hour at room temperature. The extracts were diluted 1:4 and absorbance was measured at 600 nm using a GloMax Explorer System (Promega). Absorbance from blank wells (10% acetic acid only) was subtracted.

For organoid growth inhibition, PK and PFf organoids were seeded as 2000 cells in 20 µl BME per well in white 96-well microplates. After BME solidification, 100 µl of organoid media without mEGF and hHGF was added. The next day cells were treated with the 400nM of pemigatinib (MedChemExpress, #HY-109099), 400nM futibatinib (MedChemExpress, #HY-100818) or vehicle (DMSO 0.00004 %). Media was renewed every 3rd day. Cell viability was assessed at the indicated time points using CellTiter-Glo® 3D Cell Viability Assay (Promega, #G9683), according to the manufacturer's instructions.

RNA sequencing and integrative data analysis

RNA was isolated using the RNeasy Plus Mini kit (Qiagen, 74134), and RNA integrity was analyzed by a 2100 Bioanalyzer using the RNA 600 Nano Kit (Agilent technologies) according to the manufacturer's protocol. Sequencing libraries were prepared by the Genomics and Proteomics Core Facility (DKFZ, Heidelberg, Germany) from total RNA using the Illumina TruSeq Stranded Total RNA Library Prep Kit according to the manufacturer's instructions. For sequencing, multiplexed libraries were sequenced in a paired-end setting (100 bp) on an Illumina NovaSeq 6000 sequencer.

Data was analyzed with the DKFZ Omics IT and Data Management Core Facility(ODCF) RNAseq workflow (version 4.0.0). Reads were aligned by 2-pass alignment to the mouse genome mm10 with PhiX decoy sequence and GENCODE

mouse vM12 annotation using the STAR aligner version 2.5.3a. The raw counts were obtained using the featureCounts tool from Subread (v1.6.5).[13]

Downstream analyses were performed in R 4.4.3. Differential expression analysis was conducted with DESeq2 v1.46.0.[14] Genes were retained if they had at least 10 counts in at least three samples. The DESeq2 model used the design formula `~ line * drug`, with PK and DMSO set as the reference levels. Genes with Benjamini-Hochberg false-discovery-rate adjusted *p* values < 0.05 and absolute log2 fold changes > 1 were considered differentially expressed. Heatmaps were generated from row-scaled, variance-stabilized expression values for the top protein-coding genes ranked by the absolute DESeq2 Wald statistic.

Gene set enrichment analysis was performed using genes ranked by the DESeq2 Wald statistic.[15] MSigDB mouse Hallmark gene sets,[16] were tested with the fgsea R package (v1.32.4) Click or tap here to enter text.GO Biological Process enrichment[17] was performed with clusterProfiler v4.14.6[18,19] using org.Mm.eg.db Ensembl annotations. Redundant GO terms were simplified separately for positively and negatively enriched terms using semantic similarity based on the Wang method.[20] Pathways with FDR-adjusted p values < 0.05 were considered significant, and normalized enrichment scores and adjusted p values were reported.

Statistical Analysis/Software

Data were analyzed and plotted using QuPath (V0.7), Living Image (IVIS) and GraphPad Prism 11 (GraphPad). For quantitative analyses of immunostains, the whole scanned images were loaded into QuPath.[21] Within QuPath, annotations were created to separate a tumor area from the surrounding liver tissue. Automated cell detection and classification tools were used to identify and quantify positive cells within the defined tumor area and were manually checked and corrected where necessary. Ki67 positivity was evaluated using high-power fields (HPFs). For each tissue section, 5–6 HPFs were randomly generated. Quantitative analysis was performed using QuPath software with trained object classifiers.

For the analysis of human tissue stains, random 10 HPFs of 1mm^2 were created using the automated region classifier.

Technical and biological replicates and respective statistical test used are specified for each experiment in the figure legends. Figure panels were generated by Affinity (Canva). For the creations of illustration panels in Figure 1A, 1C, 5A and 7A icons from BioRender.com were used.

Supplementary References

[1] Miyoshi H, Stappenbeck TS. In vitro expansion and genetic modification of gastrointestinal stem cells in spheroid culture. Nature Protocols 2013;8:2471–82. https://doi.org/10.1038/nprot.2013.153.

[2] **Pepe-Mooney BJ**, **Dill MT**, Alemany A, et al. Single-Cell Analysis of the Liver Epithelium Reveals Dynamic Heterogeneity and an Essential Role for YAP in Homeostasis and Regeneration. Cell Stem Cell 2019;25:23-38.e8. https://doi.org/10.1016/j.stem.2019.04.004.

[3] **Ran FA**, **Hsu PD**, Wright J, et al. Genome engineering using the CRISPR-Cas9 system. Nature Protocols 2013;8:2281–308. https://doi.org/10.1038/nprot.2013.143.

[4] **Platt RJ**, **Chen S**, Zhou Y, et al. CRISPR-Cas9 Knockin Mice for Genome Editing and Cancer Modeling. Cell 2014;159:440–55. https://doi.org/10.1016/j.cell.2014.09.014.

[5] Conant D, Hsiau T, Rossi N, et al. Inference of CRISPR Edits from Sanger Trace Data. The CRISPR Journal 2022;5:123–30. https://doi.org/10.1089/crispr.2021.0113.

[6] Ball M, Beck S, Wlochowitz D, et al. Diagnostic whole transcriptome sequencing in a series of 1233 FFPE solid tumor samples. Br J Cancer 2026;134:1101–10. https://doi.org/10.1038/s41416-025-03307-8.

[7] Bosse MM. MIBI staining v2 2022. https://doi.org/10.17504/protocols.io.dm6gprk2dvzp/v5.

[8] Vulliard L, Glauner T, Truxa S, et al. Robust multicellular programs dissect the complex tumor microenvironment and track disease progression in colorectal adenocarcinomas. 2025. arXiv:2510.05083. https://doi.org/10.48550/arXiv.2510.05083

[9] **Greenwald NF**, **Nederlof I**, Sowers C, et al. Temporal and spatial composition of the tumor microenvironment predicts response to immune checkpoint inhibition. bioRxiv 2025:2025.01.26.634557. https://doi.org/10.1101/2025.01.26.634557.

[10] Pachitariu M, Stringer C. Cellpose 2.0: how to train your own model. Nat Methods 2022;19:1634–41. https://doi.org/10.1038/s41592-022-01663-4.

[11] Gassen SV, Callebaut B, Helden MJV, et al. FlowSOM: Using self-organizing maps for visualization and interpretation of cytometry data. Cytometry Part A 2015;87:636–45. https://doi.org/10.1002/cyto.a.22625.

[12] Schiller C, Ibarra-Arellano MA, Bestak K, et al. Comparison and optimization of cellular neighbor preference methods for quantitative tissue analysis. Nat Commun 2026;17:3514. https://doi.org/10.1038/s41467-026-71699-z.

[13] Liao Y, Smyth GK, Shi W. featureCounts: an efficient general purpose program for assigning sequence reads to genomic features. Bioinformatics 2014;30:923–30. https://doi.org/10.1093/bioinformatics/btt656.

[14] Love MI, Huber W, Anders S. Moderated estimation of fold change and dispersion for RNA-seq data with DESeq2. Genome Biol 2014;15:550. https://doi.org/10.1186/s13059-014-0550-8.

[15] **Subramanian A**, **Tamayo P**, Mootha VK, et al. Gene set enrichment analysis: A knowledge-based approach for interpreting genome-wide expression profiles. Proc Natl Acad Sci 2005;102:15545–50. https://doi.org/10.1073/pnas.0506580102.

[16] Castanza AS, Recla JM, Eby D, et al. Extending support for mouse data in the Molecular Signatures Database (MSigDB). Nat Methods 2023;20:1619–20. https://doi.org/10.1038/s41592-023-02014-7.

[17] Aleksander SA, Balhoff J, Carbon S, et al. The Gene Ontology knowledgebase in 2023. GENETICS 2023;224:iyad031. https://doi.org/10.1093/genetics/iyad031.

[18] **Xu S**, **Hu E**, **Cai Y**, et al. Using clusterProfiler to characterize multiomics data. Nat Protoc 2024;19:3292–320. https://doi.org/10.1038/s41596-024-01020-z.

[19] Yu G, Wang L-G, Han Y, et al. clusterProfiler: an R Package for Comparing Biological Themes Among Gene Clusters. OMICS: A J Integr Biol 2012;16:284–7. https://doi.org/10.1089/omi.2011.0118.

[20] Wang JZ, Du Z, Payattakool R, et al. A new method to measure the semantic similarity of GO terms. Bioinformatics 2007;23:1274–81. https://doi.org/10.1093/bioinformatics/btm087.

[21] Bankhead P, Loughrey MB, Fernández JA, al. QuPath: Open source software for digital pathology image analysis. Sci Rep 2017;7:16878. https://doi.org/10.1038/s41598-017-17204-5.

[22] The Uniprot Consortium. UniProt: the Universal Protein Knowledgebase in 2025. Nucleic Acids Res 2024;53:D609–17. https://doi.org/10.1093/nar/gkae1010.

[23] **Sievers F**, **Wilm A**, Dineen D, et al. Fast, scalable generation of high-quality protein multiple sequence alignments using Clustal Omega. Mol Syst Biol 2011;7:MSB201175. https://doi.org/10.1038/msb.2011.75.

Fig. S1

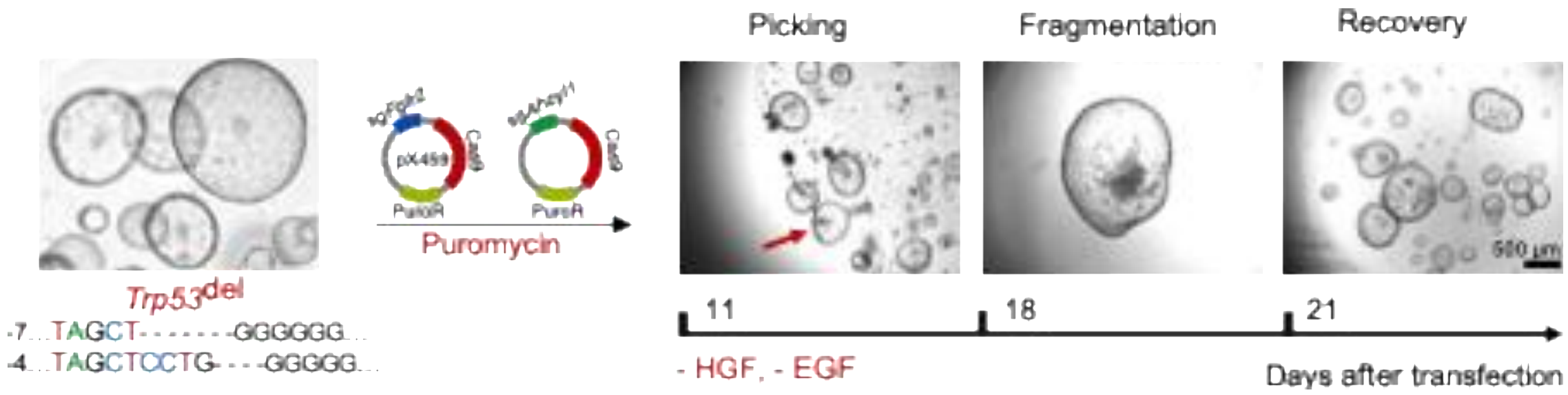

Fig. S1. Work flow of generating an *Fgfr2* fusion containing murine cholangiocyte organoid line.

Trp53 deleted cholangiocyte organoid line was transfected with 2 plasmids containing each an sgRNA targeting the respective intronic regions of *Fgfr2* and *Ahcyl1*. After transfection, functional selection with growth factor withdrawal allowed picking of single organoid clones which were then expanded and analysed for presence of *Fgfr2::Ahcyl1* fusion.

Fig. S2

A. AHCYL1

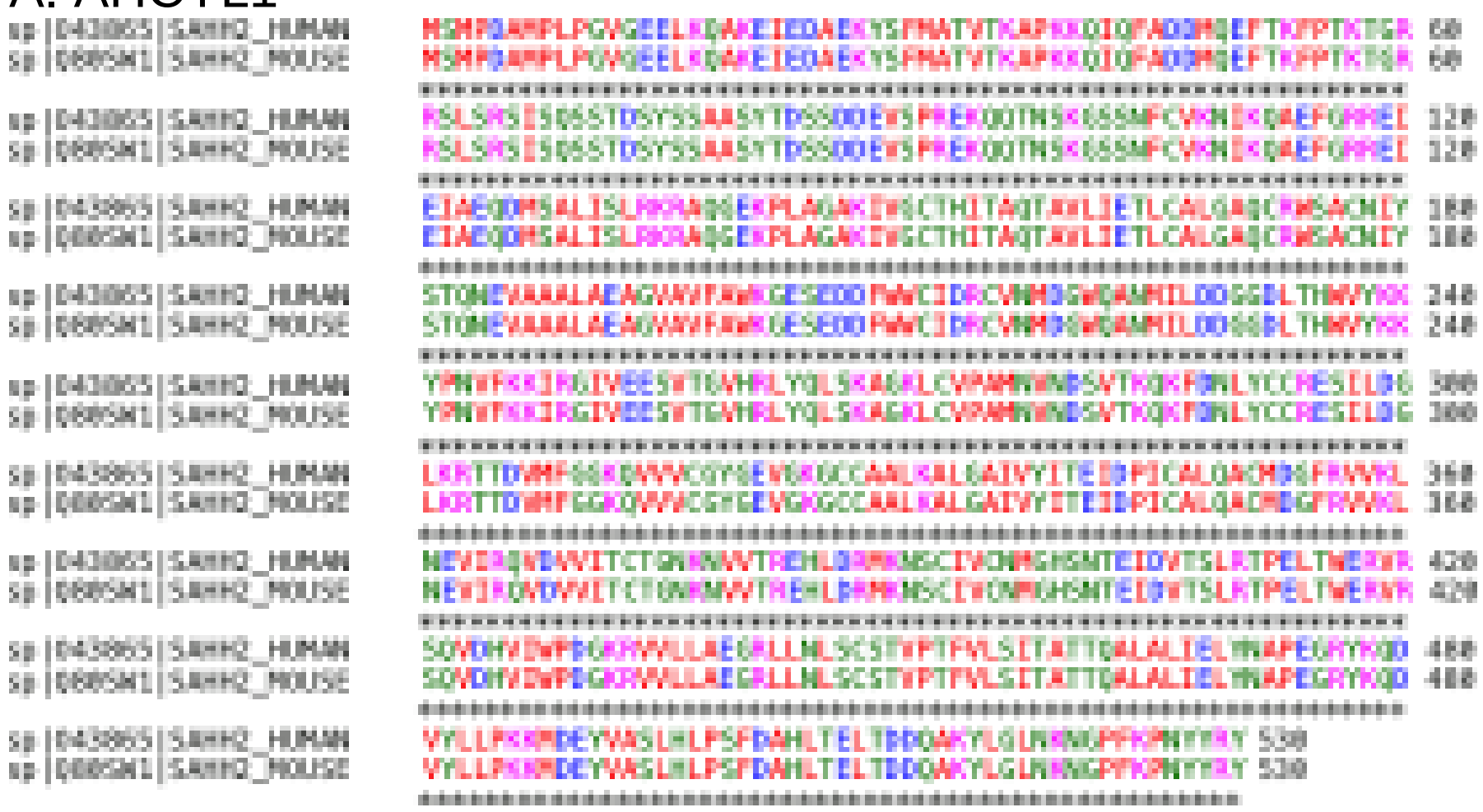

B. BICC1

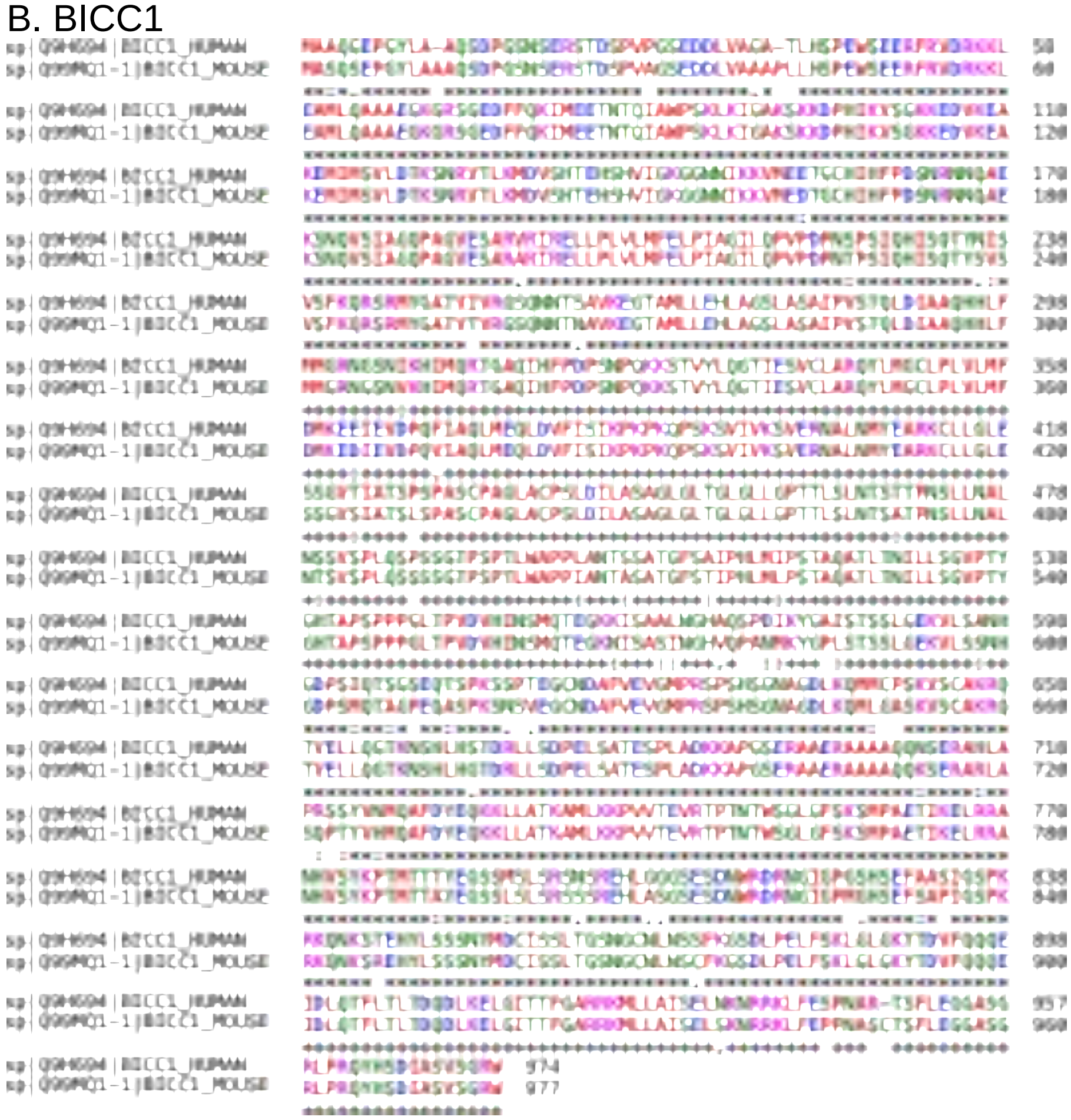

C. TACC3

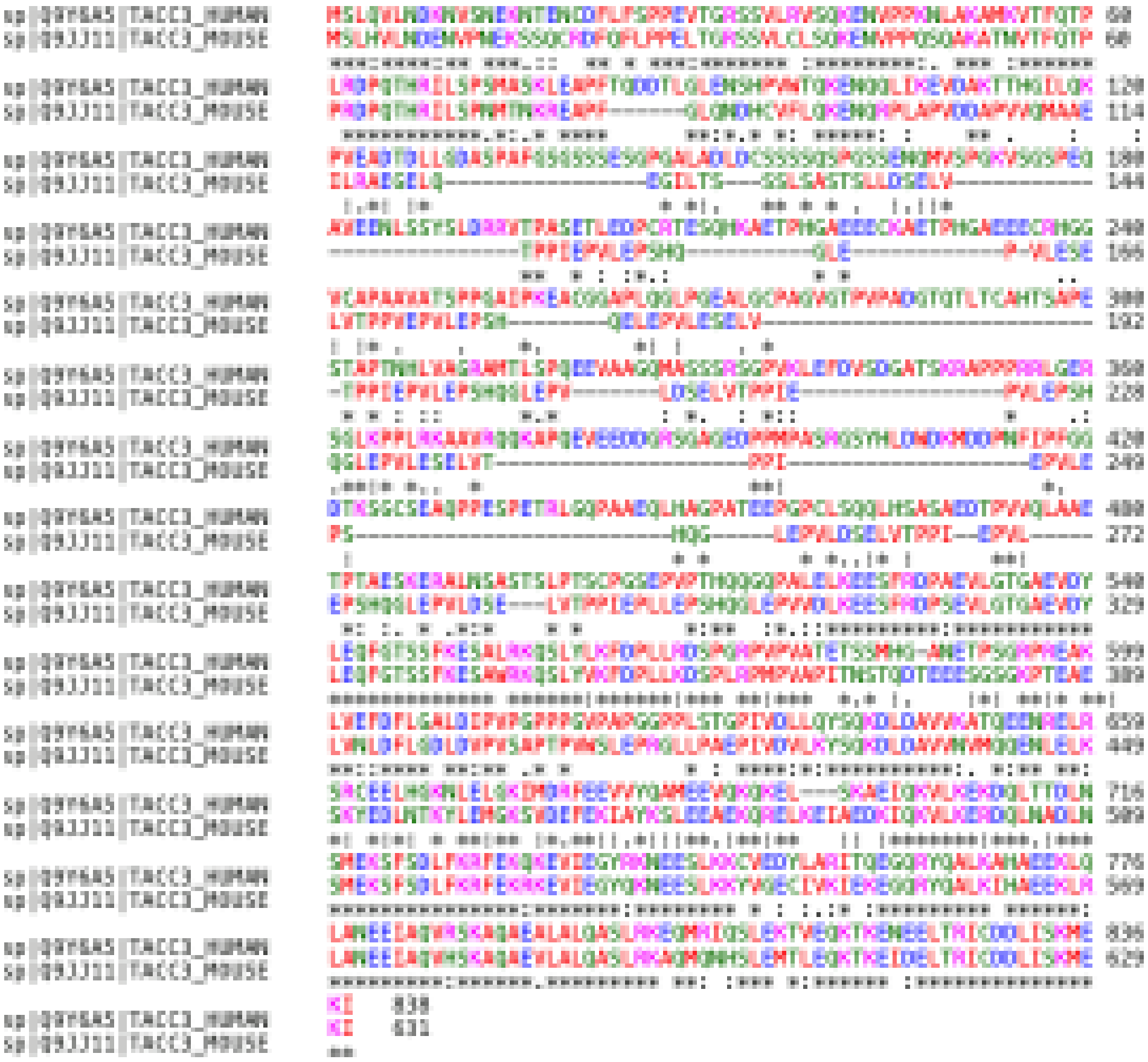

Fig. S2. Protein sequence alignments of the selected three FGFR2 fusion partners.

Amino acid sequences of the respective human and murine proteins **(A)** AHCYL1, **(B)** BICC1, and **(C)** TACC3 were extracted from the Uniprot data base[22] and aligned with Clustal Omega[23] .

Fig. S3

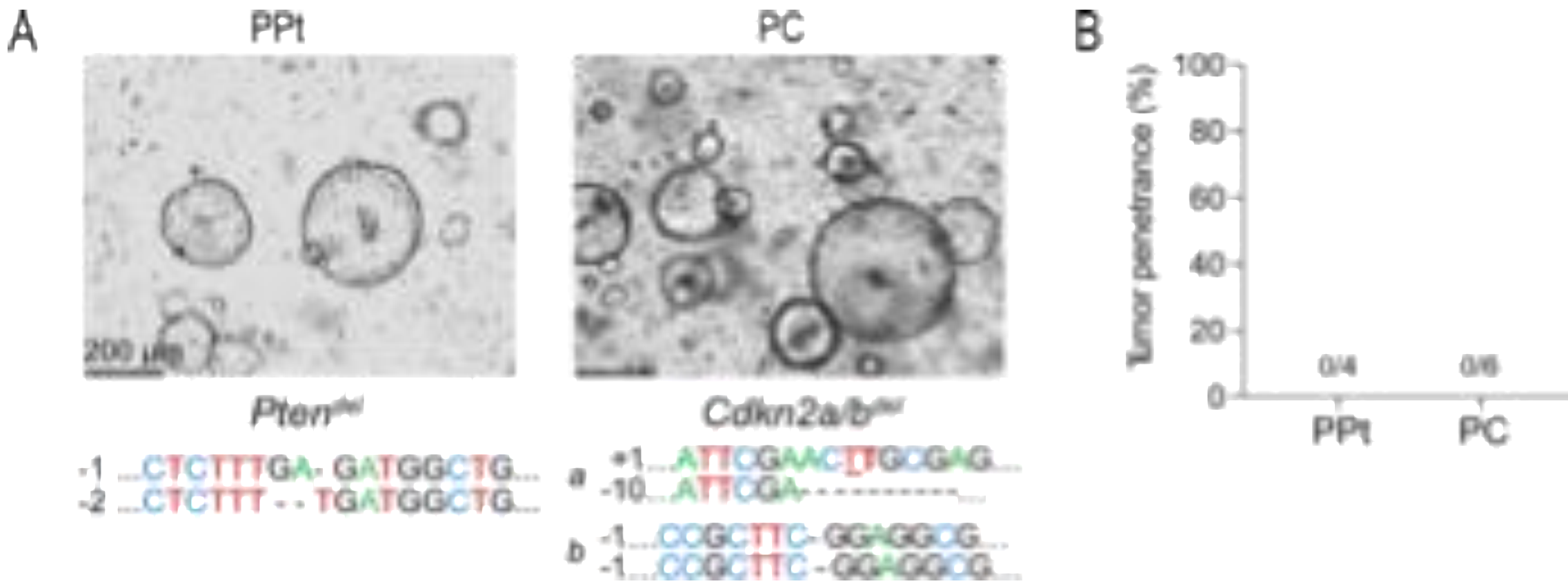

Fig. S3. Generation of PPt and PC control cholangiocyte organoid lines.
(A) Microscopic bright field images of cholangiocyte organoid lines harboring *Trp53* and *Pten* gene deletion (PPt) or *Trp53* and *Cdkn2a* and *Cdkn2b* gene deletion (PC) and respective genetic alterations specified below. Scale bar indicates 200 µm. **(B)** Bar plot indicating lack of intrahepatic tumor development upon implantation with the respective number of mice tested.

Fig. S4

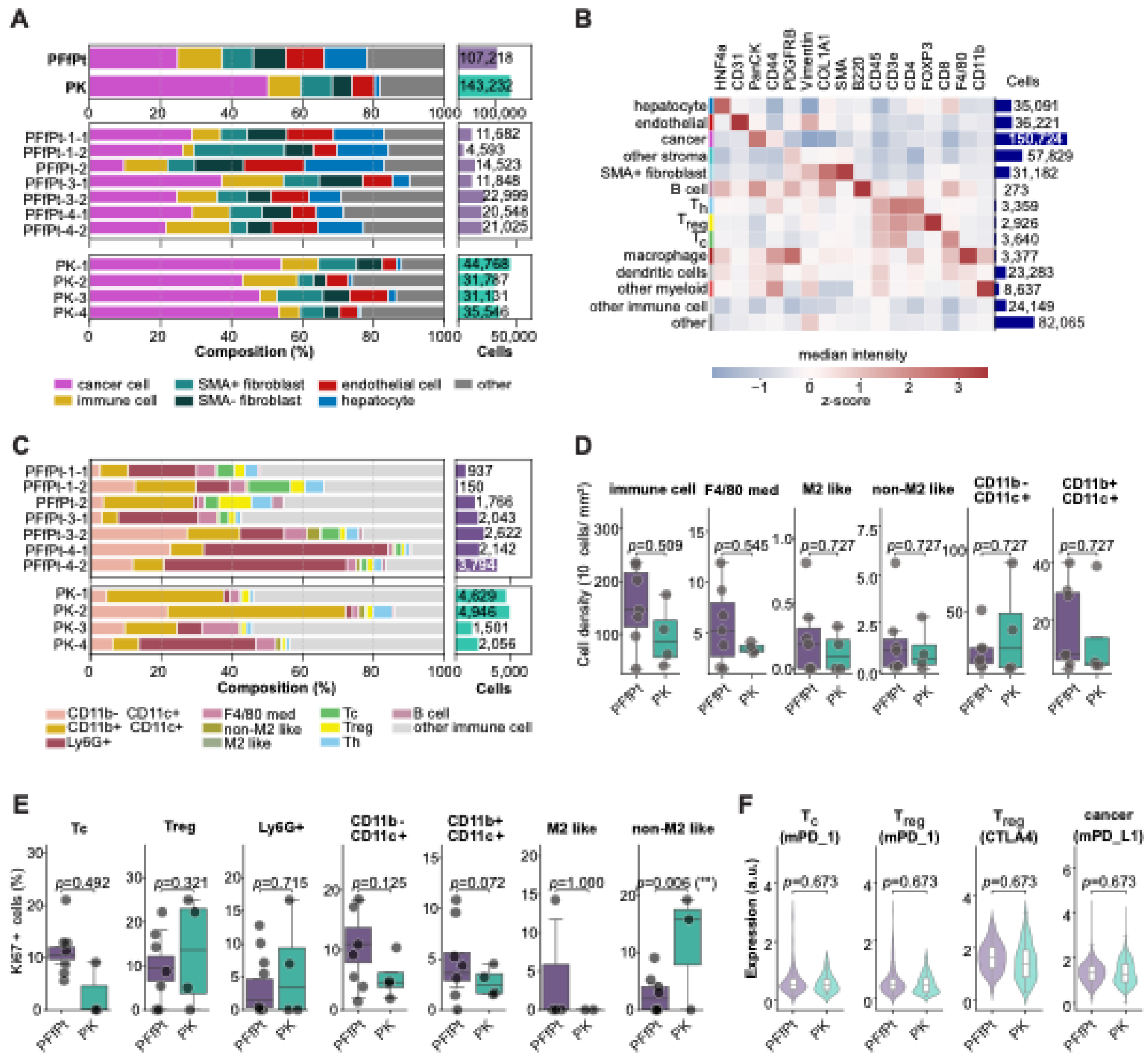

Fig. S4. In-depth analysis of TME cell populations via spatial proteomics.
(A) Stacked bar plots showing the broad cell lineage composition across samples. Total cell count per imaged tumor is indicated to the right. **(B)** Heatmap displaying the median marker intensity of lineage markers for each specified cell type. Total number of annotated cell type is indicated to the right. **(C)** Stacked bar plots showing the immune cell type composition across samples. Total immune cell count per imaged tumor is indicated to the right. Box plots depicting cell densities of indicated cell populations **(D)** and proportion of Ki67$^+$ cells per indicated cell type **(E)**. Box indicates interquartile range (IQR) and whiskers 1.5xIQR with bold line median; each data point indicates a tumor; exact p-values are indicated in the figure (binomial generalized linear mixed-effects model on 4 animal replicates each with a significance threshold of p <0.05). **(F)** Violin plots with integrated box plots depicting immune checkpoint expression intensities for the indicated cell types. No statistically significant differences between PFfPt and PK iCCA was observed.

Fig. S5

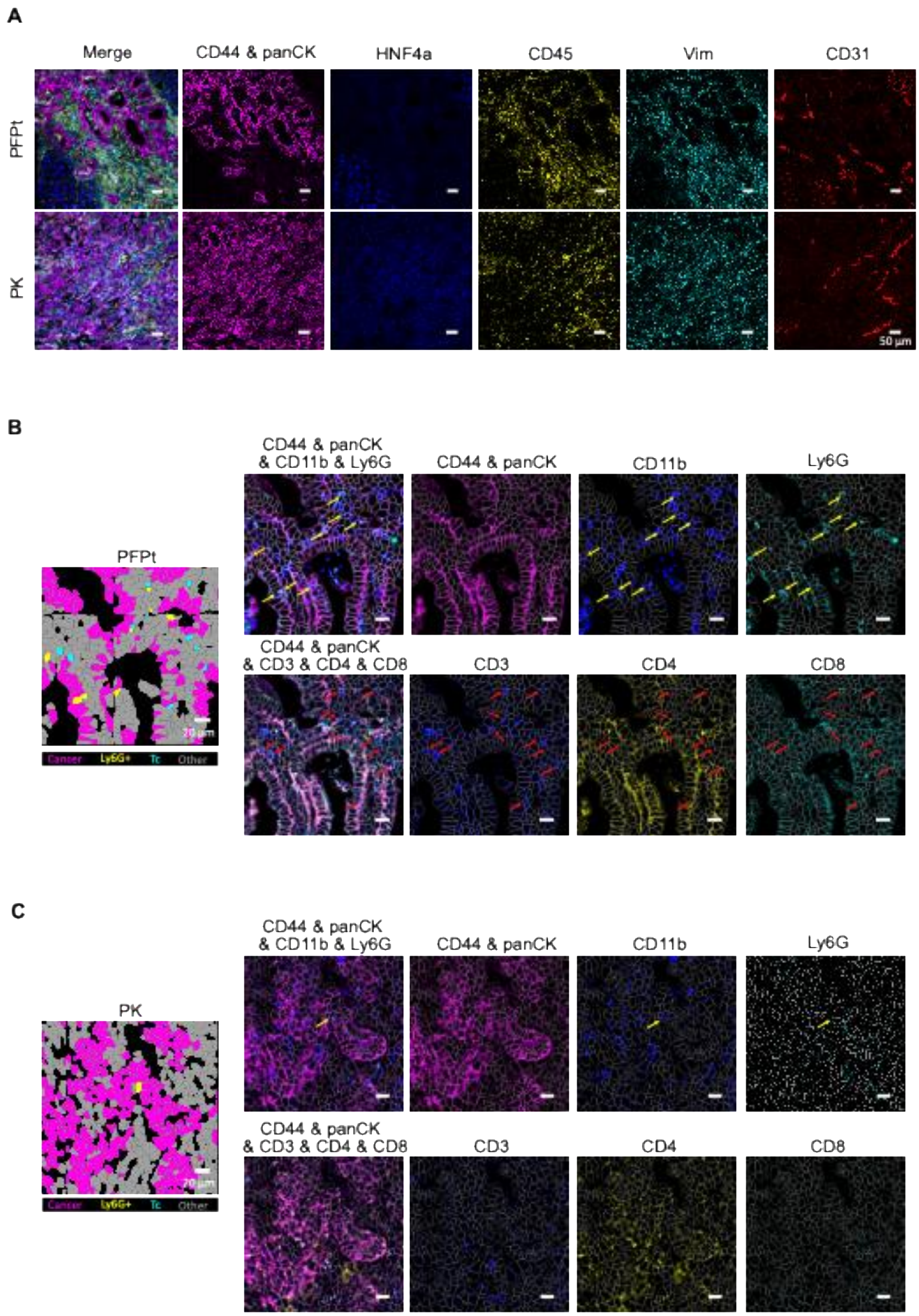
A
Merge
CD44 & panCK
HNF4a
CD45
Vim
CD31
PFPt
PK
50 µm
B
PFPt
CD44 & panCK & CD11b & Ly6G
CD44 & panCK
CD11b
Ly6G
CD44 & panCK & CD3 & CD4 & CD8
CD3
CD4
CD8
C
PK
CD44 & panCK & CD11b & Ly6G
CD44 & panCK
CD11b
Ly6G
CD44 & panCK & CD3 & CD4 & CD8
CD3
CD4
CD8

Fig. S5. Single-channel MIBI images to illustrate gating strategies.
(A) Single-channel MIBI images with indicated antibodies with the respective merged picture in **Figure 4C**. (**B-C**) Single-channel MIBI images with indicated antibodies to illustrate the gating strategy for the final cell annotation image to the left (**Figure 4H**). Red and yellow arrows highlight the cells in each channel considered positive in the annotation.
All scale bars indicate 20 µm.

Fig. S6

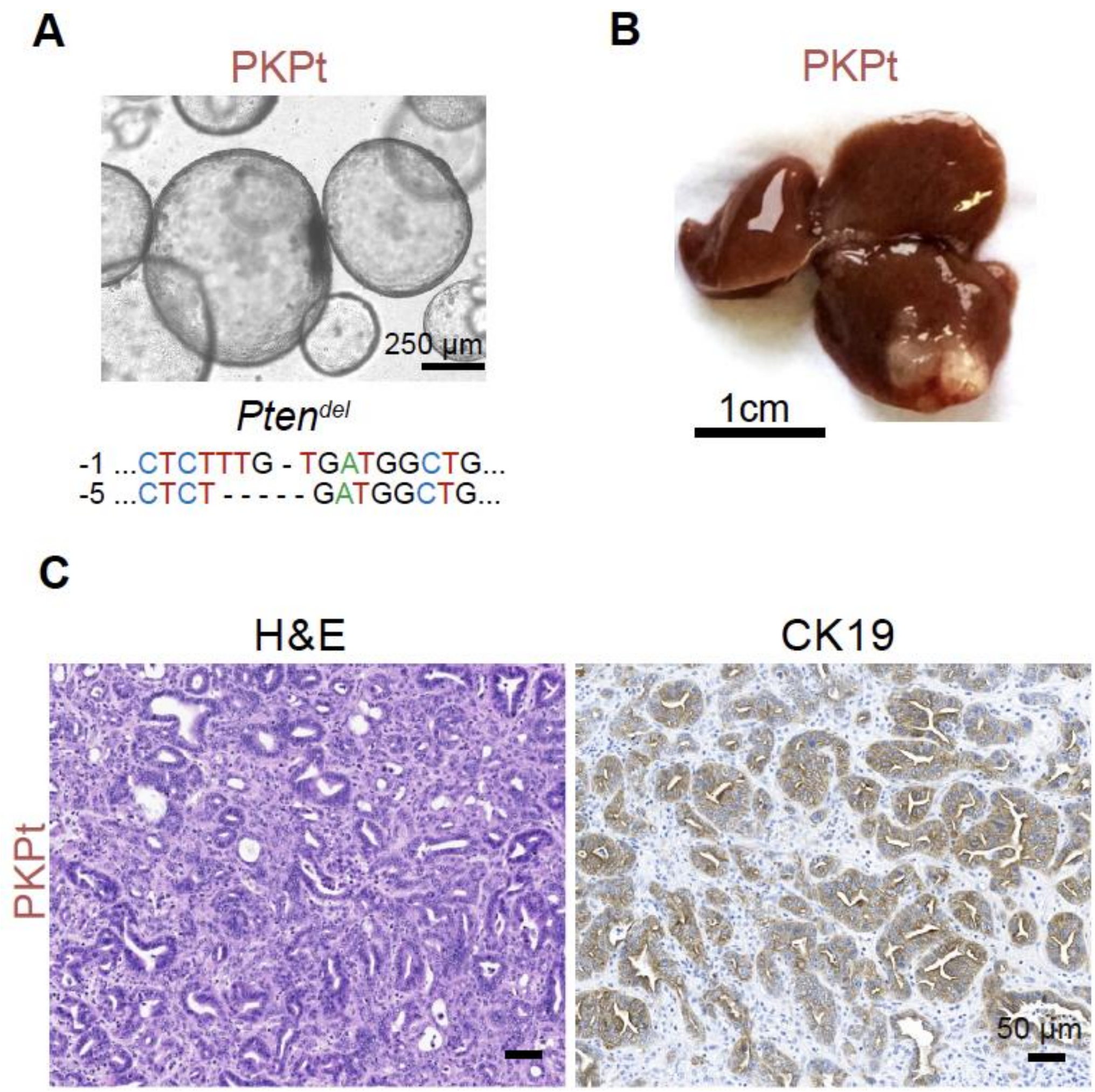

Fig. S6. Generation of PK line with *Pten* deletion.

(A) Microscopic bright-field image of PKPt CCA-organoid line and respective genetic alterations specified below. **(B)** Representative macroscopic image of PKPt iCCA. **(C)** Representative histologic pictures of PKPt iCCA for H&E and CK19 immunostain. Scale bars indicate 50 µm.

Fig. S7

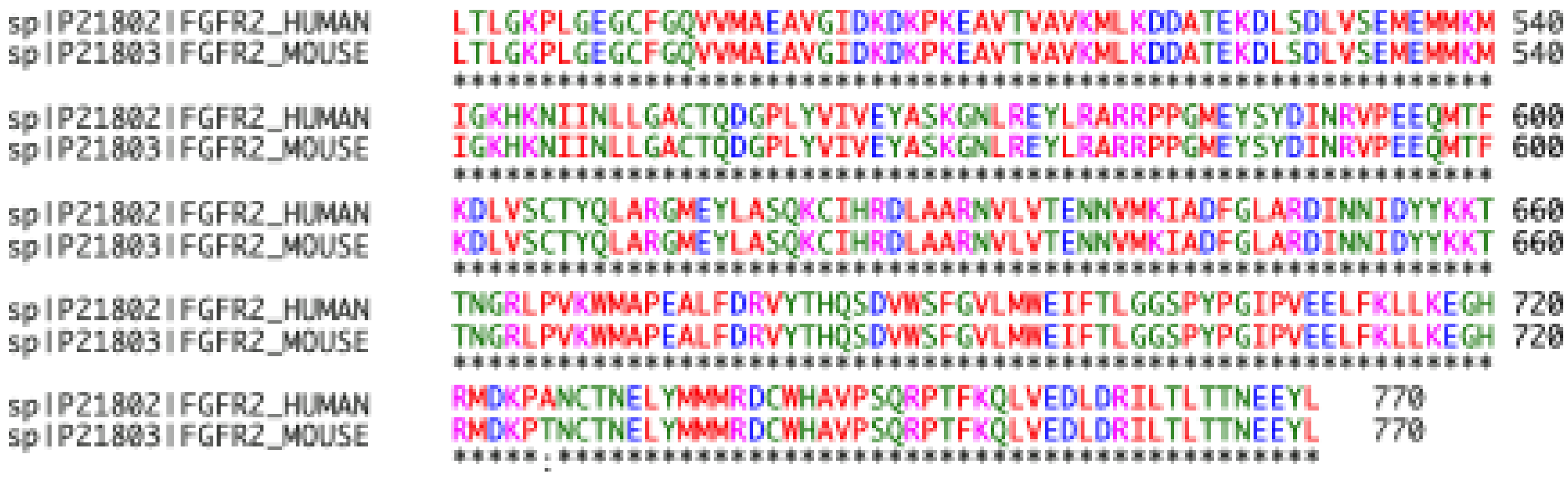

Fig. S7. Protein sequence alignments of the kinase domain of FGFR2.
Amino acid sequences of the respective human and murine kinase domain of FGFR2, which is > 99% conserved. Sequences were extracted from the Uniprot data base[22] and aligned with Clustal Omega[23].

Fig. S8

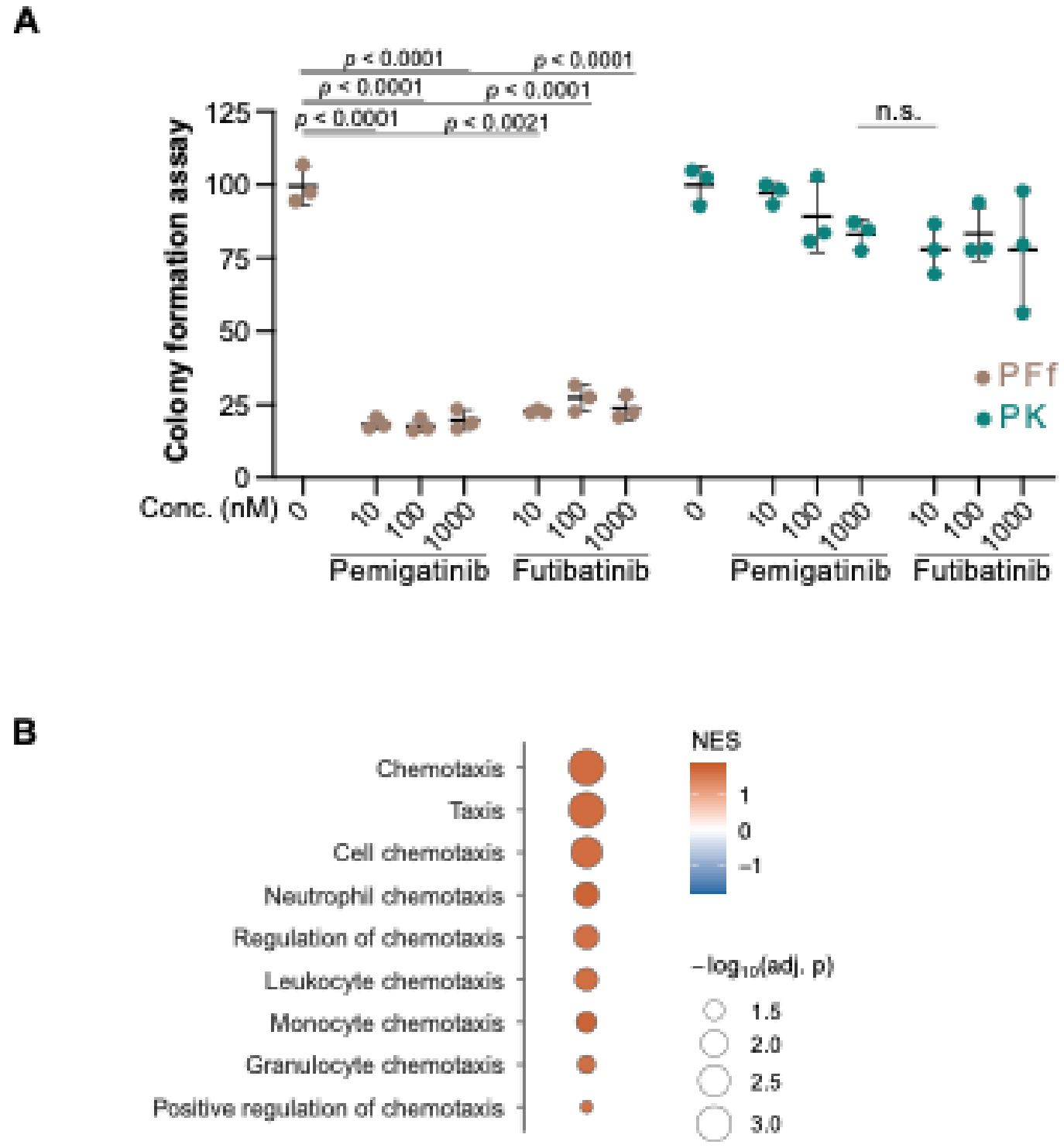

Fig. S8. FGFR inhibitor treatment *in vitro*.

(**A**) Scatter plot depicting results of colony formation of PFf and PK organoids (illustrated in **Figure 6**) treated with the indicated concentrations of pemigatinib and futibatinib normalized to DMSO control. N=3 per condition with line indicating mean±SD; exact p-values shown in figure with p≥0.05 considered not significant (One-way-ANOVA with Welch's correction). (**B**) GSEA of GO BP terms selected by semantic similarity to the chemotaxis term highlights enriched neutrophil and granulocyte migration signatures in PFf organoids.

Table S1. Lists of Oligonucleotides

sgRNAs

Targeted gene	sgRNA sequence (5´… 3´)	Source/design tool
Ahcyl1	TATTATAGGGTCTAAACAGT	Benchling [Biology Software] 2022
Bap1	GCTCTTCGATCCATTTGAAC	Artegiani et al. *Cell Stem Cell*, 2019; DOI: 10.1016/j.stem.2019.04.017
Bicc1	TGTTGCATACTCGTGATCCC	Benchling [Biology Software] 2022
Cdkn2a	ACGCCCTGAACCGCTTCGGG	Synthego CRISPR design tool
Cdkn2b	ACGCCCTGAACCGCTTCGGG	Synthego CRISPR design tool
Fgfr2	AAGTACTTACTAACTGACCC	Benchling [Biology Software] 2022
Pbrm1	GAAACCACTTCATAATAGTC	Synthego CRISPR design tool
Pten	GCTAACGATCTCTTTGATGA	Weber et al., *PNAS*, 2015 DOI: 10.1073/pnas.1512392112
Tacc3	ACAGGCACCAGTACCGACCT	Benchling [Biology Software] 2022
Trp53	GTGTAATAGCTCCTGCATGG	Platt et al., *Cell*, 2014 DOI: 10.1016/j.cell.2014.09.014

Primers for genomic PCR

Gene	Gene-ID	Primer Sequence (5´… 3´)
Ahcyl1	ENSMUSG00000027893	F: TGCAGTGTCACCCCAGCAGAGA<br>R: ACTTAGGCTAGGGGCTGGTGAGA
Bap1	ENSMUSG00000021901	F: CTGGTTTTGCAGCCGGAACCCT<br>R: AGCATCCTGGCCCGAAACAAGC
Bicc1	ENSMUSG00000014329	F: TGGCTATGTCGCAATTCTGGGTGT<br>R: TGGGTTCTGGGGCTTCGAACTCA
Cdkn2a	ENSMUSG00000044303	F: CTCCCGGCGATGTTCTACAGGA<br>R: AAAACTTTCCCCTGCGAAGGCC
Cdkn2b	ENSMUSG00000073802	F: ACGCTCACCGAAGCTACTGGGT<br>R: TTCTGTTGGGAGGGCTGTGCCT
Fgfr2	ENSMUSG00000030849	F: GTCTGCGCTTTTTGCCCCCTCT<br>R: CTGGCTGCTACCAAGGTCCCTT
Fgfr2-Ahcyl1 Fusion	-	F: GTCTGCGCTTTTTGCCCCCTCT<br>R: ACTTAGGCTAGGGGCTGGTGAGA
Fgfr2-Bicc1 Fusion	-	F: GTCTGCGCTTTTTGCCCCCTCT<br>R: AAGCTATGCTTGGGTCCGCAGC
Fgfr2-Tacc3 Fusion	-	F: GTCTGCGCTTTTTGCCCCCTCT<br>R: AGCTCCAAGTTCTCCTGCTGCA
Pbrm1	ENSMUSG00000042323	F: CACCAGCCCTGCCTTGCTCATT<br>R: CCACTGCCTGGCCCAAGAACTG
Pten	ENSMUSG00000013663	F: TCTTCTCCCCATTCCGCTGCCT<br>R: AAGGCTGGATTGACGAGCTCGC

Tacc3	ENSMUST00000079534	F: AGCCGACAGAAGCTGAGCTTGTCA<br>R: GAACAGCTGGTGGCCAAAACAGAT
Trp53	ENSMUSG00000059552	F: ATAGAGACGCTGAGTCCGGTTC<br>R: CCTAAGCCCAAGAGGAAACAGA

Primers for quantitative RT-PCR

Transcript	Transcript-ID	Primer Sequence (5´… 3´)
Ahcyl1	ENSMUST00000029490	F: CCAAGGAGATCGAGGACGCCGA<br>R: ACGAGACAAAGATCTCCGGCCA
Fgfr2	ENSMUST00000122054	F: GGCATGCTGTACCCTCACAG<br>R: GTGAGATCCAAGTATTCCTCATTGG
Fgfr2-Ahcyl1 Fusion	-	F: GACAAGCCCACCAACTGCACCA<br>R: ACGAGACAAAGATCTCCGGCCA
Gapdh	ENSMUST00000117757	F: CATCACTGCCACCCAGAAGACTG<br>R: ATGCCAGTGAGCTTCCCGTTCAG
Cxcl9	*ENSMUST00000113093.5*	*F: CCTAGTGATAAGGAATGCACGATG*<br>*R: CTAGGCAGGTTTGATCTCCGTTC*
Cxcl10	*ENSMUSG00000034855*	*F: ATCATCCCTGCGAGCCTATCCT*<br>*R: GACCTTTTTTGGCTAAACGCTTTC*
Cxcl1	*ENSMUSG00000029380*	*F: TCCAGAGCTTGAAGGTGTTGCC*<br>*R:AACCAAGGGAGCTTCAGGGTCA*
Cxcl2	*ENSMUST00000200681.3*	*F: CATCCAGAGCTTGAGTGTGACG*<br>*R: GGCTTCAGGGTCAAGGCAAACT*
Tnf	*ENSMUSG00000024401*	*F: GGTGCCTATGTCTCAGCCTCTT*<br>*R: GCCATAGAACTGATGAGAGGGAG*
Etv4	*ENSMUSG00000017724*	*F: CACAGACTTCGCCTACGACTCA*<br>*R: GCAGACATCATCTGGGAATGGTC*
Dusp6	*ENSMUSG00000019960*	*F: CTCGGATCACTGGAGCCAAAAC*<br>*R: TCTGCATGAGGTACGCCACTGT*
Rps14	*ENSMUSG00000024608*	*F: GACCAAGACCCCTGGACCT*<br>*R: CCCCTTTTCTTCGAGTGCTA*
Prf1	*ENSMUST00000035419.7*	*F: ACACAGTAGAGTGTCGCATGTAC*<br>*R: GTGGAGCTGTTAAAGTTGCGGG*

Table S2. List of antibodies

Antibodies	Source	Catalog number	RRID	Application
Anti-ACHYL1 Antibody	Proteintech	10658-3-AP	AB_2226013	1:1000,WB
Anti-mouse IgG, HRP-linked Antibody	Cell Signaling Technology	7076P2	AB_330924	1:3000, WB
Anti-rabbit IgG, HRP-linked Antibody	Cell Signaling Technology	7074P2	AB_2099233	1:3000, WB
CD8a Monoclonal Antibody (4SM15)	Thermo Fisher Scientific	14-0808-82	AB_2572861	1:100, IHC
CK19	Developmental Studies Hybridoma Bank	TROMA-III	AB_2133570	1:10, IHC
FGF receptor 2 (clone D4L2V)	Cell Signaling Technology	23328S	AB_2798862	1:500, WB
FOXP3 Monoclonal Antibody (FJK-16s)	Thermo Fisher Scientific	14-5773-82	AB_467576	1:100, IHC
Ly-6G/Ly-6C Monoclonal Antibody (RB6-8C5),	Thermo Fisher	14-5931-85	AB_467731	1:100, IHC
Rabbit Anti-Rat IgG Antibody (H+L), Biotinylated	Vector Laboratories	BA-4000	AB_2336206	1:1000, IHC
Vinculin Antibody (7F9) HRP	Santa Cruz Biotechnology	sc-73614 HRP	AB_2941767	1:1000, WB
β-Actin Antibody (C4) HRP	Santa Cruz Biotechnology	sc-47778 HRP	AB_2714189	1:5000, WB
Gmzb anti mouse monoclonal E5V2L	Cell signaling	44153	AB_2857976	1:200 IF
PD1 anti mouse Polyclonal	R&D	AF1021	AB_354541	1:100 IF
Anti mouse CD8a monoclonal D4W2Z	Cell signaling	98941S	AB_2756376	1:400 IF
Rabbit anti goat	Dako	P04490	-	1:300, IF
