# Supplementary material for: FGFR2 fusion-driven cholangiocarcinoma is characterized by a distinct neutrophil-enriched tumor microenvironment in a syngeneic murine model
Source: JHEP Rep. 2026 Jul 21;8(10):101964. doi: 10.1016/j.jhepr.2026.101964 (PMC13598188; doi:10.1016/j.jhepr.2026.101964)
Supplement: Supplementary file 3 [file mmc7.pdf]

FGFR2 fusion-driven cholangiocarcinoma is characterized by a distinct neutrophil-enriched tumor microenvironment in a syngeneic murine model

Authors

Nugzar Lekiashvili, Trinh Kieu Dinh, Alexander Olkus, …, Felix J. Hartmann, Thomas Longerich, Michael T. Dill

Correspondence

michael.dill@med.uni-heidelberg.de (M.T. Dill).

Graphical abstract

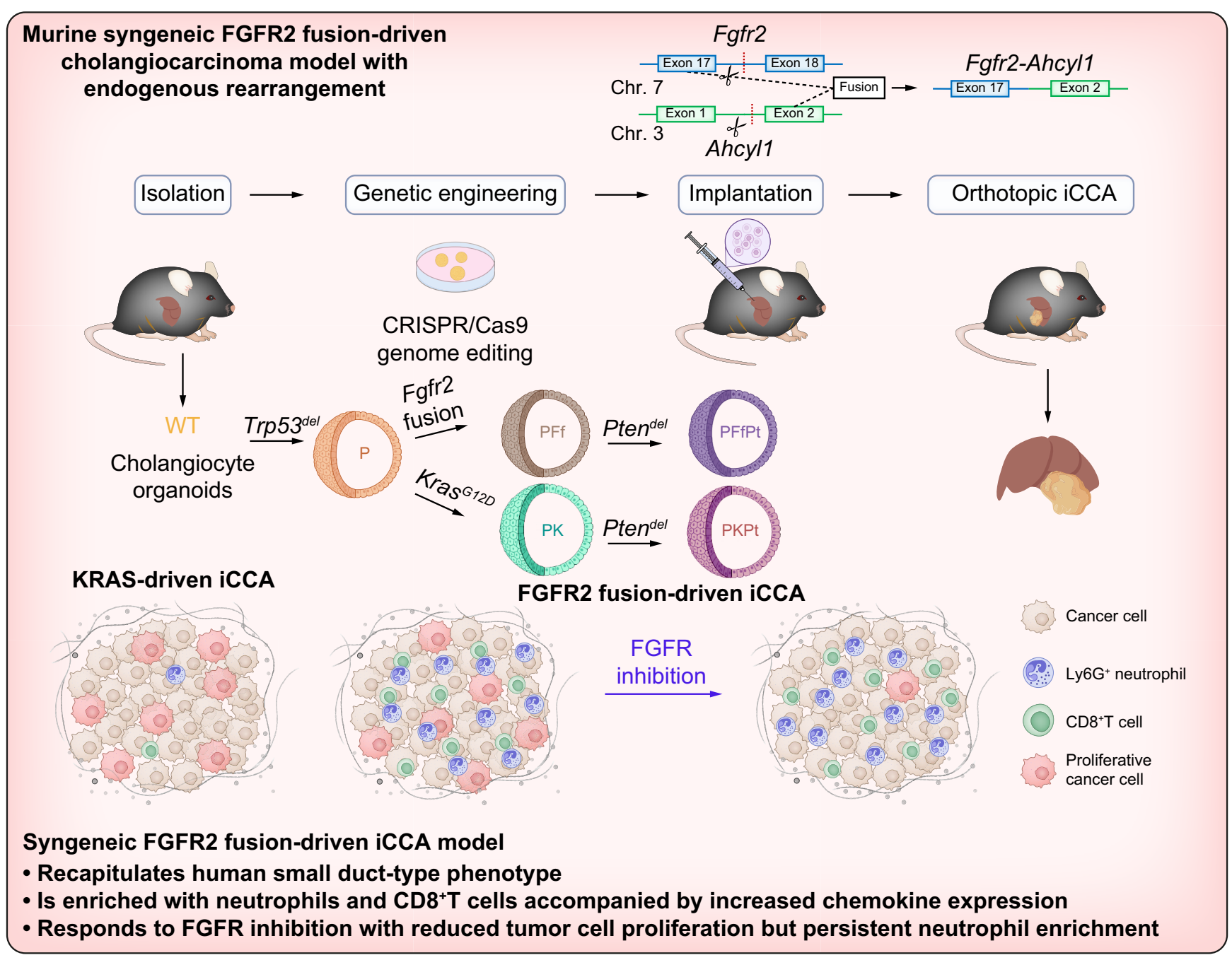

Highlights:

- First syngeneic murine iCCA model based on endogenous *Fgfr2* chromosomal rearrangement.
- Recapitulates small duct type morphology of human FGFR2 fusion-driven iCCA.
- FGFR2 fusion-driven iCCA shows distinct neutrophil and CD8+ T-cell enrichment.
- Chemotaxis gene upregulation in FGFR2 fusion-driven iCCA is not reversed by FGFR inhibition.

Impact and implications:

This study introduces the first fully syngeneic, endogenously engineered murine model of FGFR2 fusion-driven iCCA, overcoming the key limitation of prior models relying on human transgenes in immunodeficient hosts. The model faithfully recapitulates hallmarks of human FGFR2 fusion-driven iCCA, including a small duct type morphology and a distinct neutrophil-enriched tumor immune microenvironment, validating its translational relevance. The finding that an upregulated chemotaxis signature in FGFR2 fusion persists despite FGFR inhibition, alongside upregulation of interferon-stimulated genes upon treatment, points to compensatory immunomodulatory mechanisms that remain mechanistically unresolved. Overall, this work provides a physiologically relevant platform to interrogate cancer cell–tumor microenvironment crosstalk in FGFR2 fusion-driven iCCA.

https://doi.org/10.1016/j.jhepr.2026.101964
© 2026 The Authors. Published by Elsevier B.V. on behalf of European Association for the Study of the Liver (EASL). This is an open access article under the CC BY license (http://creativecommons.org/licenses/by/4.0/).

JHEP|Reports

FGFR2 fusion-driven cholangiocarcinoma is characterized by a distinct neutrophil-enriched tumor microenvironment in a syngeneic murine model

Nugzar Lekiashvili[1,2,†], **Trinh Kieu Dinh**[1,3,†], **Alexander Olkus**[1,2,4,5,†], **Yu-Le Wu**[6,†], **Chun-Shan Liu**[1,†], Daria Komkova[1,3,7], Albrecht Stenzinger[8], Felix J. Hartmann[6,9], Thomas Longerich[5,8], Michael T. Dill[1,4,5,*]

JHEP Reports **2026**. vol. 8 | 1–14

Check for updates

Background & Aims: Fibroblast growth factor receptor 2 gene (*FGFR2*) rearrangements are among the most common oncogenic drivers in intrahepatic cholangiocarcinoma (iCCA). Although FGFR inhibitors are clinically approved, primary and secondary resistance remain major limitations. Preclinical investigation of resistance mechanisms, including cancer cell-extrinsic crosstalk, is hampered by current models that rely on human *FGFR2* fusion transgenes in immunodeficient hosts. We therefore aimed at generating an entirely murine FGFR2 fusion-driven iCCA (Ff-iCCA) model to study immunomodulatory mechanisms in the tumor microenvironment (TME).

Methods: A syngeneic cholangiocyte organoid-based iCCA mouse model was engineered via endogenous chromosomal rearrangement of the *Fgfr2* gene combined with *Trp53* deletion (PFf) and other co-occurring genetic alterations. $Kras^{G12D}$-mutated lines (PK) served as comparison. TME characterization was performed using 30-plex spatial proteomics on ∼250,000 cells. Bulk RNA sequencing was conducted on FGFR inhibitor-treated PFf and PK organoids. Pharmacodynamics of FGFR inhibition on Ff-iCCA were assessed by immunostaining and quantitative RT-PCR.

Results: Intrahepatic implantation produced well-differentiated Ff-iCCA with morphologic features resembling human small duct type iCCA. In immunocompetent hosts, additional co-alterations were required for tumor penetrance, with *Pten* deletion being most robust with 75%. Compared with KRAS-driven iCCA, Ff-iCCA showed a significantly increased infiltration by Ly-6C/G+ neutrophils (19-fold, p = 0.004) and CD8+ T cells (eightfold, p <0.001). Transcriptome analysis revealed increased chemokine expression in PFf *vs*. PK organoids, which was not reversed by FGFR inhibition. Ff-iCCA responded to FGFR inhibition with a 6.5-fold reduced proliferation *in vivo* (p = 0.029), without observation of significant TME remodeling.

Conclusions: Our syngeneic murine Ff-iCCA model recapitulates hallmarks of human Ff-iCCA, providing a platform for functional investigation of cancer cell–TME crosstalk in this molecular subtype.

© 2026 The Authors. Published by Elsevier B.V. on behalf of European Association for the Study of the Liver (EASL). This is an open access article under the CC BY license (http://creativecommons.org/licenses/by/4.0/).

Introduction

Intrahepatic cholangiocarcinoma (iCCA) is a primary malignancy of the liver, with a rising global incidence and mortality.[1] The majority of patients are not eligible for curative resection at diagnosis and will eventually require palliative systemic therapy with a very poor prognosis and a 5-year-survival rate of 7–20%.[2] Over the past decade, intense tumor genome sequencing efforts have strongly increased our understanding of the genomic landscape in iCCA, and the entity has become exemplary for routine molecular diagnostics and targeted therapy.[2,3] Chromosomal rearrangement of the intronic region between exon 17 and 18 of the fibroblast growth factor receptor 2 (*FGFR2*) gene is among the most prevalent alterations in iCCA occurring with a frequency of 9–18%.[3–5] *FGFR2* can rearrange with dozens of diverse fusion gene partners that lead to a constitutive activation of FGFR2 signaling.[4,6,7] Consequently, the fusion partner enables ligand-independent receptor dimerization, leading to constitutive autophosphorylation of FGFR2 chains and the activation of downstream signaling pathways, such as RAS-MAPK, JAK-STAT, and PI3K-AKT-mTOR.[8] This oncogenic activation of FGFR2 signaling not only has cell-autonomous effects, including enhanced proliferation and invasion, but also immunomodulating and neoangiogenic effects.[8,9] Importantly, as iCCA comprises two histomorphologically distinct subtypes, defined as large duct and small duct type iCCA, *FGFR2* fusions occur almost exclusively in the small duct type.[10] This raises the question of whether the cell of origin underlying small duct type iCCA exerts a selective pressure favoring *FGFR2* rearrangement during malignant transformation, or

* Corresponding author. Address: German Cancer Research Center (DKFZ), Im Neuenheimer Feld 280, 69120 Heidelberg, Germany. Tel.: +49 6221 56 34064.
E-mail address: michael.dill@med.uni-heidelberg.de (M.T. Dill).
† These authors contributed equally to this work.

https://doi.org/10.1016/j.jhepr.2026.101964

whether FGFR2 pathway activation itself drives this distinct morphological phenotype.

The high prevalence of *FGFR2* fusion in iCCA has led to the successful clinical testing of FGFR inhibitors, which are now approved by the U.S. FDA and the European Medicines Agency for second-line palliative therapy. These inhibitors have shown effectiveness with objective response rates of 38% (pemigatinib) and 42% (futibatinib).[11,12] However, these data imply that primary resistance mechanisms occur in a considerable proportion of patients; secondary resistance also remains a relevant challenge, with sustained disease control >12 months being very rare.[13] *FGFR2* fusion commonly co-occurs in up to 63% with alterations in other genes, such as *TP53*, *BAP1*, *CDKN2A* and *CDKN2B*, *PBRM1*, and *PTEN*.[6,12] A statistically significant association with reduced objective response and progression-free survival has only been described for *TP53* in the FIGHT-202 trial, but overall case numbers for these subtypes were low.[6,12] Taken together, these clinical data underscore the need to improve inhibitor treatment and reduce resistance development, for example, by identifying rational synergistic therapy approaches.

Multiple oncogenic signaling pathways reportedly influence the tumor microenvironment (TME) and thus evade immunosurveillance.[14] To what extent FGFR2 signaling affects TME in iCCA remains systematically unexplored. Preclinical models are crucial to functionally assess mechanisms within the complex intercellular interplay of TME and could help inform rational treatment combination choices to be investigated clinically. Apart from patient-derived xenografts,[15–17] three different murine models of engineered FGFR2 fusion-driven iCCA (Ff-iCCA) have been described till date.[18–20] All models rely on transgenic introduction of a human Ff transcript, and successful tumor growth has only been demonstrated in immunodeficient mouse hosts, therefore limiting the potential to investigate immunomodulatory mechanisms.

Here, we report a novel Ff-iCCA model engineered with an endogenous, murine genome-based *Fgfr2* rearrangement. This model reveals immune cell infiltration patterns driven by FGFR2 activation and offers a physiologically relevant platform to mechanistically interrogate cancer cell–TME crosstalk.

Materials and methods

Organoid isolation and intrahepatic implantation

Wildtype cholangiocytes were isolated from C57BL/6J mice aged 4–6 weeks, adapting a published protocol,[21] and cultured as organoids. Genetically engineered organoid lines were then injected intrahepatically into mice via midline laparotomy under general anesthesia. All experiments were conducted in accordance with the German Animal Protection Law and approved by the Regierungspräsidium Karlsruhe (approval no. G34/22).

Human CCA study population

Suitable iCCA tissue samples with available genetic information were identified from the Liver Cancer Center Heidelberg (LCCH) registry. Written informed consent was obtained from all patients prospectively enrolled in the LCCH registry, which was approved by the Heidelberg University ethics committee (ethical permit number S-693/2019).

Comprehensive descriptions of all materials and methods are presented in the Supplementary Material.

Results

Generation of endogenous FGFR2 fusion in murine cholangiocyte organoids

To create endogenous murine *Fgfr2* fusion, we selected three common fusion partners in human Ff-iCCA (*Ahcyl1*, *Bicc1*, and *Tacc3*),[6] and targeted the respective introns to recreate rearranged exon-exon junctions commonly occurring in humans and allowing for ligand-independent FGFR2 activation (Fig. 1A,B). Wildtype cholangiocyte organoids from C57Bl/6J mice were generated to sequentially introduce alterations able to transform them into CCA organoids (Fig. 1C). The parental organoid line harboring *Trp53* deletion (P) was used to generate a monoclonal line with *Fgfr2* chromosomal rearrangement leading to a FGFR2 fusion protein upon functional selection through withdrawal of growth factors, which was interestingly only successful with the AHCYL1 fusion partner (Fig. S1). Of the three selected fusion partners, the murine AHCYL1 protein sequence is the most highly conserved in comparison to humans with 100% homology this may explain why this fusion was particularly successful. (Fig. S2). Accordingly, the rearrangement with the expected fused sequences was confirmed on both genomic DNA and mRNA level (Fig. 1D). Detection of the FGFR2 and AHCYL1 proteins by Western immunoblotting revealed the expected shifts in size, further confirming the presence of the gene fusion (Fig. 1E).

To reflect the molecular heterogeneity of human Ff-iCCA, alterations in eight different genes were induced including *Trp53* deletion (P), *Fgfr2-Ahcyl1* fusion (Ff), *Kras*^{G12D} (K), *Bap1* deletion (B), *Pbrm1* deletion (P), *Pten* deletion (Pt), and combined *Cdkn2a/b* deletion (C), thereby creating six different CCA organoid lines: PK, PFf, PFfPt, PFfC, PFfB, and PFfPb (Fig. 1C,F). The selected genes were chosen based on the most common co-occurring mutations in human Ff-iCCA specimens.[5,6] These lines were morphologically indistinguishable *in vitro* and maintained a spheric monolayer growth pattern as observed in their wildtype parental line. However, upon *Kras*^{G12D} mutation (line PK) or FGFR2 fusion (PFf), the cell lines continued to proliferate at a high rate despite the withdrawal of growth factors. This differed substantially from the growth rate of the cholangiocyte organoid line harboring only *Trp53* deletion, indicating the expected growth factor-independent activation of pro-proliferative pathways (Fig. 1G). In summary, we successfully created a range of cholangiocyte-derived murine CCA organoid lines based on an endogenous *Fgfr2* gene fusion.

Murine FGFR2 fusion-driven organoids develop small duct type iCCA in syngeneic hosts

We next systematically assessed the tumorigenic potential of each organoid line upon standardized intrahepatic implantation into the left liver lobe in syngeneic C57Bl/6J mice. The mice injected with the PK line showed rapid tumor formation (PK iCCA) within 4–10 weeks at a 100% penetrance. Conversely, the PFf line failed to induce liver tumors in this model by its own (zero of 11); of all the PFf lines harboring additional co-alterations, only PFfPt with *Pten* deletion and

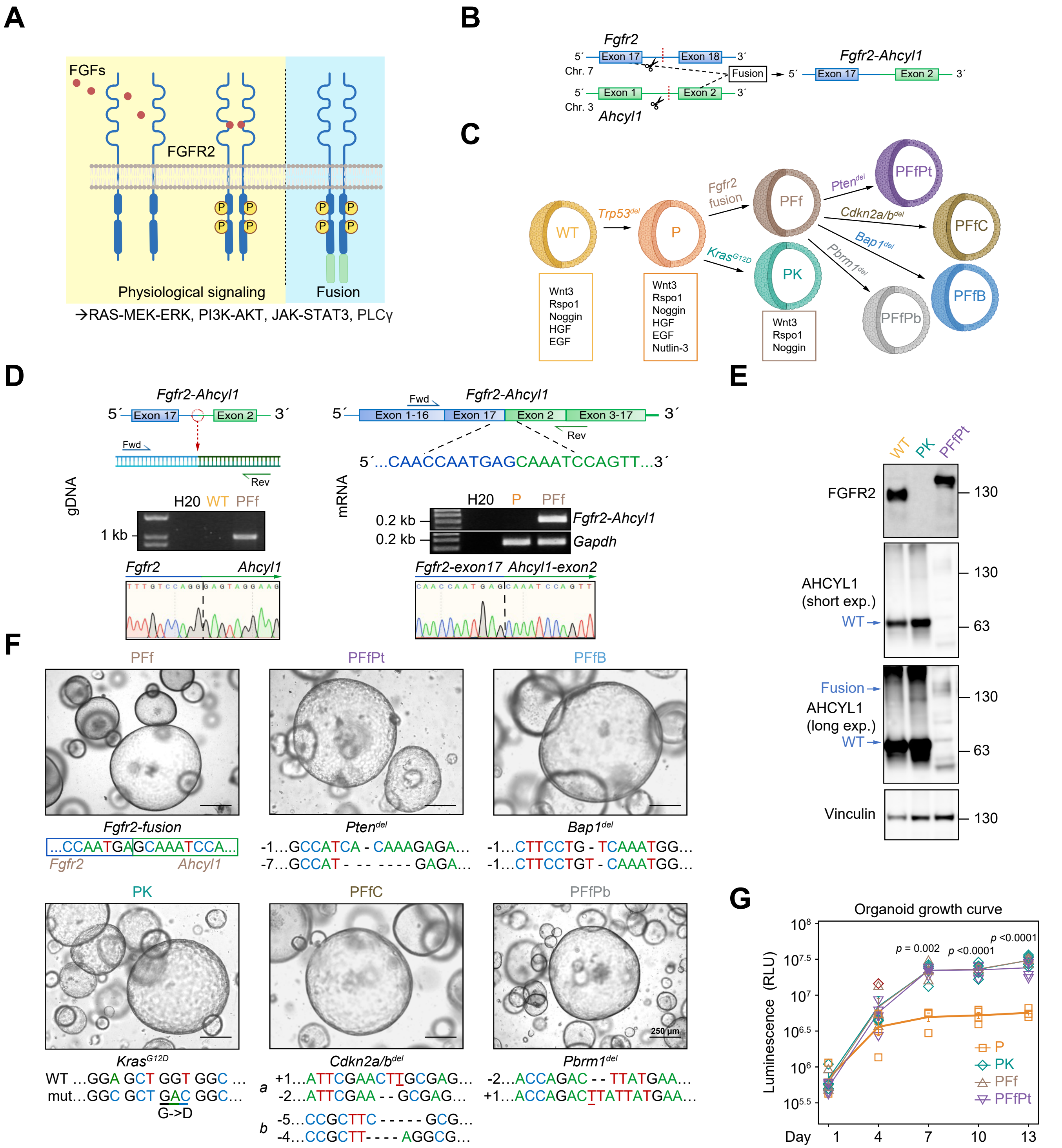

Fig. 1. Generation of endogenous FGFR2 fusion in murine cholangiocyte organoids. (A) Illustration of FGFR2 activation physiologically or via ligand-independent receptor dimerization and autophosphorylation due to the fusion partner. (B) Schematic of the CRISPR/Cas9 intron-targeting of murine *Fgfr2* and *Ahcyl1* to generate the chromosomal rearrangement that results in the *Fgfr2-Ahcyl1* fusion gene. (C) Illustration of the sequential genome editing of wildtype murine cholangiocyte organoids via plasmid transfection to engineer FGFR2 fusion-driven CCA organoids. Abbreviations of altered genes: *Trp53* deletion (P), *Fgfr2-Ahcyl1* fusion (Ff), *Kras*^{G12D} (K), deletion of *Pten* (Pt), combined *Cdkn2a/b* (C), *Bap1* (B), and *Pbrm1* (Pb). (D) PCR and Sanger sequencing results of genomic DNA and mRNA to confirm the rearrangement at the expected exon-exon junction. (E) Western blot analysis of FGFR2 and fusion partner AHCYL1 indicating the respective size shift in the PFPt organoid line (exp. = exposure). (F) Microscopic bright-field images of generated CCA organoid lines as indicated, with similar morphological aspect and respective genetic alterations specified below. (G) Cell viability assay of indicated organoid lines upon withdrawal of growth factors over time. n = 4 biological replicates; the connecting line indicates mean ± SEM; significance was considered with threshold p <0.05; exact p values are shown in the figure (one-way ANOVA test to compare lines P *vs*. PFf).

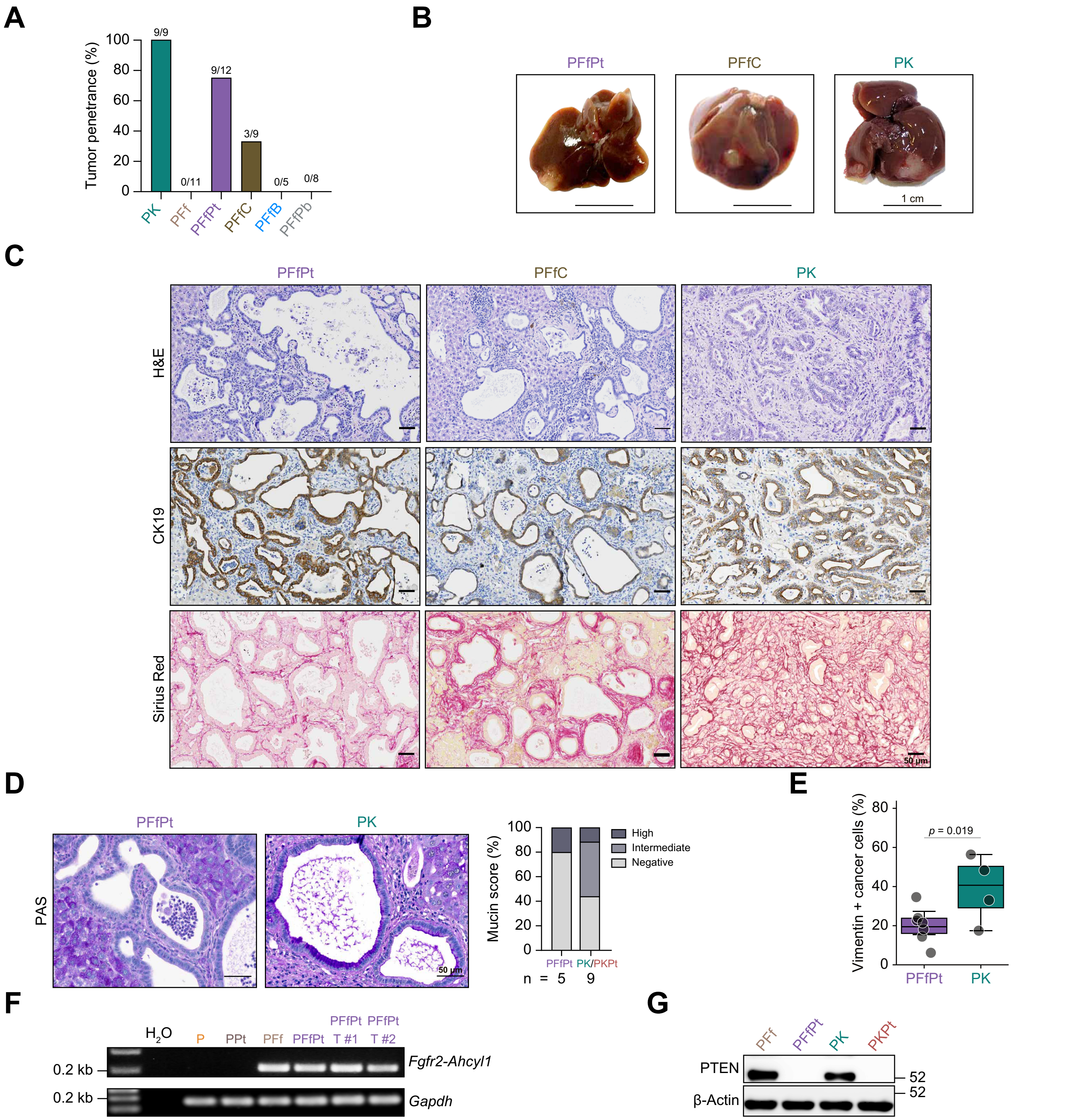

Fig. 2. Murine FGFR2 fusion-driven organoids develop iCCA in syngeneic hosts. (A) Bar plot representing percentage of tumor penetrance for indicated lines upon intrahepatic implantation with 2–5 × 10^5 cells for PK and 0.5–1 × 10^6 cells for other lines (absolute tumor numbers displayed on top of the bar). (B) Representative macroscopic images of iCCA development for the respective lines. (C) Representative histologic pictures of PFfC, PFfPt, and PK iCCA with stains as indicated. (D) Representative PAS stain images of PK and PFfPt iCCA displaying mucin production at the apical membrane of PK cancer cells. Stacked bar plots of semiquantitative assessment of mucin in PFfPt and KRAS-driven (PK and PKPt) iCCA (n = 5 and 9). All scale bars indicate 50 µm. (E) Comparison of vimentin-positive cancer cell percentage. Box indicates IQR and whiskers 1.5xIQR with bold line median; each data point indicates a tumor; p = 0.019 (binomial generalized linear mixed-effects model on four animal replicates each). (F) Gel electrophoresis of successfully amplified *Fgfr2-Ahcyl1* mRNA by RT-PCR in original and re-cultured tumor-derived (T#1 and 2) organoid lines indicating persistent expression of the FGFR2 fusion. (G) Western blot confirming PTEN deletion in the indicated tumor-derived organoid lines.

PFfC containing *Cdkn2a/b* deletion led to successful Ff-iCCA formation at a rate of 75% (nine of 12) and 33% (three of nine), respectively (Fig. 2A,B). Compared with PK tumors, the growth rate was slower for PFfPt and PFfC tumors, and they were typically harvested at 20–30 weeks following injection. In the mice that developed tumors, typically from one to five separate tumor nodules could be microscopically observed. A thorough histopathologic analysis revealed that all tumors were consistent with iCCA, presenting with a well-differentiated glandular growth pattern in a desmoplastic stroma reaction and a strong positivity of cancer cells for the cholangiocellular marker CK19 (Fig. 2C). Compared with PK iCCA, Ff-iCCAs showed a more pronounced glandular pattern with sometimes larger lumina, reminiscent of small duct type iCCA, and an increased accumulation of immune cells at the tumor–liver interface. Mucin production, a typical feature of large duct type iCCA, was almost exclusively prevalent in PK iCCA (Fig. 2D). The percentage of vimentin-positive cancer cells as a marker for epithelial-to-mesenchymal transition was markedly increased in PK iCCA compared with PFfPt iCCA (Fig. 2E). Upon tumor harvesting, the isolated cancer cells cultured as tumor organoids showed continuous growth in cell culture; furthermore, a sustained expression of the *Fgfr2* fusion transcript as well as PTEN knockout could be confirmed (Fig. 2F,G). Generally, these lines were stable and remained implantable after repeated freeze–thaw cycles.

For further analyses, we mainly focused on the PFfPt iCCA model in comparison with PK. To exclude that the tumorigenesis of PFfPt was primarily driven by *Pten* or *Cdkn2a/b* deletion, we created new lines that only shared *Tp53* and *Pten* or *Cdkn2a/b* deletion without *Fgfr2* fusion (PPt and PC) (Fig. S3A). Upon implantation, these lines did not result in liver tumor formation within an observation period of 20–30 weeks (zero of four and zero of six, respectively) (Fig. S3B), suggesting that these tumor suppressors are not the main oncogenic drivers in our Ff-iCCA model.

FGFR2 fusion-driven iCCA is less immune-evasive than KRAS-driven iCCA

As the growth of Kras-mutated and Ff-driven cholangiocyte organoid lines in cell culture without supplements was indistinguishable (Fig. 1G), we hypothesized that either host factors or the interplay with TME was responsible for the lack of tumorigenicity of PFf organoids. Therefore, we implanted PFf organoids in the livers of immunodeficient NOD scid gamma (NSG) mice and observed tumor formation at a penetrance of 60% (3/5) within 12–20 weeks (Fig. 3A). The morphology of the cancer epithelium was comparable to the PFfPt line in wildtype C57Bl/6J mice, with a pronounced glandular growth pattern that occasionally exhibited a prominent cystic aspect; the mean proliferation rate was 3.5%, as assessed via Ki67 positivity (Fig. 3B,C).

Multiplexed spatial TME profiling reveals oncogenic pathway-specific immune infiltration architecture

As the results from the implantation experiment in NSG mice suggest that the immune system plays a crucial role in

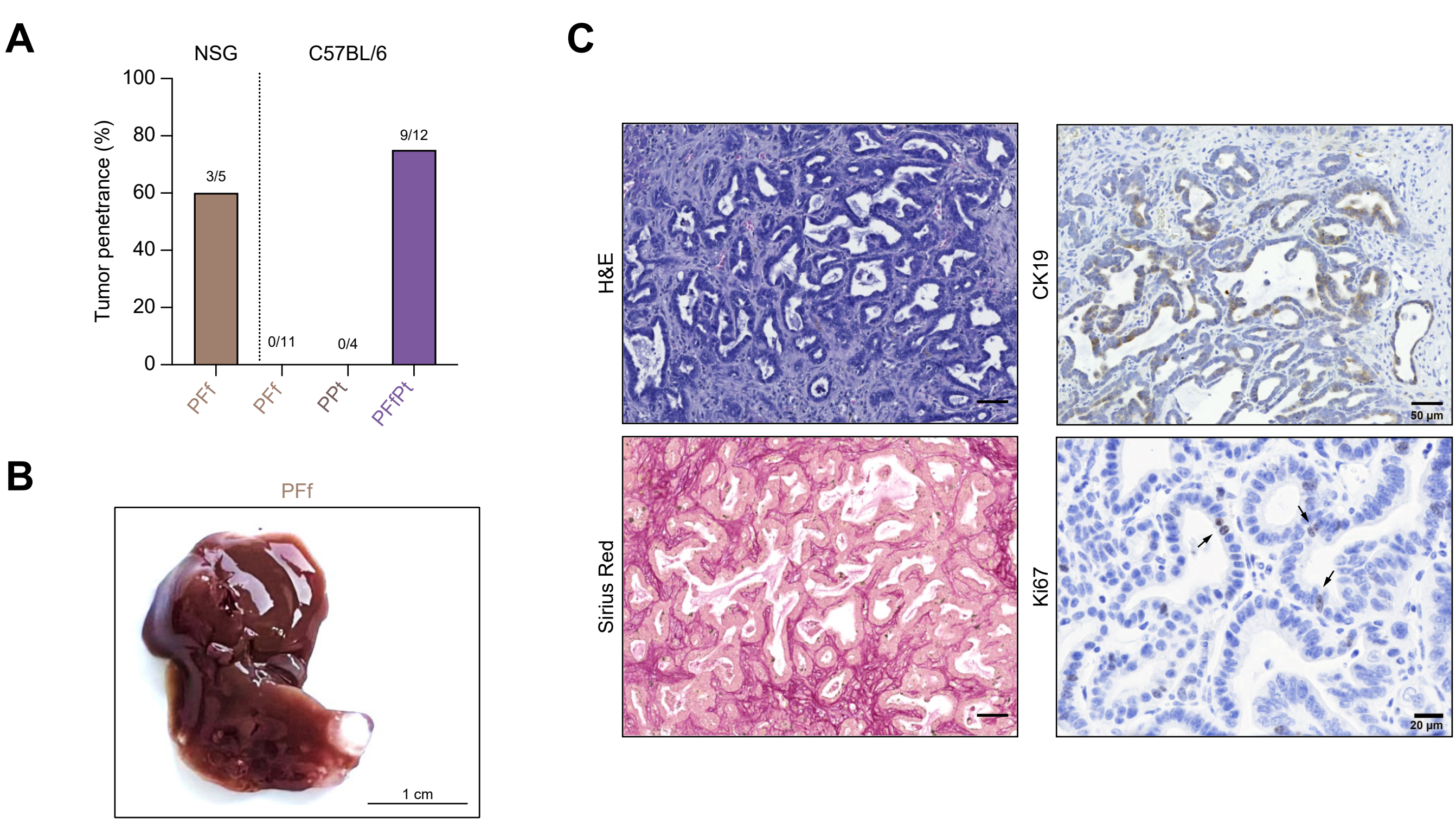

Fig. 3. FGFR2 fusion-driven iCCA is less immune-evasive than KRAS-driven iCCA. (A) Bar plot representing percentage of tumor penetrance for indicated lines upon intrahepatic implantation; 0.5–1 × 10^6 cells were used for implantation. (B) Representative macroscopic image of PFf iCCA in the liver of NOD scid gamma (NSG) mice. (C) Representative histologic pictures of PFf iCCA in NSG liver with stains as indicated. Black arrows indicate Ki67-positive cancer cells. Scale bars = 50 or 20 µm.

preventing tumorigenesis of PFf iCCA, and as more immune cell presence was observed in PFfPt iCCA, we aimed at investigating the tumor immune microenvironment (TIME) in more detail. We performed multiplexed ion beam imaging (MIBI), a spatial proteomic approach, with a 30-plex antibody panel including key lineage markers for immune cells, cancer-associated fibroblasts (CAFs), endothelial cells, and cancer cells (Fig. 4A). We acquired 327 fields of view each at a resolution of 390 nm from seven PFfPt and four PK iCCA tumors from four mice, yielding a dataset of ~248,600 cells with a mean of ~22,500 cells per tumor (Fig. S4A). More than 80% of cells per section were confidently assigned to a defined cell type, and segmentation maps closely reflected marker intensities (Fig. S4B). Cell lineage identities were assigned hierarchically based on the lineage marker expression patterns (Fig. 4A,B and Fig. S4A and B), enabling a comprehensive analysis of TME composition.

First, we compared the two tumor types in terms of spatial organizations at the tissue level (Fig. 4C and Fig. S5A). PK iCCA tumors were larger, with a well-defined tumor border and a distinct periphery and core tissue architecture (described in detail by Liu *et al.*[22]), where immune and stromal cells were predominantly enriched in the tumor periphery. In the generally smaller PFfPt iCCA tumors, cancer cells formed slightly more cystic structures surrounded by immune and stromal cells. Within the immune compartment, $CD11b^+$ $CD11c^+$ dendritic cells accounted for a greater proportion in PK tumors, whereas $Ly6G^+$ neutrophils and cytotoxic T (T_c) cells were more represented in PFfPt tumors (Fig. 4D, Fig. S4C). Among immune cells, only non-M2 macrophages showed a significantly higher $Ki67^+$ proportion in PK tumors (Fig. S4E). Spatial cell density analysis revealed directionally consistent differences for these populations, although only T_c cells reached statistical significance, likely reflecting the limited sample size (Fig. 4E). Both SMA^+ and SMA^- CAFs were present at higher densities in PFfPt than in PK tumors, with the difference being slightly more pronounced for SMA^- CAFs, though neither reached statistical significance (Fig. 4F). Despite these compositional differences, expression levels of PD-L1 in cancer cells, PD-1 in T_c and regulatory T (T_{reg}) cells, and CTLA-4 in T_{reg} cells were comparable between PFfPt and PK tumors (Fig. S4F), suggesting that the two tumor types share a similar immune checkpoint expression landscape.

Conversely, the two iCCA subtypes displayed strikingly divergent cellular interaction landscapes (Fig. 4G). Shared interactions in both cancer types were largely confined to stromal populations, including fibroblast and endothelial cell pairs, as well as cancer cell interactions with the immediate stromal niche. Immune and CAF interactions in PFfPt iCCA tumors were predominantly mediated by SMA^- CAFs, whereas SMA^+ CAFs drove the corresponding interactions in PK tumors. Among rewired interactions between immune cells, $CD11c^+$ $CD11b^{+/-}$ dendritic cells acquired numerous interaction partners in PK tumors, whereas $Ly6G^+$ neutrophils gained interaction partners predominantly in PFfPt tumors. With respect to cancer and immune crosstalk, T_c cells targeted cancer cells in both cancer types, although at markedly higher densities in PFfPt tumors (Fig. 4E,H). Cancer cell interactions were further distinguished by cancer type: in PFfPt tumors, cancer cells specifically interacted with M2-like macrophages, $Ly6G^+$ neutrophils, and $CD11c^+$ cells, whereas in PK tumors, cancer cell interactions involved $CD11c^+$ cells in the reverse direction and $F4/80^{med}$ myeloid cells. Collectively, $Ly6G^+$ neutrophils stood out as a population differing between tumor types in both interaction connectivity and cell density (Fig. 4E,G,H and Fig, S5B,C).

Neutrophil infiltration is not driven by *Pten* deletion

We next generated a PK line harboring an additional *Pten* deletion, which readily developed iCCA upon implantation in C57Bl/6J mice. We used this line to control for PFfPt and potential immune-modulating effects of aberrant PI3K/Akt signaling in these *Pten* knockout cells (Fig. 5A and Fig. S6A–C). We first assessed the proliferation rates in all three lines upon implantation, which were significantly increased in the two KRAS-driven iCCA subtypes (mean proliferation rates of 1.2% in PFfPt, 2% in PK, and 3.5% in PKPt; Fig. 5B). Similar to our comparisons with PK iCCAs, we observed strong differences in immune cell infiltrates between PFfPt and PKPt iCCAs already by light microscopy. Consistent with the observations in the sample subset from MIBI analysis, both KRAS-driven iCCA typically showed lower infiltrates of T_c cells and Ly-6G/Ly-6C+ neutrophils (Fig. 5C,D). Infiltration by neutrophils has recently been identified as an exclusive TME feature in Ff-iCCA in humans.[23,24] We aimed at independently validating this association in a retrospective analysis from the LCCH patient registry, comparing Ff-iCCA with iCCA harboring other drivers. In accordance with prior descriptions, we could observe higher neutrophil cell densities in human Ff-iCCA (Fig. 5E). Owing to low sample size, statistical testing was not applied. Thus, we could validate our MIBI-based observations of an increased infiltration of T_c cells and neutrophils and confirm this effect as independent of *Pten* deletion.

PFf organoids induce a chemotaxis-enriched transcriptional program and respond to FGFR inhibitors

The murine amino acid sequence in the kinase domain of FGFR2 shares a >99% homology with the human FGFR2 protein (Fig. S7). Accordingly, treatment with the FGFR inhibitors pemigatinib and futibatinib led to a statistically significant reduction of PFf cholangiocyte survival in a colony-forming assay in all tested dosages compared with DMSO control, whereas PK CCA cells remained unaffected (Fig. 6A and Fig. S8A). An FGFR2 fusion-specific inhibition could also be observed in a cell viability assay of these two organoid lines (Fig. 6B). The inhibitory efficacy was hereby similar to that described for some patient-derived Ff-iCCA cell lines.[15,25,26] To explore molecular differences between PFf and PK, we performed bulk RNA sequencing across six different organoid conditions: PFf and PK organoids treated either with DMSO as a control, pemigatinib, or futibatinib (n = 3 per condition). Principal component analysis revealed that the dominant axis of variation (PC1) separated organoids by oncogenic activation (93%), whereas PC2 (4%) separated PFf, but not PK organoids by FGFR inhibition (Fig. 6C). Moreover, 3,735 genes were differentially expressed in PFf relative to PK (Fig. 6D,E and Table S4). FGFR inhibition revealed 1,742 genes with a PFf-specific transcriptional response, which we considered as *bona fide* FGFR2 target genes, including well-described targets such as *Etv4* and *Dusp6* (Table S5). Demonstrating the specificity of the FGFR

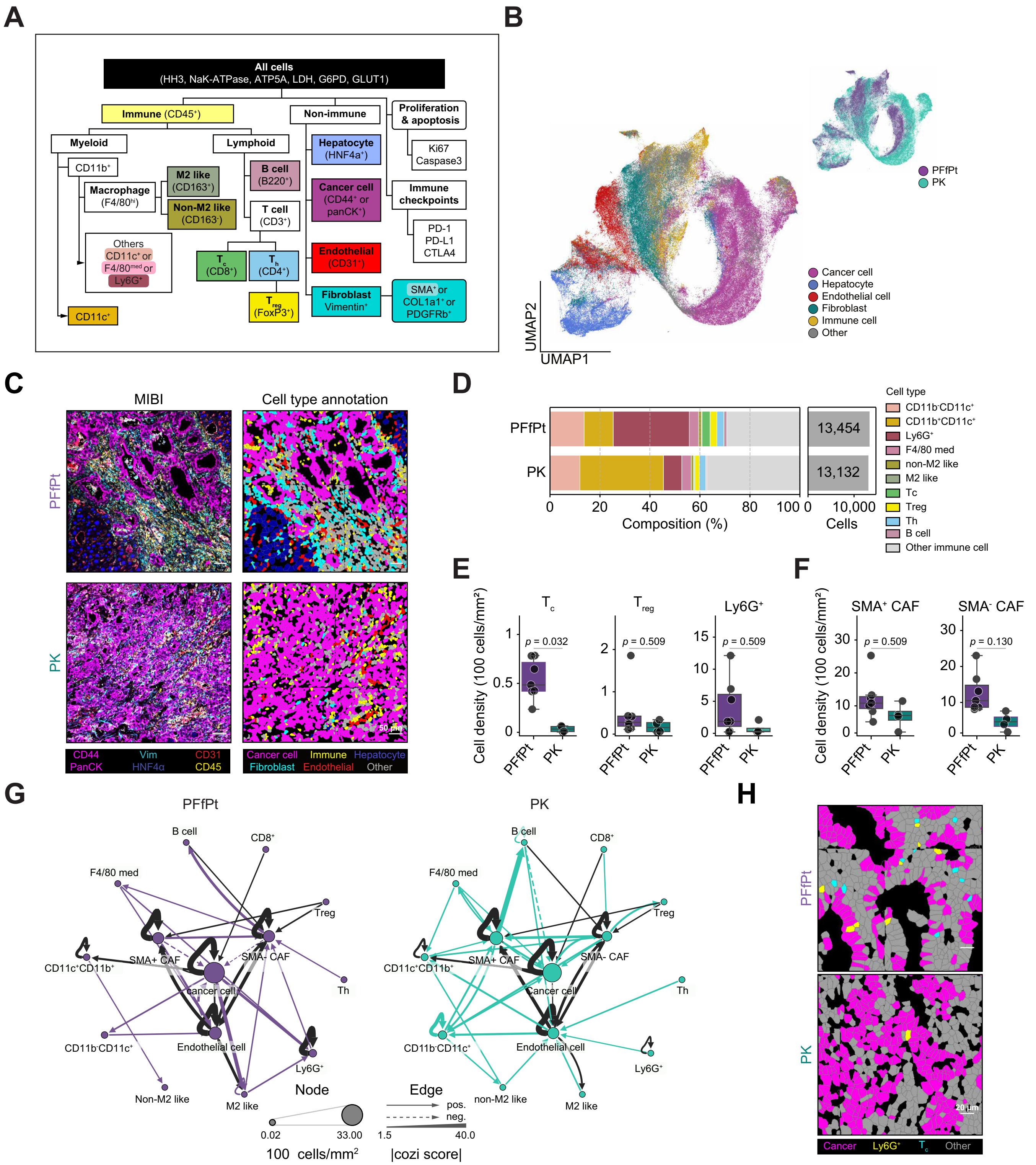

Fig. 4. Multiplexed spatial profiling reveals oncogenic pathway-specific immune infiltration architecture. (A) Hierarchical MIBI panel overview comprising 30 antibodies targeting cellular structures, cell lineage and phenotypic markers, cell fate indicators, and immune checkpoint molecules. (B) UMAP projection of single cells based on lineage marker expression profiles. Each point represents one cell (~248,600 cells in total from seven PFfPt (purple) and four PK (turquoise) tumors from four mice each), colored by major cell lineage annotation as indicated. (C) Representative tissue images of PFfPt and PK tumors displaying lineage markers (left) and the corresponding cell lineage annotations (right). (D) Cellular composition of the immune compartment in PFfPt and PK tumors. (E, F) Cell densities of indicated immune cell (E) and CAF (F) populations. Box indicates IQR and whiskers 1.5xIQR with bold line median; each data point indicates a tumor; exact *p* values are shown in the figure (binomial generalized linear mixed-effects model on four animal replicates each and a significance threshold of $p < 0.05$). (G) Cell-type interaction networks for PK and PFfPt tumors, constructed from pairwise COZI scores (after exclusion from hepatocytes and other uninformative cell populations). Edges represent statistically significant interactions. For PK tumors, the analysis was restricted to the tumor periphery, where immune cells were predominantly enriched relative to the tumor core. (H) Representative tissue images highlighting the spatial arrangement of cancer cells, Ly6G+ neutrophils, and CD8+ T cells.

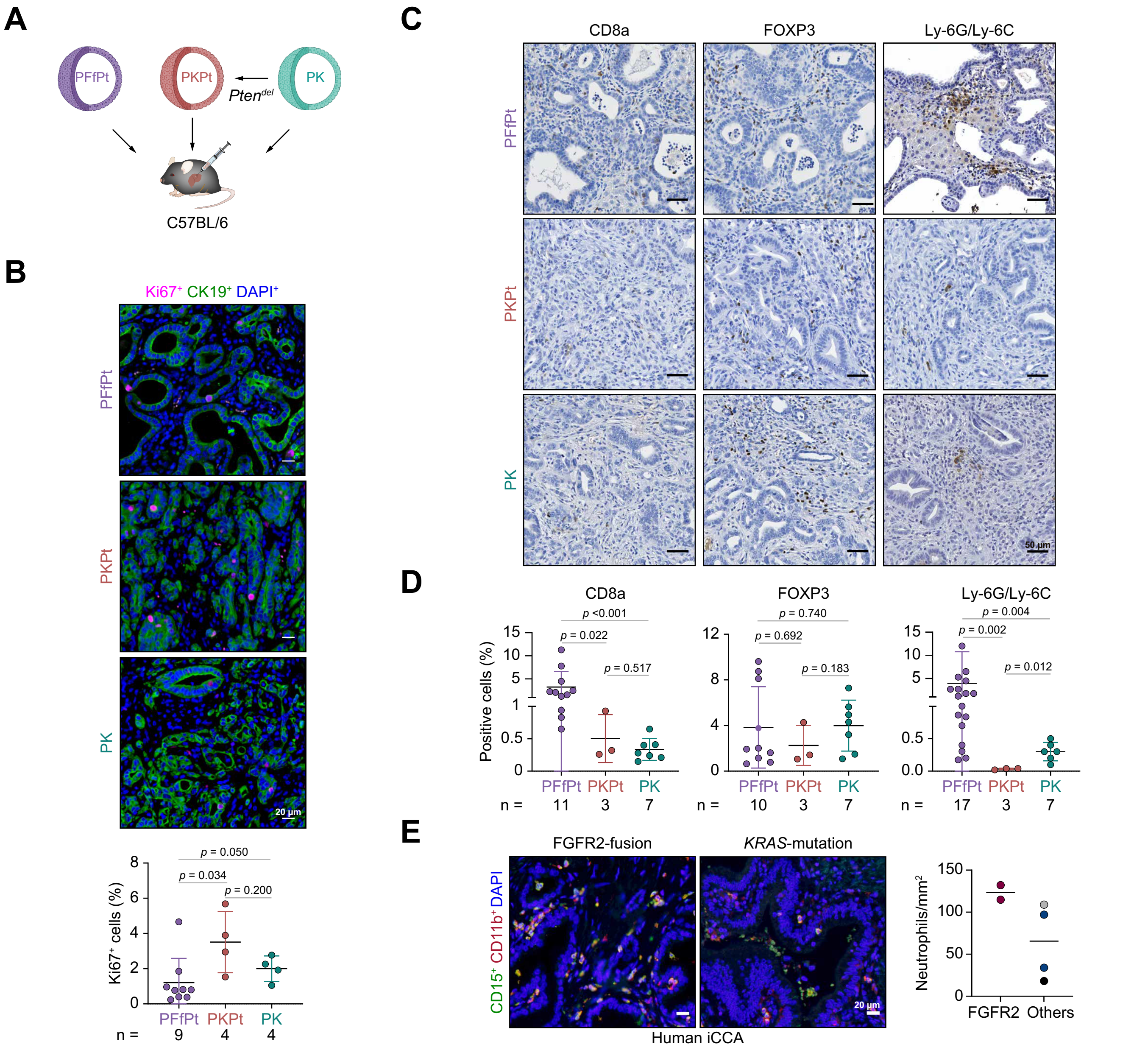

Fig. 5. FGFR2 fusion-driven iCCA show more immune cell infiltrates than KRAS-driven iCCA. (A) Schematic illustrating iCCA models; 2–5 × 10^5 cells were implanted for PK and PKPt lines and 0.5–1 × 10^6 for PFfPt. (B) Representative immunofluorescence images for Ki67 and Ck19 with DAPI. Scale bars = 20 µm. Quantification of Ki67$^+$ cancer cells depicted as scatter plot with each data point indicating one animal replicate: PFfPt (n = 9), PKPt (n = 4), PK (n = 5). (C) Immunostaining images for CD8a, FOXP3, and Ly-6G/Ly-6C in PFfPt, PKPt, and PK iCCA. Scale bars = 50 µm. (D) Scatter plots depicting percentage of cells positive for the indicated marker whole tumor areas per section with each data point indicating one animal replicate: PFfPt (n = 11), PKPt (n = 3), and PK (n = 7). (E) Representative immunostaining images of human FGFR2 fusion-driven iCCA and one with *KRAS* mutation. CD11b$^+$/CD15$^+$ cells were counted as neutrophils. Dot plot showing the mean neutrophil density of 10 high-power fields per tumor section, comparing iCCA harboring a FGFR2 fusion or other mutations. Each data point represents one tumor. Lines in plots of (B) and (D) indicate mean ± SD; exact *p* values are shown in the figure (Mann-Whitney *U* test).

inhibitors, only one gene was significantly differentially expressed in PK organoids upon treatment. Gene set enrichment analysis revealed in PFf an enrichment of basic cellular and metabolic processes (RNA/protein processing and metabolism); interestingly, the terms “humoral immune response” and “chemotaxis” ranked as the third and fourth most significant hallmarks after reducing semantic redundancy among the enriched terms (Fig. 6D). Focusing in more detail on chemotaxis-related pathway terms, all of them displayed as significantly enriched in PFf over PK, and the top 10 leading-edge genes of the “chemotaxis” gene set were all significantly overexpressed in PFf, demonstrating a robust phenotype (Fig. S8B). We next specifically investigated most common chemokines for T_c cells (*Cxcl9* and *Cxcl10*) and neutrophils (*Cxcl1*, *Cxcl2,* and *Cxcl5*).[27] Among them, *Cxcl10*, *Cxcl2*, and especially *Cxcl1* were robustly and significantly overexpressed in PFf (Fig. 6F). When looking at the interaction with FGFR inhibition, to identify the key cellular programs

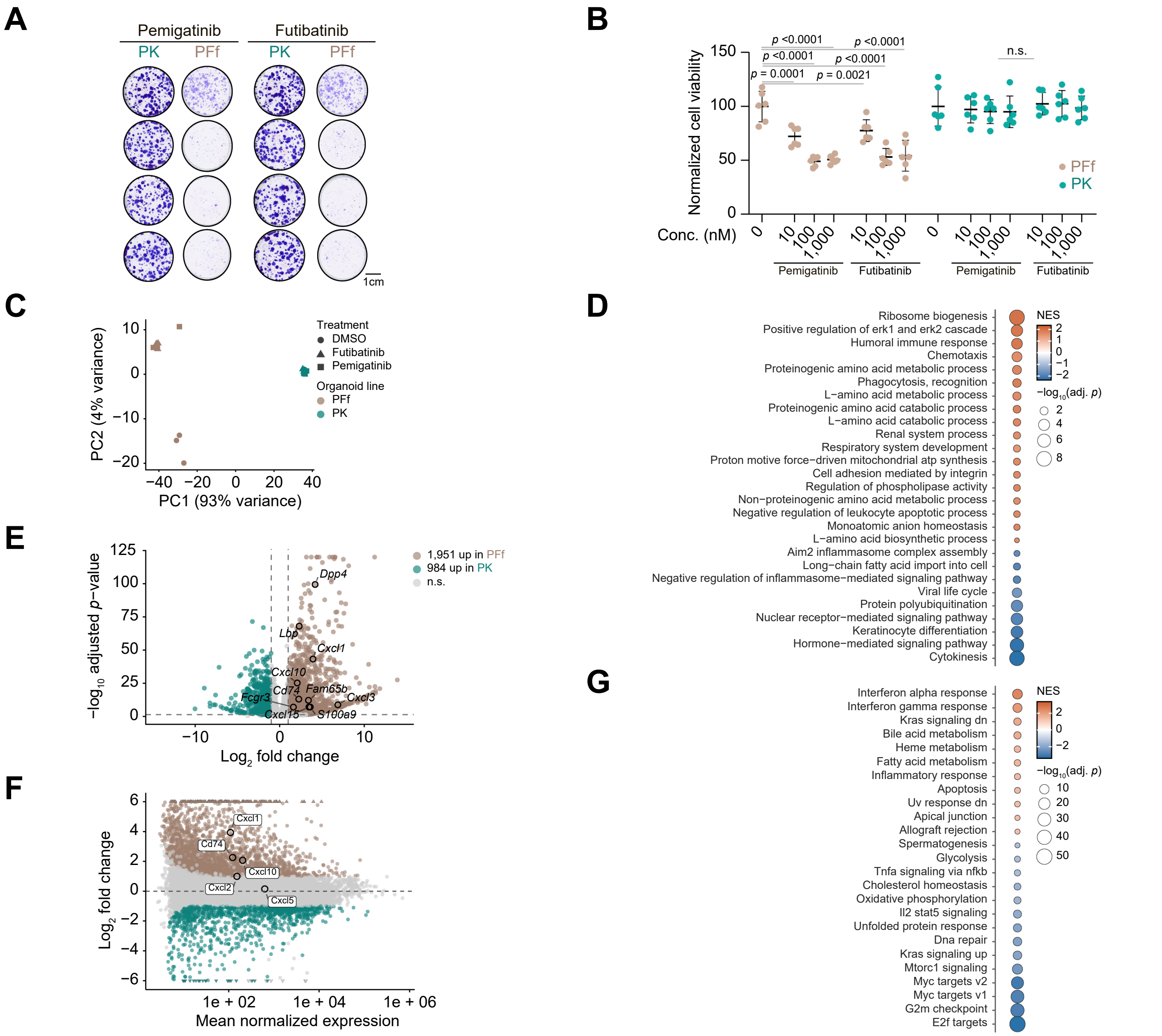

Fig. 6. FGFR2 fusion-driven CCA organoids show an increased chemotaxis signature and are susceptible to FGFR inhibitors. (A) Representative crystal violet-stained images of wells from colony-forming assay of PFf and PK cell lines seeded as a monolayer and treated with the indicated concentrations of pemigatinib and futibatinib for 10 days. (B) Cell viability assay of PFf and PK organoids treated with the indicated concentrations of pemigatinib and futibatinib normalized to DMSO control. n = 6 per condition with line indicating mean ± SD; exact p values are shown in the figure, with $p \geq 0.05$ considered not significant (one-way ANOVA with Welch's correction). (C) Principal component analysis (PCA) plot of variance-stabilized expression profiles from the indicated lines and treatment conditions. PC1 (93% variance) and PC2 (4%) separate samples primarily by oncogenic pathway activation and, to a lesser extent, by FGFR inhibition (in PFf only). (D) Gene set enrichment analysis (GSEA) of Gene Ontology (GO) Biological Process terms, reduced by semantic similarity, highlights enrichment of immune and chemotaxis-related processes in PFf alongside metabolic and proliferative differences between DMSO-treated PFf and PK. (E) Differential gene expression between DMSO-treated PFf and PK shown as volcano plot of protein-coding genes ranked by $\log_2$ fold change and $-\log_{10}$ adjusted p value. Selected leading-edge neutrophil chemotaxis genes are labeled. Values above $-\log_{10}$ adjusted p value = 120 were capped for visualization. (F) MA plot of differential expression between DMSO-treated PFf and PK organoids, showing mean normalized expression *vs.* $\log_2$ fold change. Selected chemotaxis-associated genes are highlighted. Log_2 fold changes were capped at ±6 for visualization. (G) Hallmark GSEA reveals a PFf-selective shift after FGFR inhibition, with reduced growth and cell cycle programs and relative enrichment of immune and inflammatory signatures.

affected via FGFR2, interferon alpha and gamma response were the two top upregulated terms, whereas expectedly cell cycle program (G/2M, E2F targets) as well as Myc activation and Kras signaling were among the top downregulated terms (Fig. 6G). Interestingly, neither "chemotaxis" term nor expression of its related genes was significantly altered upon FGFR inhibition, suggesting that the upregulation of these genes in PFf *vs*. PK cannot be explained directly via canonical signaling pathway, or that compensatory mechanisms are in play.

FGFR inhibition in PFfPt iCCA is antiproliferative but without apparent immunomodulatory effects

To assess the effects of FGFR inhibition in our Ff-iCCA model, we treated mice with pemigatinib for 2 weeks once tumors had developed (n = 7 control and 5 treated; Fig. 7A). Although we observed no differences in tumor morphology or increased necrotic areas between treated and untreated PFfPt tumors, we confirmed a pharmacodynamic effect via significantly reduced proliferation rates and intratumoral downregulation of the canonical FGFR target gene *Etv4*, which was among the top downregulated genes in the bulk RNA sequencing analysis (Fig. 7B,C and Table S5). Focusing our immune infiltrate analysis on T_c and T_{reg} cells, as well as Ly-6G/Ly-6C+ neutrophils, we could not detect significant differences between the control and treated tumors (Fig. 7D). In parallel, there was also no evidence of increased T_c cell activity or checkpoint engagement based on granzyme B or PD-1 positive T_c cell assessment upon FGFR inhibition (Fig. 7E). Consistent with our results from the bulk RNA sequencing on treated PFf organoids and the observed lack of immune infiltration patterns upon FGFR inhibition, the mRNA expression of most chemokines we assessed as relevant for T_c cell and neutrophil attraction were intratumorally not changed (Fig. 7F). The only robustly downregulated chemokine was *Cxcl10*, an effect we did not observe in PFf organoids *in vitro* and potentially explained via another cellular source in TME. In summary, FGFR inhibition *in vivo* leads to measurable cancer cell-specific effects in this endogenously engineered murine FGFR2 fusion model. However, we did not observe any major changes in neutrophil and T-cell infiltration and activity as a surrogate for an immunomodulatory effect.

Discussion

Here, we introduce a new organoid-based iCCA preclinical model that allows us to reproduce co-alterations frequently observed in human iCCA through systematic genetic engineering. This approach offers versatility and uses cholangiocytes as the cell of origin in contrast to many widely used iCCA models relying on transforming hepatocytes via transposon- and viral vector-based systems.[28] Typically, these systems also have to rely on oncogenes that drive towards biliary lineage, which limits the range of alterations that can be explored. The organoid-engineering approach used here profits from genetic flexibility with additive mutations, as first described in human cholangiocyte organoids.[29] However, it requires orthotopic implantation and, therefore, may be less suitable for assessing the aspects of early carcinogenesis compared with autochthonous models.

Since the first conception of small duct and large duct type iCCA based on pathomorphological criteria in 2010,[30] they have become widely accepted as biologically distinct and prognostically relevant entities, now incorporated into the 5th WHO classification and clinical practice guidelines.[31] The genomic landscape of iCCA has further strengthened this classification by reflecting the enrichment of different driver mutations.[10,31] *FGFR2* rearrangements are considered exclusive to small duct type iCCA, whereas KRAS-driven iCCA is enriched in the large duct type.[10] Consistently, our model recapitulates these hallmarks in the respective cancer genotypes. Mucin production, for example, was almost exclusive to the KRAS-driven iCCA, which was overall the more aggressive tumor model. The prevailing hypothesis to explain the distinction of small and large duct type iCCA holds that they arise from different cells of origin reflecting the morphology and function of cholangiocytes in small *vs.* large ducts. This would in turn imply that these originally transformed cells have a propensity for specific genetic alterations, such as *FGFR2* rearrangements or *KRAS*-mutations, respectively. Interestingly, because our iCCA subtypes derive from the same parental cholangiocyte, our findings suggest that specific pathway activation alone can influence the pathomorphological phenotype. Interpretation is nonetheless limited, as transformation occurs in cholangiocyte organoids cultured *in vitro*, outside their physiological niche, and given the known plasticity of hepatobiliary epithelium.[21,32] Nevertheless, we strongly encourage further investigation into the relationship between cell of origin and oncogenic pathway activation in small and large duct type iCCA.

By achieving an endogenous rearrangement of *Fgfr2* in the mouse genome, our model closes an important gap in Ff-iCCA preclinical modelling as it enables investigations in a syngeneic system and detailed analysis of the tumor and TIME interplay. Although it seems theoretically advantageous to systematically study common co-occurring mutation patterns, such as *Bap1*, *Pbrm1*, *Cdkn2a/b*, and *Pten*, our efforts in this regard were substantially hampered by the lack of tumorigenicity of lines with co-deletion of *Bap1* or *Pbrm1*, and by variable penetrance rates (33–75%) in lines with co-deletion of *Cdkn2a/b* or *Pten*. Our *in vitro* data did not hint at any cell autonomous disadvantage compared with $Kras^{G12D}$-mutation driven iCCA lines; similarly, proliferation rates were only slightly increased in our KRAS-driven iCCA tumors. As PFf cholangiocyte organoids could grow in NSG mice, the key hurdle to overcome in our model may be antitumor immunity.

The increased immunogenicity of Ff-iCCA could be due to the neoantigen presentation of the fusion protein epitopes, which has been demonstrated in at least one human case.[33] However, it is more likely that oncogenic KRAS signaling, which was our standard comparative model in this work, has superior mechanisms for immune escape. $Kras^{G12D}$ mutation-harboring lines had a higher tumor penetrance and possibly generated very immune-excluded, *i.e.* "immunologically cold" iCCAs in our model. Differences in tumor growth kinetics or penetrance represent a possible alternative explanation for this finding. However, this is unlikely because our KRAS-driven iCCA model showed consistently low immune infiltrates across multiple time points, whereas genetic manipulation of KRAS effector genes was sufficient to reverse this low-infiltrate phenotype independent of changes in growth kinetics.[22] This immune-excluded phenotype is consistent with findings in human CCA, where co-occurrence of *Trp53*-and *Kras*-mutations were mostly associated in low immune enrichment classes (*i.e.* tumor classical, and desert-like).[34] Moreover, several immune escape mechanisms have been described for oncogenic KRAS signaling. These include the secretion of chemokines and cytokines to create an immunosuppressive TME, or the repression of interferon signaling, which may be relevant in our model.[35,36]

Ff-iCCA is a frequent CCA subtype amenable to targeted therapy. Although a subset of patients can profit from FGFR inhibitors with objective response rates observed in up to 42%,

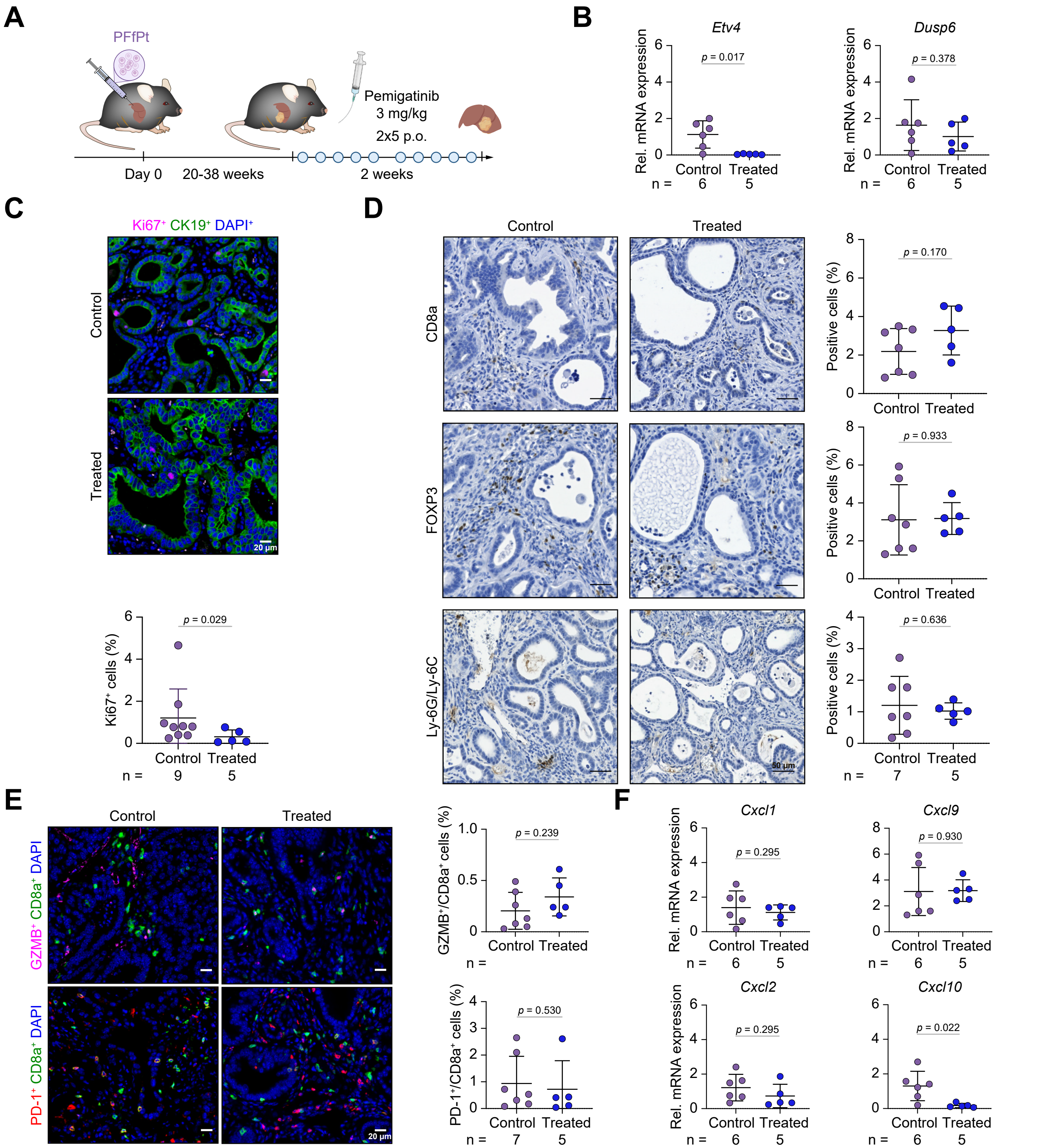

Fig. 7. FGFR inhibition in PFfPt iCCA is antiproliferative but without apparent immunomodulatory effects. (A) Schematic of experimental treatment design. (B) Normalized FGFR2 target gene expression from control PFfPt iCCA and treated with pemigatinib. Control (n = 6), treated (n = 5); exact *p* values are shown in the figure (Welch's *t* test). (C) Representative immunofluorescence images for Ki67 and Ck19 with DAPI. Scale bars = 20 μm. Quantification of Ki67-positive cancer cells depicted as scatter plot. Control (n = 9), treated (n = 5); *p* = 0.029 (Mann-Whitney *U* test). (D) Representative immunostaining images for CD8a, FOXP3, and Ly-6G/Ly-6C. Scale bars = 50 μm. Quantification of respective cell type abundance shown as a scatter plot. Control (n = 9), treated (n = 5); exact *p* values are shown in the figure (Welch's *t* test). (E) Immunostaining for T_c cells (CD8a+) co-expressing either granzyme B (GZMB) or PD-1. Scale bars = 20 μm. Scatter plots depict percentage of respective double-positive T_c cells per mouse. Control (n = 7), treated (n = 5); exact *p* values are shown in the figure using Welch's *t* test (GZMB) and Mann-Whitney *U* test (PD-1). (F) Intratumoral normalized mRNA expression of indicated chemokines shown as scatter plot. Control (n = 7), treated (n = 5); exact *p* values are shown in the figure (Welch's *t* test). For all plots, each data point represents one animal as a biological replicate, and lines indicate mean ± SD.

the duration of response is usually limited to less than a year and many patients remain primary non-responders.[11,12] Secondary resistance is mainly attributed to the acquisition of resistance mutations in the *FGFR2* gene,[6,7,37] but the reason for the variability of initial response remains unclear. With genomic data from the FIGHT-202 and FOENIX trials, comprehensive analyses of the prognostic value of co-mutations provided few insights.[6,7] One limitation is clearly that such analyses could be underpowered, particularly when looking at possibly weak effects of different co-alterations of a subtype of a relatively rare cancer. However, it may also reflect the actual predictive value of the cancer genome by itself, as it insufficiently informs on the downstream biological effects and the associated host responses that could influence treatment response. This is of particular importance when trying to develop combination treatment including immunotherapy strategies.

Interestingly, of all co-alterations tested in our Ff-iCCA model, additional *Pten* deletion was most successful to overcome this obstacle to tumor formation. Because *Pten* knockout itself is not a main oncogenic driver in our model, it is likely that this co-alteration supports immune escape mechanisms. *Pten* deficiency reportedly leads to an immunosuppressive TME and is associated with various immune-evasive pathways, such as reducing T-cell infiltration, interferon response, or activating STAT3-mediated signaling via PI3Kβ,[38–40] effects which could be reversed pharmacologically with PI3Kβ inhibitors or re-introduction of PTEN via lipid nanoparticles.[39,41] PTEN co-alterations in Ff-iCCA are not the most common ones with a frequency of ~3–5%,[5,6] but a combinatory targeted approach could be worth exploring in this subgroup.

One of the major phenotypes observed in our Ff-iCCA model is the strong infiltration of myeloid cells positive for Ly6G/C, a classic marker for neutrophils. Most importantly, neutrophil infiltration has very recently been identified as an exclusive TME feature in Ff-iCCA in humans, a trend that was similarly observed in our own human cohort.[23,24] The association of neutrophil infiltration with human Ff-iCCA has yet to be functionally proven. Our transcriptome analysis revealed that an increased expression of common chemotaxis genes was one of the most distinct features of PFf *vs.* PK CCA organoids. However, FGFR inhibition did not affect their expression levels; accordingly, we did not detect a difference in neutrophil infiltration upon pemigatinib treatment *in vivo*. Interestingly, FGFR inhibition in PFf led to a significant upregulation of interferon-stimulated genes. These findings are consistent with a recent report finding that activated FGFR signaling is blocking the Jak-STAT pathway downstream of IFNγ in renal cell carcinoma, which can be reactivated upon treatment with lenvatinib, a receptor tyrosine kinase inhibitor with partial anti-FGFR activity.[42] Given that interferon signaling is among the most potent inducers of chemotaxis genes,[43] whether such a compensatory mechanism could explain the persistently high expression of chemotaxis genes in Ff-iCCA despite FGFR inhibition remains to be investigated. Taken together, although our results cannot establish a definitive causal relationship between FGFR2 signaling and neutrophil recruitment, they confirm this strong association in an immunocompetent preclinical model and we encourage further investigation.

In summary, we have generated a syngeneic Ff-iCCA model that allows the functional study of cancer cell and TIME interplay. Our tumor model recapitulates key morphologic features of human Ff-iCCA and reproduces a neutrophil-enriched TME. Notably, this neutrophil association was not acutely reversed by FGFR inhibition. How oncogene-specific cancer cell programs establish and sustain this functional state of the TIME, and whether it can be therapeutically modulated, remain open questions that this model is positioned to address.

Affiliations

[1]*German Cancer Research Center (DKFZ) Heidelberg, Experimental Hepatology, Inflammation and Cancer, Heidelberg, Germany;* [2]*Medical Faculty Heidelberg, Heidelberg University, Heidelberg, Germany;* [3]*Faculty of Biosciences, Heidelberg University, Heidelberg, Germany;* [4]*Department of Medicine IV, Gastroenterology, Hepatology and Infectious Diseases, Heidelberg University, Heidelberg, Germany;* [5]*Liver Cancer Center Heidelberg (LCCH), Heidelberg, Germany;* [6]*German Cancer Research Center (DKFZ) Heidelberg, Systems Immunology and Single-Cell Biology, Heidelberg, Germany;* [7]*German Cancer Research Center (DKFZ) Heidelberg, Computational and Molecular Prevention, Heidelberg, Germany;* [8]*Institute of Pathology, Heidelberg University Hospital, Heidelberg, Germany;* [9]*German Cancer Consortium (DKTK), DKFZ Core Center, Heidelberg, Germany*

Abbreviations

CAFs, cancer-associated fibroblasts; Ff-iCCA, FGFR2 fusion-driven iCCA; FGFR2, fibroblast growth factor receptor 2; iCCA, intrahepatic cholangiocarcinoma; LCCH, Liver Cancer Center Heidelberg; MIBI, multiplexed ion beam imaging; NSG, NOD scid gamma; T_c cells, cytotoxic T cells; TIME, tumor immune microenvironment; TME, tumor microenvironment; T_{reg} cells, regulatory T cells.

Financial support

NL was supported by a Heinrich F. C. Behr scholarship. YLW was supported by a DKFZ Postdoctoral Fellowship. This work was supported by the Clinician Scientist Program of the Medical Faculty of Heidelberg University to AO; by the Initiative and Networking Fund of the Helmholtz Association under the Helmholtz Young Investigator Program (VH-NG-1605), the European Commission (ERC Starting grant (101116823), the State Parliament of Baden-Württemberg for the Innovation Campus Health + Life Science Alliance Heidelberg Mannheim, the Rolf M. Schwiete Stiftung, the Hector Foundation, and the Deutsche Forschungsgemeinschaft (DFG, German Research Foundation) under Germanys Excellence Strategy – EXC-3018/1 – 533587280 to FJH; the Rolf M. Schwiete Stiftung, and a Max Eder grant (70113858) from the German Cancer Aid to MTD.

Conflict of interest

The authors declare no conflicts of interest that pertain to this work.

Please refer to the accompanying ICMJE disclosure forms for further details.

Authors' contributions

Conceptualization: MTD. Methodology: NL, TKD, AO, YLW, CSL, MTD. Validation: NL, TKD, AO, YLW, CSL, MTD, AS, TL. Formal Analysis: NL, TKD, AO, YLW, CSL, DK. Investigation: NL, TKD, AO, YLW, CSL. Resources: AS, FJH, TL, MTD. Writing – original draft: NL, MTD. Writing – review and editing: NL, TKD, AO, YLW, CSL, DK, AS, FJH, TL, MTD. Visualization: NL, TKD, AO, YLW, CSL, MTD, DK. Supervision and project administration: MTD. Funding acquisition: NL, AO, YLW, FJH, MTD.

Data availability

The bulk RNA sequencing data generated in this study have been deposited in the European Nucleotide Archive (ENA) under accession number (PRJEB113177)

and the MIBI spatial proteomics data have been deposited in the BioImage Archive under accession number (S-BIAD2557).

Acknowledgments

The authors thank all patients and their families who provided biomaterial for this research project. The authors are very grateful to Sarah Böhn and Sabrina Schweiggert for their outstanding technical support, and to all laboratory members for productive discussions. The authors thank Robert Vanner, Sven Truxa, Kassandra Hoetzel, and Carolin Brombach for the initial MIBI mouse panel design, Vanessa Vogel for support with RNA extraction from FFPE sections, Danijela Heide for performing the TSA-based IF, and Lisa Künstler for her technical support. We want to acknowledge the excellent services provided by the Center for Preclinical Research, Light Microscopy, and Next Generation Sequencing Core Facilities of the DKFZ (Heidelberg, Germany).

Declaration of generative AI and AI-assisted technologies in the manuscript preparation process

During the preparation of this work, the authors used Anthropic Claude and ChatGPT for copy editing to check grammar and spelling and improve language and readability. After using this tool, the authors reviewed and edited the content as needed and take full responsibility for the content of the publication.

Supplementary data

Supplementary data to this article can be found online at https://doi.org/10.1016/j.jhepr.2026.101964.

References

Author names in bold designate shared co-first authorship.

[1] **Qurashi M**, **Vithayathil M**, Khan SA. Epidemiology of cholangiocarcinoma. Eur J Surg Oncol 2025;51:107064.
[2] Banales JM, Marin JJG, Lamarca A, et al. Cholangiocarcinoma 2020: the next horizon in mechanisms and management. Nat Rev Gastroenterol Hepatol 2020;17:557–588.
[3] Tomczak A, Springfeld C, Dill MT, et al. Precision oncology for intrahepatic cholangiocarcinoma in clinical practice. Br J Cancer 2022;127:1701–1708.
[4] Wu YM, Su F, Kalyana-Sundaram S, et al. Identification of targetable FGFR gene fusions in diverse cancers. Cancer Discov 2013;3:636–647.
[5] **Kendre G**, **Murugesan K**, Brummer T, et al. Charting co-mutation patterns associated with actionable drivers in intrahepatic cholangiocarcinoma. J Hepatol 2023;78:614–626.
[6] Silverman IM, Hollebecque A, Friboulet L, et al. Clinicogenomic analysis of FGFR2-rearranged cholangiocarcinoma identifies correlates of response and mechanisms of resistance to pemigatinib. Cancer Discov 2021;11:326–339.
[7] **Goyal L**, **DiToro D**, Hollebecque A, et al. Genomic correlates of response and resistance to the irreversible FGFR1-4 inhibitor futibatinib based on biopsy and circulating tumor DNA profiling. Ann Oncol 2025;36:414–425.
[8] Goyal L, Kongpetch S, Crolley VE, et al. Targeting FGFR inhibition in cholangiocarcinoma. Cancer Treat Rev 2021;95:102170.
[9] Lei JH, Lee M, Miao K, et al. Activation of FGFR2 signaling suppresses BRCA1 and drives triple-negative mammary tumorigenesis that is sensitive to immunotherapy. Adv Sci 2021;8:2100974.
[10] **Banales JM**, **Rodrigues PM**, Affò S, et al. Cholangiocarcinoma 2026: status quo, unmet needs and priorities. Nat Rev Gastroenterol Hepatol 2026;23:65–96.
[11] Abou-Alfa GK, Sahai V, Hollebecque A, et al. Pemigatinib for previously treated, locally advanced or metastatic cholangiocarcinoma: a multicentre, open-label, phase 2 study. Lancet Oncol 2020;21:671–684.
[12] Goyal L, Meric-Bernstam F, Hollebecque A, et al. Futibatinib for FGFR2-rearranged intrahepatic cholangiocarcinoma. N Engl J Med 2023;388:228–239.
[13] Lamarca A, Ostios L, McNamara MG, et al. Resistance mechanism to fibroblast growth factor receptor (FGFR) inhibitors in cholangiocarcinoma. Cancer Treat Rev 2023;121:102627.
[14] Galassi C, Chan TA, Vitale I, et al. The hallmarks of cancer immune evasion. Cancer Cell 2024;42:1825–1863.
[15] **Lidsky ME**, **Wang Z**, Lu M, et al. Leveraging patient derived models of FGFR2 fusion positive intrahepatic cholangiocarcinoma to identify synergistic therapies. NPJ Precis Oncol 2022;6:75.
[16] **Subbiah V**, **Sahai V**, Maglic D, et al. RLY-4008, the first highly selective FGFR2 inhibitor with activity across FGFR2 alterations and resistance mutations. Cancer Discov 2023;13:2012–2031.
[17] Zhen Y, Liu K, Shi L, et al. FGFR inhibition blocks NF-B-dependent glucose metabolism and confers metabolic vulnerabilities in cholangiocarcinoma. Nat Commun 2024;15:3805.
[18] Cristinziano G, Porru M, Lamberti D, et al. FGFR2 fusion proteins drive oncogenic transformation of mouse liver organoids towards cholangiocarcinoma. J Hepatol 2021;75:351–362.
[19] Kendre G, Marhenke S, Lorz G, et al. The co-mutational spectrum determines the therapeutic response in murine FGFR2 fusion-driven cholangiocarcinoma. Hepatology 2021;74:1357–1370.
[20] **Ochiai M**, **Yoshihara Y**, **Maru Y**, et al. Kras-driven heterotopic tumor development from hepatobiliary organoids. Carcinogenesis 2019;40:1142–1152.
[21] **Pepe-Mooney BJ**, **Dill MT**, Alemany A, et al. Single-cell analysis of the liver epithelium reveals dynamic heterogeneity and an essential role for YAP in homeostasis and regeneration. Cell Stem Cell 2019;25:23–38.e8.
[22] Liu CS, Wu YL, Komkova D, et al. Cell-of-origin, not oncogenic effect, determines desmoplastic immune exclusion in KRAS-driven liver cancer. bioRxiv 2026. in press.
[23] Baretti M, Shekhar S, Sahai V, et al. Deep immune profiling of intrahepatic cholangiocarcinoma with CODEX multiplexed imaging. Hepatol Commun 2025;9:e0632.
[24] Liu S, Weng J, Cao M, et al. FGFR2 fusion/rearrangement is associated with favorable prognosis and immunoactivation in patients with intrahepatic cholangiocarcinoma. Oncologist 2024;29:e1734–e1747.
[25] **Wu Q**, **Zhen Y**, Shi L, et al. EGFR inhibition potentiates FGFR inhibitor therapy and overcomes resistance in FGFR2 fusion–positive cholangiocarcinoma. Cancer Discov 2022;12:1378–1395.
[26] Spahn S, Kleinhenz F, Shevchenko E, et al. The molecular interaction pattern of lenvatinib enables inhibition of wild-type or kinase-mutated FGFR2-driven cholangiocarcinoma. Nat Commun 2024;15:1287.
[27] Nagarsheth N, Wicha MS, Zou W. Chemokines in the cancer microenvironment and their relevance in cancer immunotherapy. Nat Rev Immunol 2017;17:559–572.
[28] Calvisi DF, Boulter L, Vaquero J, et al. Criteria for preclinical models of cholangiocarcinoma: scientific and medical relevance. Nat Rev Gastroenterol Hepatol 2023;20:462–480.
[29] Artegiani B, Voorthuijsen L van, Lindeboom RGH, et al. Probing the tumor suppressor function of BAP1 in CRISPR-engineered human liver organoids. Cell Stem Cell 2019;24:927–943.e6.
[30] Nakanuma Y, Sato Y, Harada K, et al. Pathological classification of intrahepatic cholangiocarcinoma based on a new concept. World J Hepatol 2010;2:419–427.
[31] Alvaro D, Gores GJ, Walicki J, et al. EASL-ILCA Clinical Practice Guidelines on the management of intrahepatic cholangiocarcinoma. J Hepatol 2023;79:181–208.
[32] **Saha SK**, **Parachoniak CA**, Ghanta KS, et al. Mutant IDH inhibits HNF-4α to block hepatocyte differentiation and promote biliary cancer. Nature 2014;513:110–114.
[33] White BS, Sindiri S, Hill V, et al. Specific recognition of an FGFR2 fusion by tumor infiltrating lymphocytes from a patient with metastatic cholangiocarcinoma. J Immunother Cancer 2023;11:e006303.
[34] **Martin-Serrano MA**, **Kepecs B**, Torres-Martin M, et al. Novel microenvironment-based classification of intrahepatic cholangiocarcinoma with therapeutic implications. Gut 2023;72:736–748.
[35] Molina-Arcas M, Downward J. Exploiting the therapeutic implications of KRAS inhibition on tumor immunity. Cancer Cell 2024;42:338–357.
[36] Hamarsheh S, Groß O, Brummer T, et al. Immune modulatory effects of oncogenic KRAS in cancer. Nat Commun 2020;11:5439.
[37] Wu Q, Ellis H, Siravegna G, et al. Landscape of clinical resistance mechanisms to FGFR inhibitors in FGFR2-altered cholangiocarcinoma. Clin Cancer Res 2023;30:198–208.

[38] Cetintas VB, Batada NN. Is there a causal link between PTEN deficient tumors and immunosuppressive tumor microenvironment? J Transl Med 2020;18:45.
[39] **Bergholz JS**, **Wang Q**, Wang Q, et al. PI3Kβ controls immune evasion in PTEN-deficient breast tumours. Nature 2023;617:139–146.
[40] Peng W, Chen JQ, Liu C, et al. Loss of PTEN promotes resistance to T cell–mediated immunotherapy. Cancer Discov 2016;6:202–216.
[41] **Lin YX**, **Wang Y**, Ding J, et al. Reactivation of the tumor suppressor PTEN by mRNA nanoparticles enhances antitumor immunity in preclinical models. Sci Transl Med 2021;13. eaba9772.
[42] Adachi Y, Kamiyama H, Ichikawa K, et al. Inhibition of FGFR reactivates IFNγ signaling in tumor cells to enhance the combined antitumor activity of lenvatinib with anti-PD-1 antibodies. Cancer Res 2021;82. canres.2426.2020.
[43] Luster AD, Ravetch JV. Biochemical characterization of a gamma interferon-inducible cytokine (IP-10). J Exp Med 1987;166:1084–1097.

Keywords: Liver cancer; Tumor microenvironment; Immune escape; FGFR2 fusion; Kras mutation; Organoids; Cytotoxic T cells; Neutrophil granulocytes.

Received 10 October 2025; received in revised form 2 July 2026; accepted 14 July 2026; Available online 21 July 2026

Journal of Hepatology, Volume *8*

Supplemental information

FGFR2 fusion-driven cholangiocarcinoma is characterized by a distinct neutrophil-enriched tumor microenvironment in a syngeneic murine model

Nugzar Lekiashvili, Trinh Kieu Dinh, Alexander Olkus, Yu-Le Wu, Chun-Shan Liu, Daria Komkova, Albrecht Stenzinger, Felix J. Hartmann, Thomas Longerich, and Michael T. Dill

FGFR2 fusion-driven cholangiocarcinoma is characterized by a distinct neutrophil-enriched tumor microenvironment in a syngeneic murine model

Nugzar Lekiashvili, Trinh Kieu Dinh, Alexander Olkus, Yu-Le Wu, Chun-Shan Liu, Daria Komkova, Albrecht Stenzinger, Felix J. Hartmann, Thomas Longerich, Michael T. Dill

Table of contents

Supplementary Materials and Methods……………………………..………..…………2
Supplementary References. ……………………………………………………………18
Supplementary Figures………………………………………………..………..………..21
Table S1. ……………………………………………………………………………….31
Table S2.……..……..……………………………………………………………………33
Tables S3-5…………………………………………………......…separate excel files

Supplementary Materials and Methods

Organoid media generation and composition

Organoid media consisted of a 1:1 mixture of L-WRN conditioned media generated as described previously,[1] and advanced DMEM/F12 (Thermo Fisher Scientific, #12634010) supplemented with 1% (vol/vol) Glutamax (ThermoFisher Scientific, #35050038), 10 mM HEPES (ThermoFisher Scientific, #35050038), 1:50 B27 supplement (without vitamin A) (ThermoFisher Scientific, #12587010), 1:100 N2 supplement (ThermoFisher Scientific, #17502048), 10 mM Nicotinamide (Sigma-Aldrich, #N0636), 50 ng/ml rmEGF (ThermoFisher Scientific, #31509), 50 ng/ml rhHGF (ThermoFisher Scientific, #10039-500), 1% (vol/vol) penicillin/streptomycin (ThermoFisher Scientific, #15140122), and 100 µg/ml Primocin (Invivogen, #ant-pm1) and - upon initial derivation and upon passage - 10 µM Y27632 (Holzel, #TMO-T1725-50mg).

Murine cholangiocyte isolation and organoid culture

Wild-type cholangiocytes were isolated from 4-6 week-old C57BL/6J mice, adapting a published protocol.[2] In short, after euthanization via cervical dislocation, a midline laparotomy was performed and mouse livers immediately perfused via portal vein cannulation by a pre-warmed (37°C) perfusion buffer (50 mM EDTA, 10 mM HEPES in 1X HBSS [GIBCO]) at 6 mL/min for 2 min, then 8 mL/min for another 2 min, followed by collagenase IV buffer (Collagenase IV:150 U/mL, Invitrogen, #17104019; in 1X HBSS with 1.25 mM $CaCl_2$, 4 mM $MgCl_2$, 10 mM HEPES) for 5–10 min at 8 mL/min. After removal of the gallbladder, the liver capsule was removed and the tissue gently agitated to remove hepatocytes. The remaining liver scaffold underwent sequential digestions with collagenase IV buffer (150 U/mL), accutase, and 0.25% trypsin for 30, 30, and 20 min respectively at 37°C. Cells were collected after each step, filtered (100 µm), washed, and resuspended in ice-cold resuspension buffer (2% FBS, 1.25 mM $CaCl_2$, 4 mM $MgCl_2$, 10 mM HEPES, 5 mM glucose in 1X HBSS). The suspensions were then combined and spun at 50 G for 5 min at 4°C. The supernatant was then transferred and spun at 300 G for 5 min at 4°C. The cell pellet was then resuspended in

basement membrane extract (BME, Cultrex Reduced Growth Factor Basement Membrane Extract, Type 2, R&D Systems, #3533-010-02) and plated in 24-well plates (50 µl droplet per well). After solidification, BME droplets were overlaid with 500 µl of organoid media.

After 7-14 days, outgrowing cholangiocyte organoid clones were picked for further passage. For expansion and passaging, organoids were mechanically disrupted with a 25G needle on a 1ml syringe, the cell-suspension supplemented with fresh ice-cold DMEM and spun at 300 G for 5 min at 4°C. The cell pellet was resuspended in fresh BME. Media was changed two to three times per week. Bright-field images of organoids were taken with a DM IL LED Inverted Laboratory Microscope (Leica).

Cloning of sgRNA constructs

For single guide (sgRNA) constructs, the pX458 pSpCas9(BB)-2A-GFP and pX459 pSpCAs9(BB)-2A-Puro V2.0 backbone plasmids were used (Addgene, #48138 and #62988).[3] The pX458-sgRNA(*Trp53*) plasmid was constructed by integrating the published *Trp53* sgRNA oligos into the pX458 vector as described.[4] The sgRNA(*Kras*)-*KrasG12D*_HDR plasmid was generated by modifying the parental plasmid AAV-KPL (Addgene #60224).[4] Briefly, AAV-KPL was digested with BamHI and KpnI to excise the additional sgRNA cassette. The linearized backbone was blunt-ended by Klenow and subsequently ligated to re-circularize the plasmid. Additional sgRNA were designed using Benchling (Biology Software, 2022) and the SYNTHEGO CRISPR design tool (https://design.synthego.com) and oligos cloned into the pX458 or pX459 vector. All oligo sequences are listed in **Table S1**.

Genetic engineering of monoclonal organoid lines

For transfection, organoids were dissociated by incubating with 500 µL/well of Cell Recovery Solution (Corning. #354253) on ice for 20 min, followed by centrifugation at 500 G for 5 min at 4°C. The cell pellets were resuspended in TrypLE (Thermo Fisher Scientific, #12605010) at RT for 5-8 min with intermittent shaking, then

mechanically dissociated with a 25G needle. After quenching the digestion reaction with ice-cold DMEM and centrifugation, cells were resuspended in organoid medium with 10µM of Y-27632 (TargetMol, cat#TMO-T1725-50mg) and plated at 450 µL/well in a 48-well plate. For transfection, Lipofectamine 2000 (Invitrogen, cat# 11668019) mixed with plasmid DNA (1.5 µg/reaction) was prepared according to manufacturer's instruction and added dropwise to the cells. Cell plates were centrifuged at 600 G for 60 min at 32°C, then incubated at 37°C for 4 hours. Cells were collected, centrifuged (845 G, 5 min, 4°C), resuspended in BME, and seeded in 50 µl droplets into a 24-well plate. Organoid media was added after solidification of the BME.

To generate cholangiocyte organoids carrying *Trp53* deletion, wildtype organoids were transfected with pX458-sgRNA(*Trp53*) plasmid and treated with 7.5 µM Nutlin-3 (Biotrend #A4228-S) starting 2 days post-transfection for functional selection. Outgrowing clones were picked between 14-20 days after transfection. To generate cholangiocyte organoids carrying a *Kras*^{G12D} mutation, cells were co-transfected with pX459-(BB) and sgRNA(*Kras*)-*Kras*^{G12D}_HDR plasmids. The day after transfection, 1 µM of Alt-R™ HDR Enhancer V2 (IDT, #10007921) was added for 24 hours to promote homology-directed repair. On day 3, cells were treated with 1 µg/mL puromycin (Sigma-Aldrich, # P9620-10ML) in organoid media for 2 days to select for transfected cells. Beginning 3 days post-transfection, cells were switched to organoid media lacking mEGF and hHGF supplemented with 1 µM gefitinib (Biotrend, #A8219-S) for functional selection. Outgrowing clones were picked approximately 14 days after transfection.

The generation of *Fgfr2* rearrangements was done in a similar fashion with co-transfection of organoids with pX459-sgRNA(*Fgfr2*) and either pX459-sgRNA(*Ahcyl1*), pX459-sgRNA(*Bicc1*), or pX459-sgRNA (*Tacc3*) template, with selection with puromycin and functional selection under growth factor withdrawal as described for *Kras*^{G12D}. Outgrowing clones were picked approximately 5-7 days after transfection (**Fig. S1**). *Pten-*, *Ckdn2a/b-*, *Bap1-* and *Pbrm1*-deleted organoids were generated by transfection with pX459 plasmid with the respective sgRNA,

treated with 1 µg/mL puromycin in organoid media on day 3-4 and outgrowing organoids picked to generate monoclonal lines and screened for deletion.

To confirm successful genome editing of the picked organoid clones, genomic DNA was isolated with Quick-DNA Miniprep Kit (Zymo Research) and the targeted genomic region amplified by a standard PCR protocol using 2X Taq Master Mix (Genaxxon, #3029) and the primers indicated in **Table S1**. PCR products were verified by Sanger sequencing (Microsynth AG, Balgach, Switzerland). Indel mutation frequencies were analyzed using EditCo's ICE Analysis tool (https://ice.editco.bio/#).[5] For *Kras*^{G12D} mutation in chol-PK, PCR amplicons were further confirmed by TA-cloning (PCR Cloning, NEB, cat#E1203S) and Sanger sequenced.

Lentivirus production and transduction

The Lenti-luciferase-P2A-Neo plasmid (Addgene #105621) was co-transfected with packaging plasmid psPAX2 and pMD2.G (PlasmidFactory GmbH & Co. KG, Bielefeld, Germany) into HEK293T cells by TransIT LT1 transfection reagent (Mirus Bio, Madison, WI, USA) according to the manufacturer's instructions. The supernatant containing lentivirus was harvested 2 days after transfection, filtered, and stored at -80°C until usage. Organoids were infected with the lentivirus supernatant and selected by 500 µg/ml G418 (ThermoFisher Scientific, #10131027) for at least 2 weeks.

RNA extraction and reverse transcription quantitative PCR

RNA from cells or tumors was isolated with Direct-zol RNA Microprep kit (Zymo Research, #R2062) with on-column DNase I digestion according to manufacturer's instructions. RNA concentration was measured using NanoDrop (Thermo Fisher Scientific, Waltham, MA, USA).

RNA extraction from FFPE sections was performed as previously described.[6] Briefly, tumor areas were marked on H&E-stained slides and corresponding tissue areas were macrodissected, marking the tumor area and scratching material from subsequent unstained 5 µm thick sections. RNA extraction was conducted automatically using the Maxwell RSC FFPE RNA Kit on

a Maxwell RSC Benchtop Instrument (Promega, Madison, WI, USA), in accordance with the manufacturer's instructions. The RNA concentration was measured fluorometrically (QuBit 2.0 RNA high sensitivity kit, Thermo Fisher Scientific, Waltham, MA, USA) following the manufacturer's instructions.

Reverse transcription was carried out using iScript cDNA Synthesis kit (Bio-Rad, # 1708891) with 500ng or 1µg RNA as template and cDNA amplified using 2X Taq Master Mix (Genaxxon, #3029) with target region-specific primers (**Table S1**). Quantitative polymerase chain reaction (qPCR) was performed with 384-well or 96-well plates using 10ng cDNA input and Luna Universal qPCR Master Mix SYBR Green (New England Biolabs, #M3003E) with gene specific custom made primers (Sigma Aldrich) (**Table S1**) on a Quant Studio 6Pro Cycler (Therma Fisher Scientific, Waltham, MA, USA) or a CFX-Maestro (BIO-RAD, Feldkirchen Germany), respectively.

Initial denaturation was performed for 10s at 95°C. Amplification was performed in a two-step cycle for 15s at 95°C followed by 1 min at 60°C. Primers were evaluated by analysis of melting curves and amplification size of the PCR products using 1.5 % agarose gel electrophoresis. The primer efficiency was tested by serial dilution. Relative mRNA expression was analysed using the ΔΔ-CT method by normalisation to the housekeeping genes (glyceraldehyd-3-phosphat-dehydrogenase (GAPDH) or ribosomal protein 14 (RPS14).

Western blotting

Organoids and 2D cells were starved for 48 hours in basal media without supplements before protein extraction. For lysis, 4–5 confluent 50 µl BME domes were processed using Cell Recovery Solution (Corning #354253) and ice-cold DMEM. Cell pellets were lysed in RIPA buffer (ThermoFisher Scientific, #89900) supplemented with phosphatase inhibitor (ThermoFisher Scientific, #89900) and protease inhibitors (Hölzel Diagnostika, # HY-K0011-25pcs), incubated on ice for 30 min, and centrifuged at 16'000 G for 20 min at 4°C. Protein concentrations were determined using the BCA assay (ThermoFisher Scientific, #23225). 20 µg of protein were mixed with Laemmli buffer (Alfa Aesar, #J61337-AC), boiled, and

separated by SDS-PAGE using 7.5% TGX™ gels (Bio-Rad, #1610180). Proteins were transferred to 0.45 µm PVDF membranes (Millipore, # IPVH00010) overnight at 10 mA in cold conditions. Membranes were blocked in 3% BSA (Sigma-Aldrich, # A7030-100G) and incubated with primary antibodies overnight at 4°C (**Table S2**). After washing, HRP-conjugated secondary antibodies were applied, and chemiluminescent signals (Millipore, WBKLS0500) were developed using an Amersham Imager 680. For reprobing, membranes were stripped in glycine-based stripping buffer (3% glycine, 0.2% SDS and 2% Tween 20 at pH 2.2) and re-blocked.

Organoid viability assay

Organoids were mechanically and enzymatically dissociated as described above and seeded 1200 cells in 10 µl BME per well in white 96-well microplates. After BME solidification, 100 µl of organoid media without mEGF and hHGF was added. Media was renewed every 3rd day. Cell viability was assessed at the indicated time points using CellTiter-Glo® 3D Cell Viability Assay (Promega, #G9683), according to the manufacturer's instructions. Growth curves are presented as mean of 4 biological replicates, and each biological replicate contains 2 technical replicates.

Animal experiments

C57BL/6J mice were obtained from Janvier Labs and NOD.Cg-*Prkdc^{scid} Il2rg^{tm1Wjl}* (NOD scid gamma, NSG) internally from the German Cancer Research Center (DKFZ). All mice were housed under specific pathogen-free (SPF) conditions in individually ventilated cages on a 12-hour light/dark cycle at 21–23 °C, with ad libitum access to food and water. All experiments were conducted in accordance with German law and approved by the Regierungspräsidium Karlsruhe (approval no. G34/22). For orthotopic tumorigenicity assays, mice older than 8 weeks were used. Mice were anesthetized with isoflurane (0.5-1% with oxygen), a small midline laparotomy and intrahepatic implantation via 28-gauge syringe into the left liver lobe was performed. The injection site was then compressed with a cotton-top to avoid leakage or bleeding, and peritoneum and skin closed with absorbable suture

(Ethicon, VCP303H) and 9 mm clips (Fine Science Tools, 12022-09), respectively. Proper peri- and postoperative analgesia was applied with metamizole and lidocaine. For implantation, typically 5-10 × 10^5 cells in 25 µl DMEM:BME mixture (1:1) were used**.**

The *in vivo* imaging System (IVIS) was used for tumor growth surveillance. To this end, the abdominal area of the mice was shaved and 150 mg/kg D-Luciferin (Promega, E1605) injected intraperitoneally, and imaging performed under anesthesia with isoflurane (1–2 vol.% in oxygen).

For the *in vivo* treatment experiments of total 2 weeks, pemigatinib (Biosynth, #NKC85777) dissolved in 1% Tween-80 (Sigma-Aldrich, P1754) was administered at 3 mg/kg body weight per oral gavage once daily 5 out of 7 days for a total of 2 weeks to PFfPt tumor-bearing C57BL/6J mice.

Human CCA study population

For the histological analysis of human FGFR2 fusion-driven and other control iCCA, patients were identified from the Liver Cancer Center Heidelberg (LCCH) registry, where data on molecular alterations is typically available for patients undergoing palliative systemic therapy. Samples were included only from treatment-naïve patients and where enough FFPE block material containing tumor tissue was available for sectioning.

Written informed consent was obtained from all patients prospectively enrolled in the LCCH registry, which was approved by the Heidelberg University ethics committee (ethical permit number S-693/2019), complied with the provisions of the Good Clinical Practice guidelines and the Declaration of Helsinki and local laws and fulfilled the Regulation (EU) 2016/679 of the European Parliament and of the Council of 27 April 2016 on the protection of natural persons with regard to the processing of personal data (ID number DSAN854-A-OS/5).

Histopathology and immunostaining

Tissue specimens were fixed in 4% paraformaldehyde or 10% formalin solution over night at room temperature, washed with PBS and 70% ethanol, and

embedded in paraffin. Serial sections of 3-5 µm thickness were generated and rehydrated. Hematoxylin and eosin and periodic acid-Schiff (PAS) staining was performed following routine histological protocols. For immunohistochemical analyses, sections were treated with citric-acid based pH 6.0 Antigen Unmasking Solution (Biozol, H-3300) in a steamer for 20 min. Slides were then treated with 0.3% hydrogen peroxide to quench endogenous peroxidase activity. To prevent non-specific antibody binding, slides were incubated in protein blocking solution (5% donkey Serum [Biozol, LIN-END9010] in 1% BSA/PBS/0.1% Tween 20) for 1 hour before overnight incubation with primary antibody at 4°C (**Table S2**). After washing, appropriate biotinylated secondary antibodies (Vector Labs) were used with the Vectastain Elite ABC-HRP reagent (Vector Labs, PK-7100) and Diaminobenzidine-reaction kit (Vector Labs, SK-4100) for visualisation of bound antibodies. Harris modified hematoxylin (Carl Roth, T864.1) was used to counterstain nuclei. Finally, slides were washed, dehydrated, and mounted using Vectamount (Vector Labs, H-5700-60).

Stained sections were scanned on a Zeiss Axio Scan.Z1 slide scanner at the DKFZ Light Microscopy Facility. Tumors were assessed qualitatively by an expert liver pathologist in a blinded fashion.

For immunofluorescence stains, 2-3 µm thick FFPE serial sections were cut and deparaffinized and rehydrated. For human tissue sections, antigen retrieval was performed using the Epredia™ Dewax and HIER Buffer H (#TA999DHBH) and slides were boiled at 102°C for 1h. For murine tissue sections antigen retrieval was performed with EDTA based buffer (Leica, #AR9640) for 30min at 100°C or with a TRIS based buffer for 20 min in a steamer. Slides were washed and blocked with 5% donkey Serum (Biozol, LIN-END9010) in 1% BSA/PBS/0.1% Tween-20 for 30min. Afterwards cells were incubated with primary antibodies (**Table S2**) in blocking solution overnight at 4°C. After washing, slides were incubated with appropriate diluted fluorophore-conjugated secondary antibodies for 1h at RT, and then after washing, incubated with DAPI (1:100) for 5 min and mounted with of Prolong Gold antifade with DAPI.

Tyramide signal amplification (TSA) was performed using the Bond Polymer Refine Detection Kit (Leica: DS9800) and reagents FP1487001KT Opal 520 Reagent (FITC), FP1495001KT Opal 620 Reagent (Texas red), and FP1488001KT Opal 570 Reagent (Cy3) (Akoya) with antibodies and dilutions listed in **Table S2**. For PD-1 stainings a secondary antibody incubation was performed using a rabbit anti-goat antibody (Dako, P04990) before Opal reagents were added. Stained sections were scanned using Nano Zoomer S60 (Hamamatsu)

Tissue mounting and immunostaining for multiplexed ion beam imaging (MIBI)

Formalin-fixed and paraffin-embedded tissue blocks were sectioned at 5 µm and mounted onto gold-coated conductive slides (Ionpath, 567001). To minimize batch effects, all samples were processed together in parallel and stained with common antibody master mixes, following established protocols.[7] Briefly, sections were deparaffinized and rehydrated as for IHC preparation, then subjected to antigen retrieval. Non-specific binding was blocked for 1 h at room temperature, followed by M.O.M. reagent (Vector, MKB-2213-1) to prevent background from endogenous mouse IgG. Slides were then incubated overnight at 4 °C with a filtered primary antibody master mix for indirect staining. The following day, sections were washed and incubated with a filtered secondary antibody master mix for 1 h at room temperature. After washing, slides were incubated overnight at 4 °C with a filtered direct-staining antibody master mix. All antibodies used for MIBI staining are listed in **Table S3**. Following a final wash, sections were fixed in 2% (v/v) glutaraldehyde for 5 min at room temperature and dehydrated through a graded ethanol series. Prior to acquisition, stained slides were stored in a vacuum cabinet to prevent metal oxidation and preserve tissue integrity.

Multiplexed imaging and data preprocessing

H&E images of serial sections corresponding to the MIBI slides were used to identify tumor boundary regions for imaging. For each sample, one region was manually selected in DUEfiner, a customized interactive image viewer implemented in Python, and subdivided into 400 × 400 µm fields of view (FOVs).

Each FOV was acquired at a dwell time of 0.5 ms using the “Coarse” preset, yielding a pixel size of 390 nm.

Images were extracted from mass spectrometry data using Toffy (https://github.com/angelolab/toffy). For each pixel, the intensity of a given mass m was obtained by integrating ion counts over the range $[m - 0.3, m]$. To correct for systematic intensity gradients across FOVs — analogous to illumination correction in light and fluorescence microscopy —, all images were examined for such patterns. A gradient was defined as a consistent intensity variation present across channels and multiple FOVs. For each channel, the gradient was estimated by summing Gaussian-blurred versions of its FOVs, thereby reducing contributions from biological signals. Gradient magnitude scaled with channel mass, and a per-channel scaling factor was derived by linear regression between a proxy of gradient magnitude and channel mass. To further suppress biological signal, a two-dimensional correction map was generated by summing the scaled gradients across all channels. Correction was applied by dividing each channel by this map, adjusted for the channel-specific gradient magnitude across all FOVs.

Gradient-corrected images were subsequently processed with Rosetta compensation to reduce cross-channel contamination, as previously described.[8] The mass and relative abundance (coefficient) of interfering species in the source reagent are well characterized,[9] enabling estimation of their contribution by scaling the source channel intensity before subtracting it from the target channel. A coefficient matrix for all source–target channel pairs is available at https://doi.org/10.1101/2025.01.26.634557.

To remove gold-signal-based background, bare slide regions were identified and excluded based on elevated gold (Au) ion signal from the conductive slide coating. Pixel intensities in the Au channel were normalized to the 99.9th percentile and arcsinh-transformed (cofactor = 100). The transformed intensity distribution was modeled with a double-Gaussian fit to distinguish gold-negative and gold-positive populations. The intersection of the two Gaussian components was defined as the threshold for gold-positive pixels. This threshold was then back-transformed into raw intensity space and applied to each FOV to generate binary masks of gold-

positive pixels. These masks were subsequently used to exclude bare slide regions from downstream analysis.

Image-based single cell data quantification and filtering

FOV tiles were stitched into whole-region images. Cells were segmented in the stitched images using the Cellpose TN model,[10] re-trained on unpublished MIBI datasets comprising 335 cells from human normal liver, normal lung, colorectal cancer, and lung cancer tissues. The model was applied with a cell diameter of 30 pixels, a flow threshold of 0.7, and a cell probability threshold of –5. Cell masks were filtered based on size, nuclear intensity, and gold-pixel content to retain only high-confidence single cells. Masks smaller than 100 pixels were removed as over-segmented. For retained cells, features including spatial location in the whole-region image, cell size, and cell-size–normalized (mean) channel intensities were calculated. Histone H3 (HH3) intensities were either summed or averaged per cell, percentile-scaled, arcsinh-transformed, and thresholded using the intersection of a double-Gaussian fit to identify nuclei-negative populations. Cells below this threshold were flagged as out-of-plane. Similarly, cells with >50% of their area overlapping bare-slide pixels were flagged as incomplete. All flagged cells were excluded from downstream analysis.

Data annotation and tissue zone identification

To generate robust and accurate cell labels, we employed an iterative clustering strategy with human-in-the-loop curation across multiple levels, defined by the hierarchy of lineage markers (**Figure 4**). At each level, FlowSOM [11] was used to cluster cells into self-organizing map (SOM) nodes based on lineage markers relevant for that level. Nodes were then merged to align with defined cell lineages according to marker positivity and visual inspection. Nodes with ambiguous marker profiles were re-clustered and annotated; cells that remained ambiguous were labeled as “other.” Importantly, at each level, cells belonging to different higher-level lineages were classified separately to maintain lineage-specific resolution.

At level 1, cells were broadly classified into hepatocytes, cancer, endothelial, CAFs, and immune cells. At level 2, CAFs were further classified according to Vimentin (Vim), αSMA, COL1a1, and PDGFRb positivity, while immune cells were subdivided into $CD3^+$, $CD20^+$, $CD11c^+$, $CD11b^+$, and $CD11b^+CD11c^+$ populations. At level 3, $CD3^+$ cells were further divided into $CD4^+$, $CD8^+$, and $CD4^+FOXP3^+$ subsets, and $CD11b^+$ cells into $F4/80^{med}$, $Ly6G^+$, and $F4/80^{hi}$ subsets. At level 4, $F4/80^{hi}$ macrophages were split into $CD163^-$ and $CD163^+$ populations. Immune cells with ambiguous finer lineage profiles were marked as "other immune." All annotation steps were performed using our custom viewer, UELer (https://github.com/HartmannLab/UELer).

PK tissues were divided into three zones: adjacent normal, tumor periphery, and tumor core. The tumor border was manually marked as the interface where hepatocytes confronted tumor cells, using tissue images with level 1 markers rendered in UELer. Relative to this border, the hepatocyte side was defined as adjacent normal, while the opposite side was defined as tumor. The tumor region was further subdivided into periphery and core based on distance from the border, using a cutoff of 150 µm.

MIBI statistical analysis

Cell densities were calculated as the counts of a specified cell type divided by the total area of all cells within a tumor. Cell densities between groups were compared via linear mixed-effects model with tumor type as a fixed effect and animal as a random intercept to account for multiple nodules per animal and a significance threshold of $p < 0.05$. Marker abundance between groups was compared via linear mixed-effects model with tumor type as a fixed effect, animal as a random intercept, and tumor nodules nested within animal as an additional random intercept to account for multiple cells per tumor per animal, with a significance threshold of $p < 0.05$. The proportion of Ki67-positive and Vimentin-positive cells within each cell population was determined by applying a predefined intensity threshold to classify individual cells as positive or negative, with per-tumor percentages calculated as the number of positive cells divided by the total cell

count. The positivity of Ki67 and Vimentin between groups was compared via binomial generalized linear mixed-effects model with group (PFfPt vs. PK) as a fixed effect and animal as a random intercept to account for multiple nodules per animal. All significance values were determined from permutation-based p-values corrected for multiple testing using the false discovery rate (FDR) method, applied across comparisons within each data type (i.e., cell density, Ki67 positivity, Vimentin positivity, and marker abundance separately).

Spatial Interaction Analysis

To characterize cell-type co-localization patterns in the tumor microenvironment, we performed spatial interaction analysis using the Conditional Z-score Interaction (COZI) method implemented within the SCIMAP framework (https://github.com/SchapiroLabor/scimap_COZI/tree/master).[12] Prior to analysis, hepatocytes, uninformative cell populations ("other immune cell," "other"), and in the PK samples, cells residing in the core zone were excluded. For each condition, spatial interactions were computed using a K-nearest-neighbor graph ($K = 5$) with 300 permutations per tumor nodule, applying conditional normalization and Z-score-based significance estimation. For each directed cell-type pair (source → target), Z-scores were summarized at the per-condition level by taking the median across tumor nodules, and permutation-based *p*-values were averaged accordingly. Cell-type pairs with a mean p-value ≥ 0.05 were considered non-significant and excluded from the interaction network.

FGFR inhibitor treatment in vitro

For the colony forming assay, PK and PFf organoids were first grown as a monolayer on the cell culture dish for several passages with media transition to DMEM with 10% FBS and 1% Penicillin/Streptomycin(P/S). Cells below passage 20 were used during the experiments and were seeded in 6-well plates at 500 cells/well for PK and 1000 cells/well for PFf with 2 ml of DMEM with 10% FBS. On the following day, cells were treated with the indicated concentrations of pemigatinib (MedChemExpress, #HY-109099), futibatinib (MedChemExpress,

#HY-100818) or vehicle (DMSO). Maximal DMSO concentration used was 560 nM. Media was renewed every 3 days. After 15 days for PK and 22 days for PFf, colonies were fixed with 70% ethanol for 10 minutes at room temperature and stained with 1% (w/v) crystal violet in distilled water for 10 minutes. Excess dye was removed by rinsing plates with tap water, followed by air-drying overnight. For quantification, crystal violet was extracted using 1 mL of 10% acetic acid (Thermo Fisher, #A38212) on a shaker for 1 hour at room temperature. The extracts were diluted 1:4 and absorbance was measured at 600 nm using a GloMax Explorer System (Promega). Absorbance from blank wells (10% acetic acid only) was subtracted.

For organoid growth inhibition, PK and PFf organoids were seeded as 2000 cells in 20 µl BME per well in white 96-well microplates. After BME solidification, 100 µl of organoid media without mEGF and hHGF was added. The next day cells were treated with the 400nM of pemigatinib (MedChemExpress, #HY-109099), 400nM futibatinib (MedChemExpress, #HY-100818) or vehicle (DMSO 0.00004 %). Media was renewed every 3rd day. Cell viability was assessed at the indicated time points using CellTiter-Glo® 3D Cell Viability Assay (Promega, #G9683), according to the manufacturer's instructions.

RNA sequencing and integrative data analysis

RNA was isolated using the RNeasy Plus Mini kit (Qiagen, 74134), and RNA integrity was analyzed by a 2100 Bioanalyzer using the RNA 600 Nano Kit (Agilent technologies) according to the manufacturer's protocol. Sequencing libraries were prepared by the Genomics and Proteomics Core Facility (DKFZ, Heidelberg, Germany) from total RNA using the Illumina TruSeq Stranded Total RNA Library Prep Kit according to the manufacturer's instructions. For sequencing, multiplexed libraries were sequenced in a paired-end setting (100 bp) on an Illumina NovaSeq 6000 sequencer.

Data was analyzed with the DKFZ Omics IT and Data Management Core Facility(ODCF) RNAseq workflow (version 4.0.0). Reads were aligned by 2-pass alignment to the mouse genome mm10 with PhiX decoy sequence and GENCODE

mouse vM12 annotation using the STAR aligner version 2.5.3a. The raw counts were obtained using the featureCounts tool from Subread (v1.6.5).[13]

Downstream analyses were performed in R 4.4.3. Differential expression analysis was conducted with DESeq2 v1.46.0.[14] Genes were retained if they had at least 10 counts in at least three samples. The DESeq2 model used the design formula `~ line * drug`, with PK and DMSO set as the reference levels. Genes with Benjamini-Hochberg false-discovery-rate adjusted *p* values < 0.05 and absolute log2 fold changes > 1 were considered differentially expressed. Heatmaps were generated from row-scaled, variance-stabilized expression values for the top protein-coding genes ranked by the absolute DESeq2 Wald statistic.

Gene set enrichment analysis was performed using genes ranked by the DESeq2 Wald statistic.[15] MSigDB mouse Hallmark gene sets,[16] were tested with the fgsea R package (v1.32.4) Click or tap here to enter text.GO Biological Process enrichment[17] was performed with clusterProfiler v4.14.6[18,19] using org.Mm.eg.db Ensembl annotations. Redundant GO terms were simplified separately for positively and negatively enriched terms using semantic similarity based on the Wang method.[20] Pathways with FDR-adjusted p values < 0.05 were considered significant, and normalized enrichment scores and adjusted p values were reported.

Statistical Analysis/Software

Data were analyzed and plotted using QuPath (V0.7), Living Image (IVIS) and GraphPad Prism 11 (GraphPad). For quantitative analyses of immunostains, the whole scanned images were loaded into QuPath.[21] Within QuPath, annotations were created to separate a tumor area from the surrounding liver tissue. Automated cell detection and classification tools were used to identify and quantify positive cells within the defined tumor area and were manually checked and corrected where necessary. Ki67 positivity was evaluated using high-power fields (HPFs). For each tissue section, 5–6 HPFs were randomly generated. Quantitative analysis was performed using QuPath software with trained object classifiers.

For the analysis of human tissue stains, random 10 HPFs of $1mm^2$ were created using the automated region classifier.
Technical and biological replicates and respective statistical test used are specified for each experiment in the figure legends. Figure panels were generated by Affinity (Canva). For the creations of illustration panels in Figure 1A, 1C, 5A and 7A icons from BioRender.com were used.

Supplementary References

[1] Miyoshi H, Stappenbeck TS. In vitro expansion and genetic modification of gastrointestinal stem cells in spheroid culture. Nature Protocols 2013;8:2471–82. https://doi.org/10.1038/nprot.2013.153.

[2] **Pepe-Mooney BJ**, **Dill MT**, Alemany A, et al. Single-Cell Analysis of the Liver Epithelium Reveals Dynamic Heterogeneity and an Essential Role for YAP in Homeostasis and Regeneration. Cell Stem Cell 2019;25:23-38.e8. https://doi.org/10.1016/j.stem.2019.04.004.

[3] **Ran FA**, **Hsu PD**, Wright J, et al. Genome engineering using the CRISPR-Cas9 system. Nature Protocols 2013;8:2281–308. https://doi.org/10.1038/nprot.2013.143.

[4] **Platt RJ**, **Chen S**, Zhou Y, et al. CRISPR-Cas9 Knockin Mice for Genome Editing and Cancer Modeling. Cell 2014;159:440–55. https://doi.org/10.1016/j.cell.2014.09.014.

[5] Conant D, Hsiau T, Rossi N, et al. Inference of CRISPR Edits from Sanger Trace Data. The CRISPR Journal 2022;5:123–30. https://doi.org/10.1089/crispr.2021.0113.

[6] Ball M, Beck S, Wlochowitz D, et al. Diagnostic whole transcriptome sequencing in a series of 1233 FFPE solid tumor samples. Br J Cancer 2026;134:1101–10. https://doi.org/10.1038/s41416-025-03307-8.

[7] Bosse MM. MIBI staining v2 2022. https://doi.org/10.17504/protocols.io.dm6gprk2dvzp/v5.

[8] Vulliard L, Glauner T, Truxa S, et al. Robust multicellular programs dissect the complex tumor microenvironment and track disease progression in colorectal adenocarcinomas. 2025. arXiv:2510.05083. https://doi.org/10.48550/arXiv.2510.05083

[9] **Greenwald NF**, **Nederlof I**, Sowers C, et al. Temporal and spatial composition of the tumor microenvironment predicts response to immune checkpoint inhibition. bioRxiv 2025:2025.01.26.634557. https://doi.org/10.1101/2025.01.26.634557.

[10] Pachitariu M, Stringer C. Cellpose 2.0: how to train your own model. Nat Methods 2022;19:1634–41. https://doi.org/10.1038/s41592-022-01663-4.

[11] Gassen SV, Callebaut B, Helden MJV, et al. FlowSOM: Using self-organizing maps for visualization and interpretation of cytometry data. Cytometry Part A 2015;87:636–45. https://doi.org/10.1002/cyto.a.22625.

[12] Schiller C, Ibarra-Arellano MA, Bestak K, et al. Comparison and optimization of cellular neighbor preference methods for quantitative tissue analysis. Nat Commun 2026;17:3514. https://doi.org/10.1038/s41467-026-71699-z.

[13] Liao Y, Smyth GK, Shi W. featureCounts: an efficient general purpose program for assigning sequence reads to genomic features. Bioinformatics 2014;30:923–30. https://doi.org/10.1093/bioinformatics/btt656.

[14] Love MI, Huber W, Anders S. Moderated estimation of fold change and dispersion for RNA-seq data with DESeq2. Genome Biol 2014;15:550. https://doi.org/10.1186/s13059-014-0550-8.

[15] **Subramanian A**, **Tamayo P**, Mootha VK, et al. Gene set enrichment analysis: A knowledge-based approach for interpreting genome-wide expression profiles. Proc Natl Acad Sci 2005;102:15545–50. https://doi.org/10.1073/pnas.0506580102.

[16] Castanza AS, Recla JM, Eby D, et al. Extending support for mouse data in the Molecular Signatures Database (MSigDB). Nat Methods 2023;20:1619–20. https://doi.org/10.1038/s41592-023-02014-7.

[17] Aleksander SA, Balhoff J, Carbon S, et al. The Gene Ontology knowledgebase in 2023. GENETICS 2023;224:iyad031. https://doi.org/10.1093/genetics/iyad031.

[18] **Xu S**, **Hu E**, **Cai Y**, et al. Using clusterProfiler to characterize multiomics data. Nat Protoc 2024;19:3292–320. https://doi.org/10.1038/s41596-024-01020-z.

[19] Yu G, Wang L-G, Han Y, et al. clusterProfiler: an R Package for Comparing Biological Themes Among Gene Clusters. OMICS: A J Integr Biol 2012;16:284–7. https://doi.org/10.1089/omi.2011.0118.

[20] Wang JZ, Du Z, Payattakool R, et al. A new method to measure the semantic similarity of GO terms. Bioinformatics 2007;23:1274–81. https://doi.org/10.1093/bioinformatics/btm087.

[21] Bankhead P, Loughrey MB, Fernández JA, al. QuPath: Open source software for digital pathology image analysis. Sci Rep 2017;7:16878. https://doi.org/10.1038/s41598-017-17204-5.

[22] The Uniprot Consortium. UniProt: the Universal Protein Knowledgebase in 2025. Nucleic Acids Res 2024;53:D609–17. https://doi.org/10.1093/nar/gkae1010.

[23] **Sievers F**, **Wilm A**, Dineen D, et al. Fast, scalable generation of high-quality protein multiple sequence alignments using Clustal Omega. Mol Syst Biol 2011;7:MSB201175. https://doi.org/10.1038/msb.2011.75.

Fig. S1

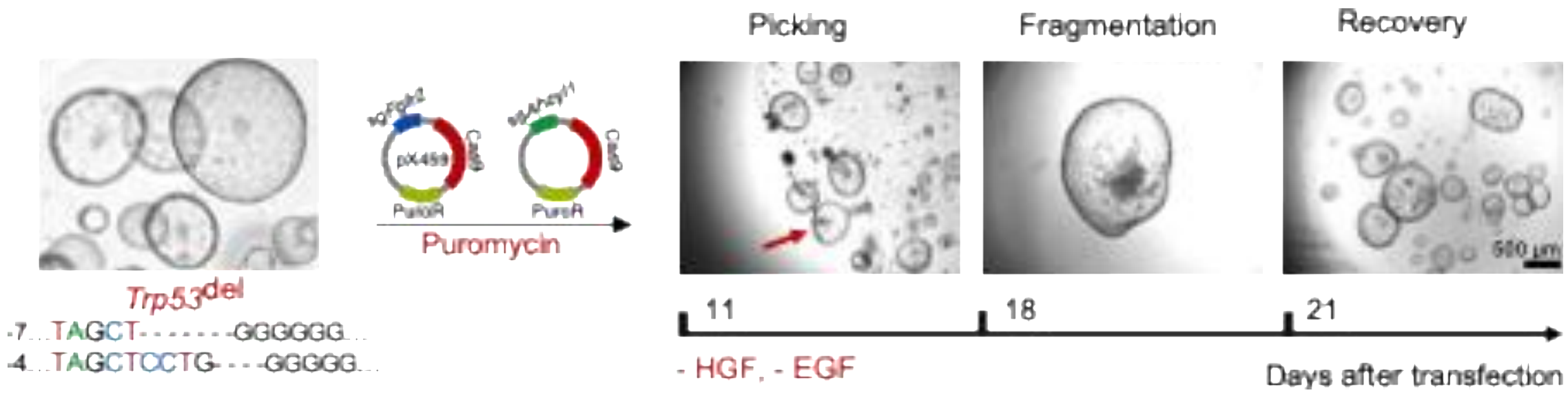

Fig. S1. Work flow of generating an *Fgfr2* fusion containing murine cholangiocyte organoid line.

Trp53 deleted cholangiocyte organoid line was transfected with 2 plasmids containing each an sgRNA targeting the respective intronic regions of *Fgfr2* and *Ahcyl1*. After transfection, functional selection with growth factor withdrawal allowed picking of single organoid clones which were then expanded and analysed for presence of *Fgfr2::Ahcyl1* fusion.

Fig. S2

A. AHCYL1

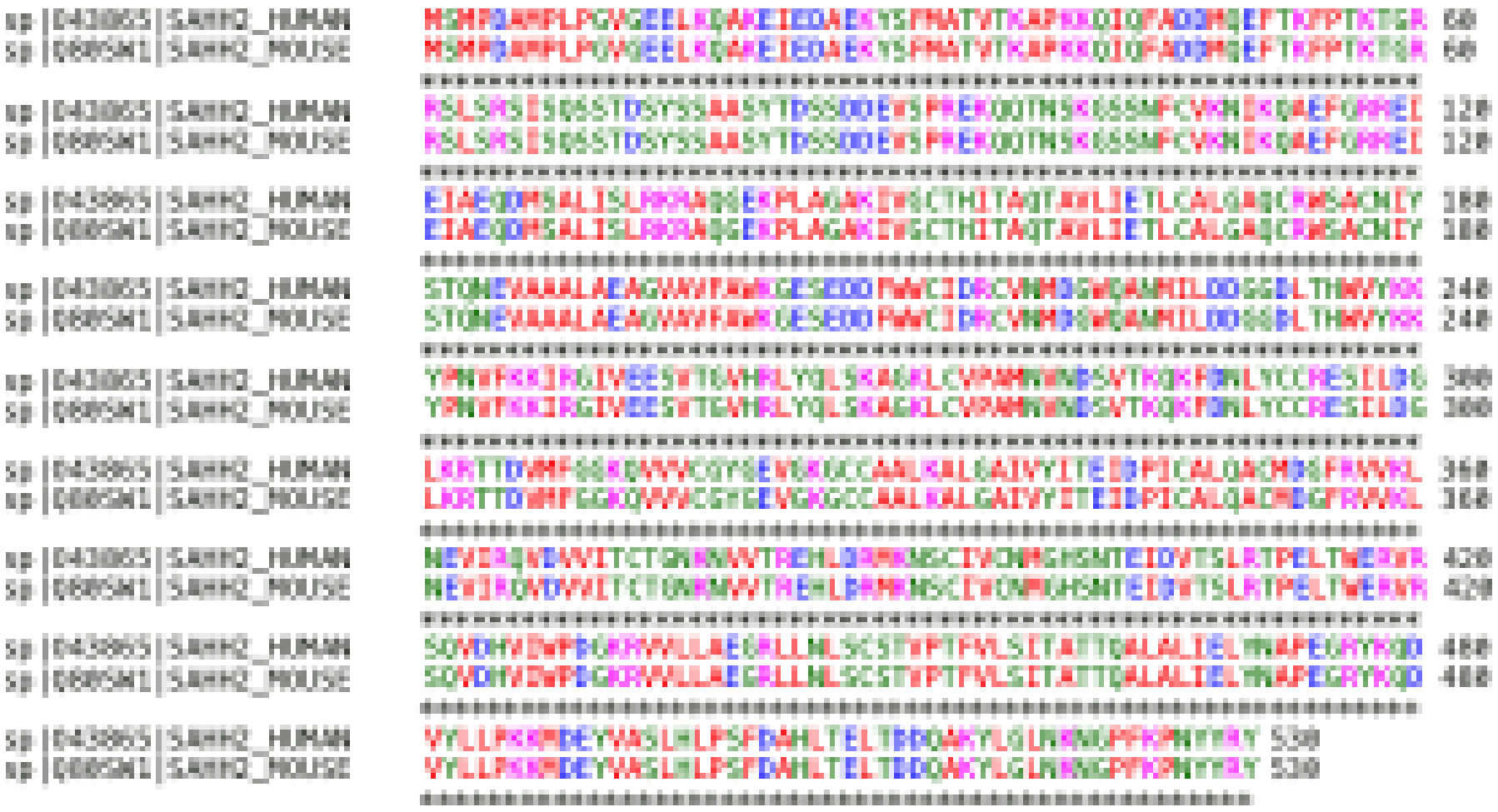

B. BICC1

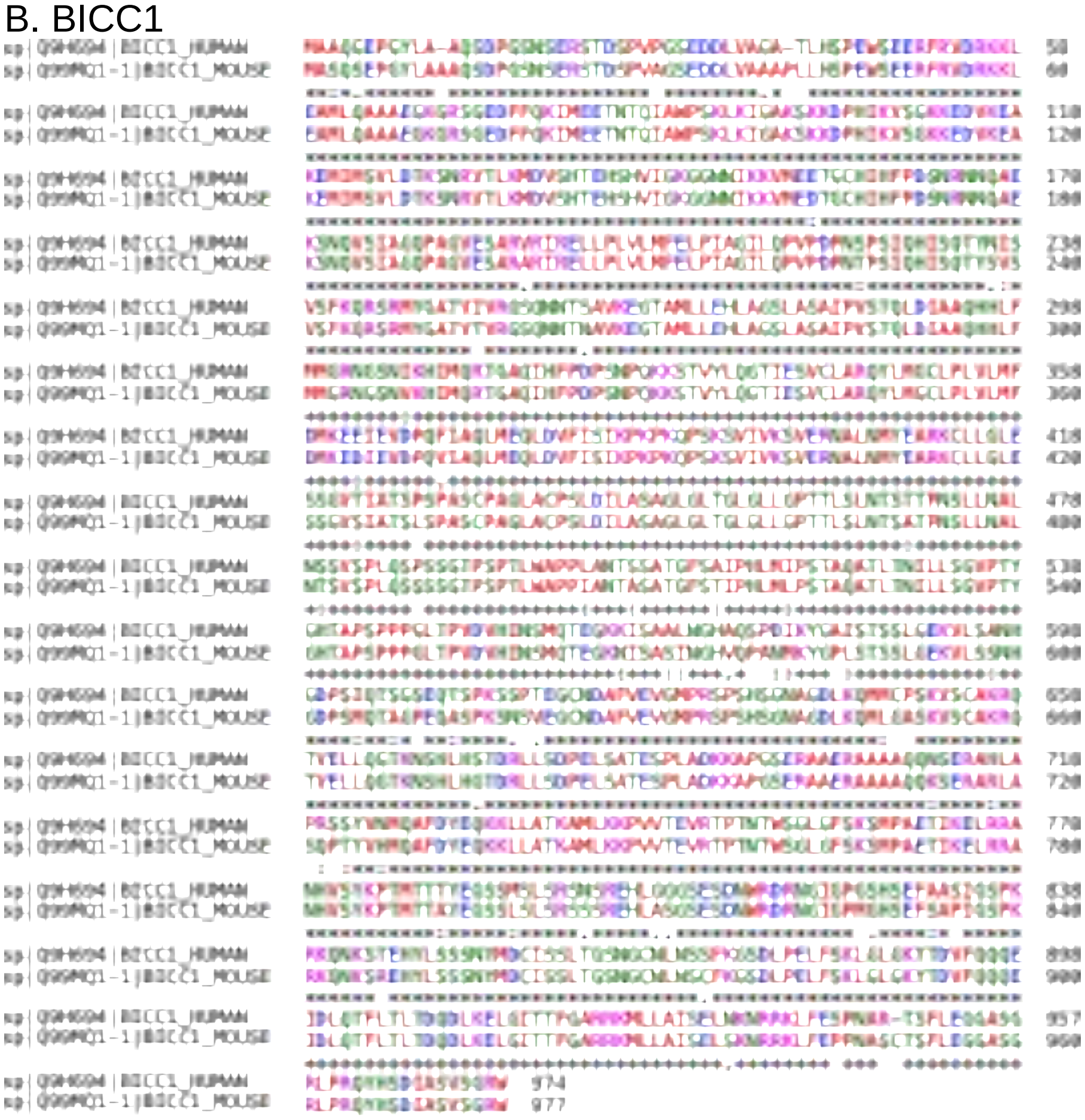

C. TACC3

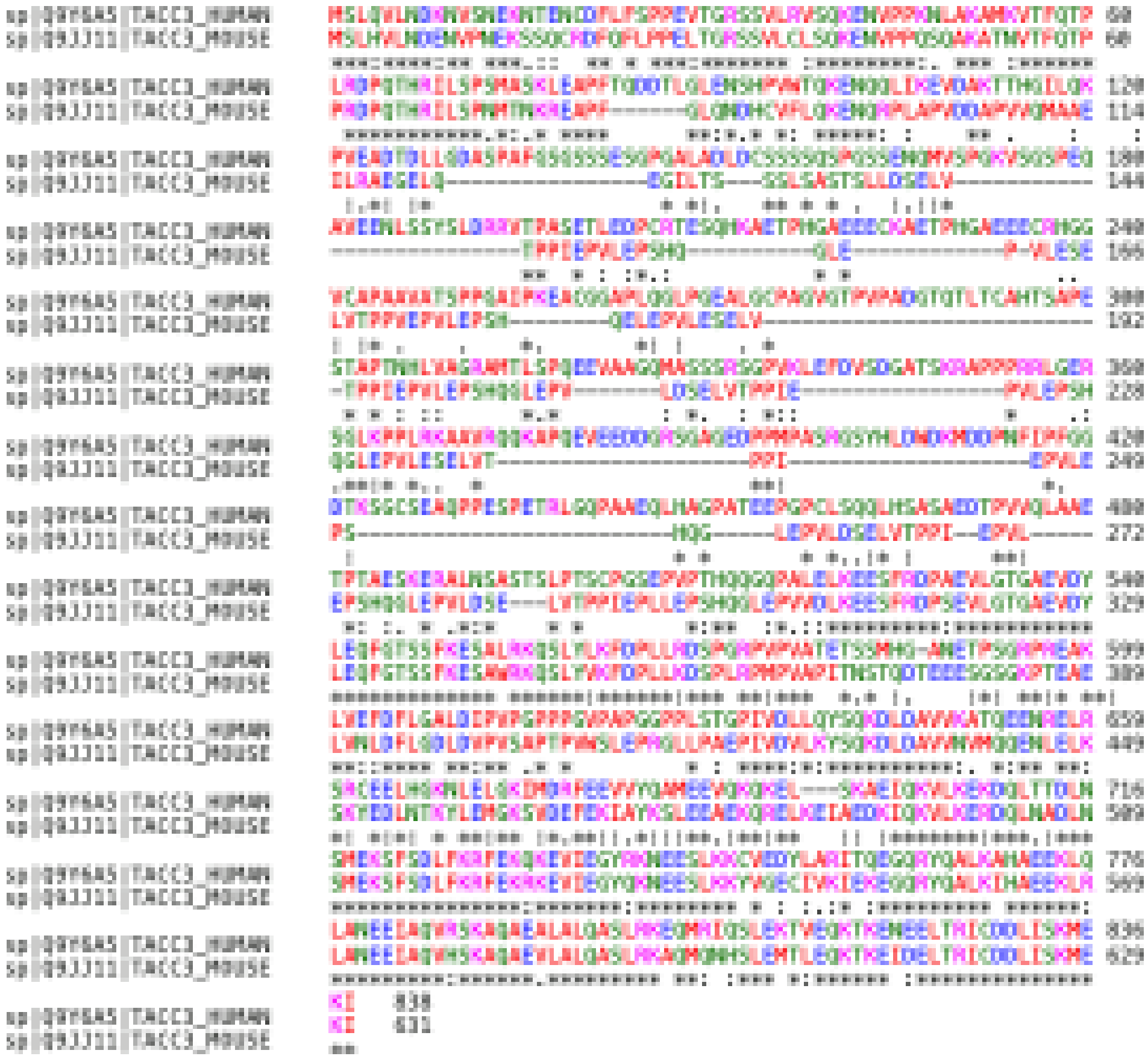

Fig. S2. Protein sequence alignments of the selected three FGFR2 fusion partners.

Amino acid sequences of the respective human and murine proteins **(A)** AHCYL1, **(B)** BICC1, and **(C)** TACC3 were extracted from the Uniprot data base[22] and aligned with Clustal Omega[23] .

Fig. S3

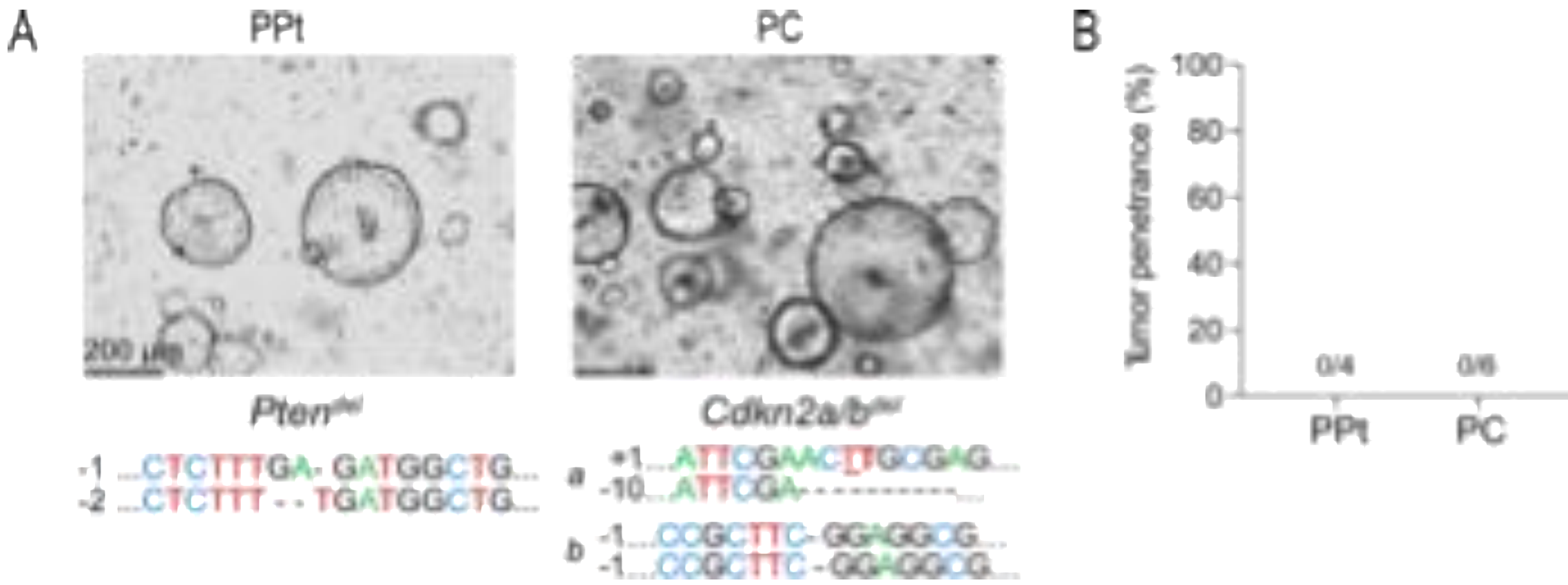

Fig. S3. Generation of PPt and PC control cholangiocyte organoid lines.
(A) Microscopic bright field images of cholangiocyte organoid lines harboring *Trp53* and *Pten* gene deletion (PPt) or *Trp53* and *Cdkn2a* and *Cdkn2b* gene deletion (PC) and respective genetic alterations specified below. Scale bar indicates 200 µm. **(B)** Bar plot indicating lack of intrahepatic tumor development upon implantation with the respective number of mice tested.

Fig. S4

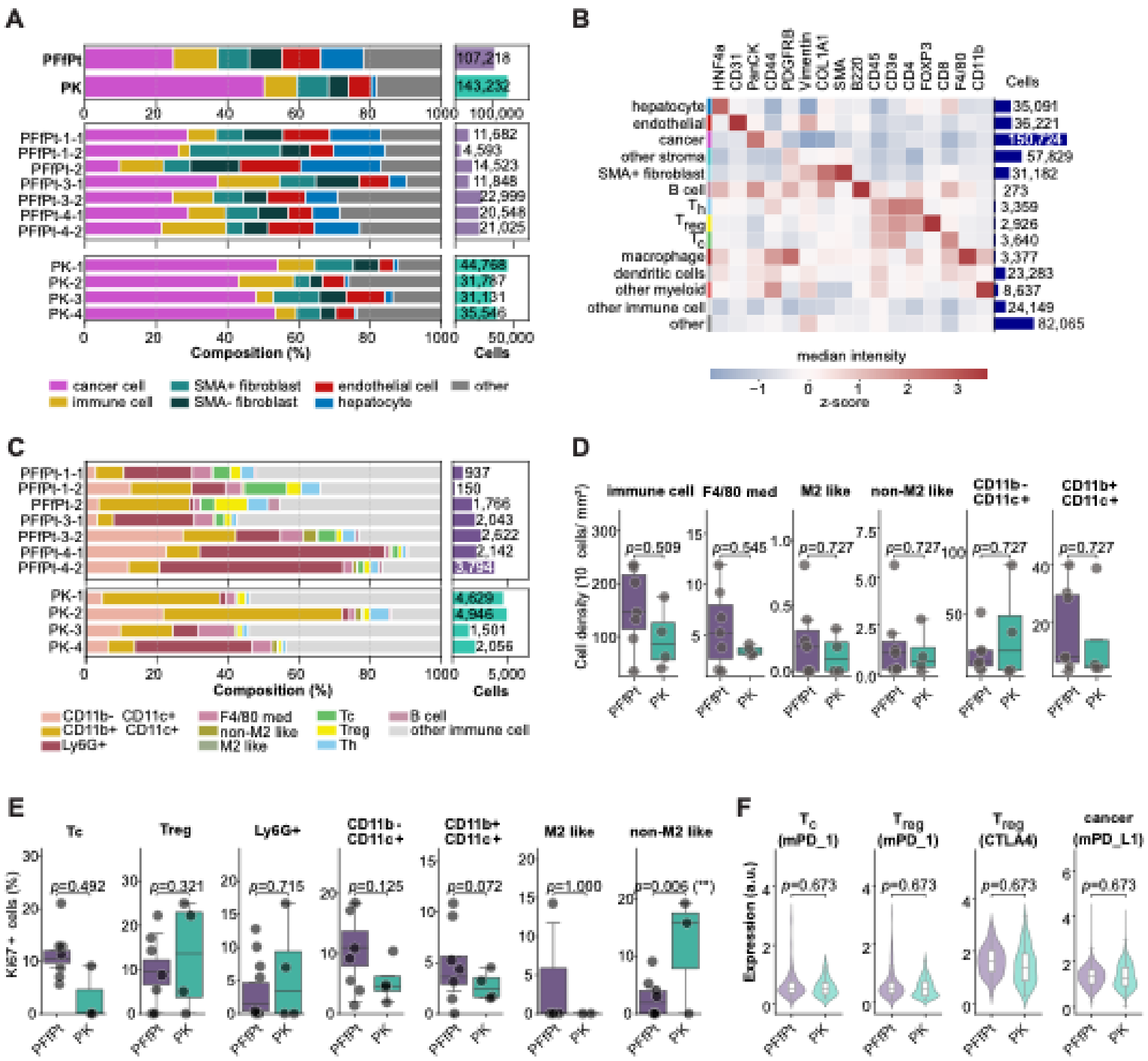

Fig. S4. In-depth analysis of TME cell populations via spatial proteomics. **(A)** Stacked bar plots showing the broad cell lineage composition across samples. Total cell count per imaged tumor is indicated to the right. **(B)** Heatmap displaying the median marker intensity of lineage markers for each specified cell type. Total number of annotated cell type is indicated to the right. **(C)** Stacked bar plots showing the immune cell type composition across samples. Total immune cell count per imaged tumor is indicated to the right. Box plots depicting cell densities of indicated cell populations **(D)** and proportion of Ki67^{+} cells per indicated cell type **(E)**. Box indicates interquartile range (IQR) and whiskers 1.5xIQR with bold line median; each data point indicates a tumor; exact p-values are indicated in the figure (binomial generalized linear mixed-effects model on 4 animal replicates each with a significance threshold of p <0.05). **(F)** Violin plots with integrated box plots depicting immune checkpoint expression intensities for the indicated cell types. No statistically significant differences between PFfPt and PK iCCA was observed.

Fig. S5

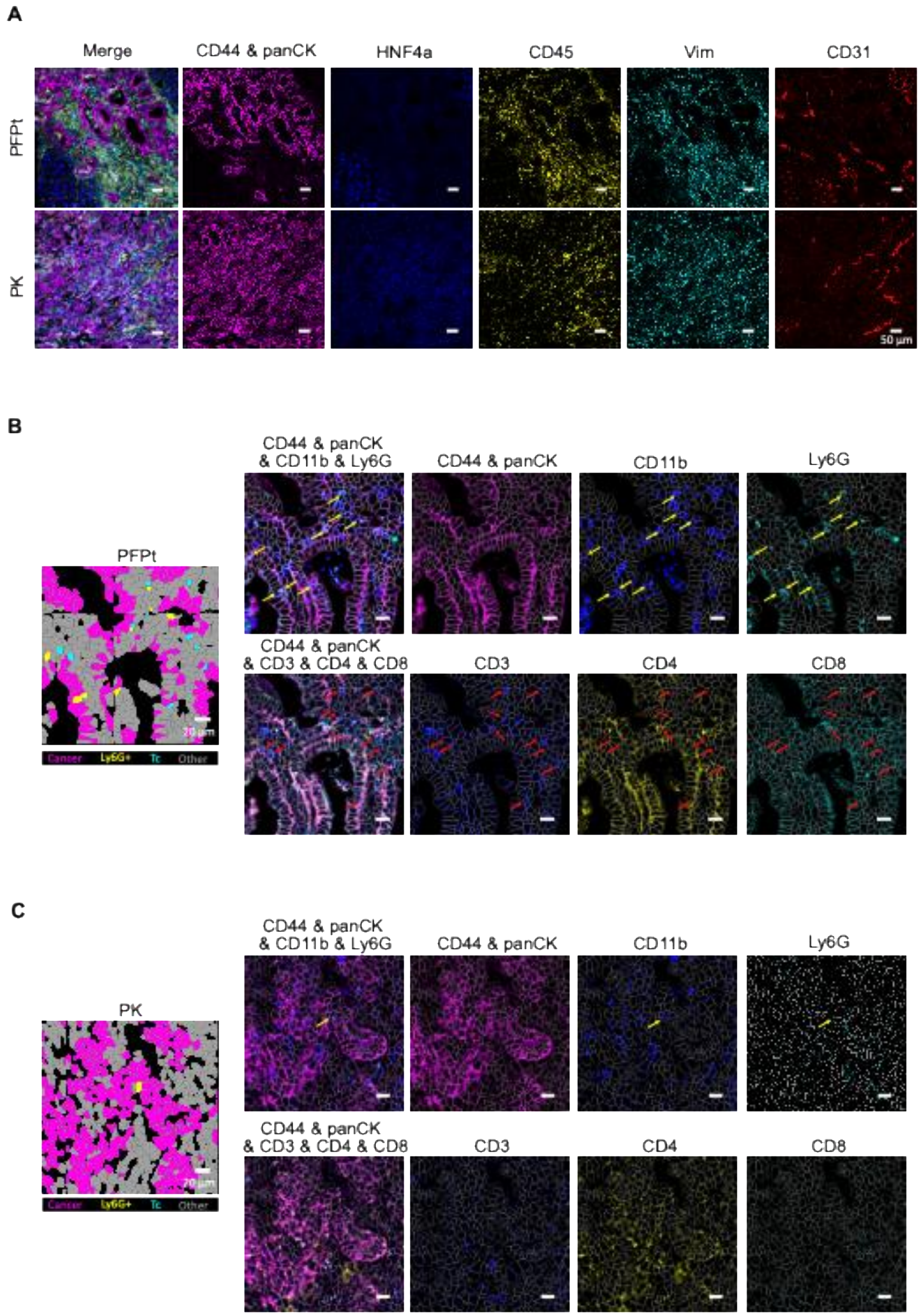
A
Merge
CD44 & panCK
HNF4a
CD45
Vim
CD31
PFPt
PK
50 µm
B
PFPt
20 µm
Cancer
Ly6G+
Tc
Other
CD44 & panCK & CD11b & Ly6G
CD44 & panCK
CD11b
Ly6G
CD44 & panCK & CD3 & CD4 & CD8
CD3
CD4
CD8
C
PK
20 µm
Cancer
Ly6G+
Tc
Other
CD44 & panCK & CD11b & Ly6G
CD44 & panCK
CD11b
Ly6G
CD44 & panCK & CD3 & CD4 & CD8
CD3
CD4
CD8

Fig. S5. Single-channel MIBI images to illustrate gating strategies.

(A) Single-channel MIBI images with indicated antibodies with the respective merged picture in **Figure 4C**. (**B-C**) Single-channel MIBI images with indicated antibodies to illustrate the gating strategy for the final cell annotation image to the left (**Figure 4H**). Red and yellow arrows highlight the cells in each channel considered positive in the annotation.

All scale bars indicate 20 µm.

Fig. S6

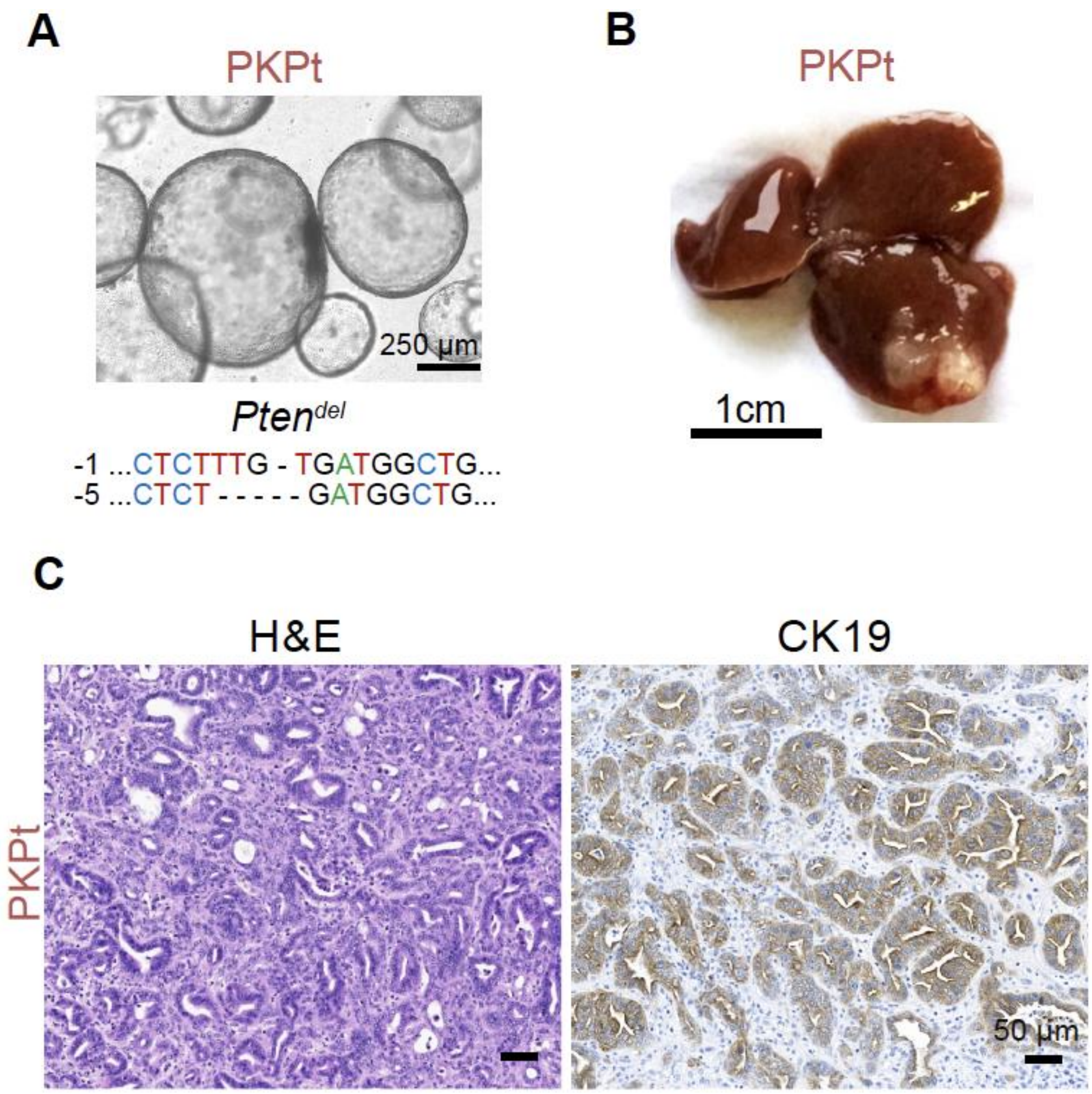

Fig. S6. Generation of PK line with *Pten* deletion.

(A) Microscopic bright-field image of PKPt CCA-organoid line and respective genetic alterations specified below. **(B)** Representative macroscopic image of PKPt iCCA. **(C)** Representative histologic pictures of PKPt iCCA for H&E and CK19 immunostain. Scale bars indicate 50 µm.

Fig. S7

```
sp|P21802|FGFR2_HUMAN      LTLGKPLGEGCFGQVVMAEAVGIDKDKPKEAVTVAVKMLKDDATEKDLSDLVSEMEMMKM 540
sp|P21803|FGFR2_MOUSE      LTLGKPLGEGCFGQVVMAEAVGIDKDKPKEAVTVAVKMLKDDATEKDLSDLVSEMEMMKM 540
                           ************************************************************

sp|P21802|FGFR2_HUMAN      IGKHKNIINLLGACTQDGPLYVIVEYASKGNLREYLRARRPPGMEYSYDINRVPEEQMTF 600
sp|P21803|FGFR2_MOUSE      IGKHKNIINLLGACTQDGPLYVIVEYASKGNLREYLRARRPPGMEYSYDINRVPEEQMTF 600
                           ************************************************************

sp|P21802|FGFR2_HUMAN      KDLVSCTYQLARGMEYLASQKCIHRDLAARNVLVTENNVMKIADFGLARDINNIDYYKKT 660
sp|P21803|FGFR2_MOUSE      KDLVSCTYQLARGMEYLASQKCIHRDLAARNVLVTENNVMKIADFGLARDINNIDYYKKT 660
                           ************************************************************

sp|P21802|FGFR2_HUMAN      TNGRLPVKWMAPEALFDRVYTHQSDVWSFGVLMWEIFTLGGSPYPGIPVEELFKLLKEGH 720
sp|P21803|FGFR2_MOUSE      TNGRLPVKWMAPEALFDRVYTHQSDVWSFGVLMWEIFTLGGSPYPGIPVEELFKLLKEGH 720
                           ************************************************************

sp|P21802|FGFR2_HUMAN      RMDKPANCTNELYMMMRDCWHAVPSQRPTFKQLVEDLDRILTLTTNEEYL    770
sp|P21803|FGFR2_MOUSE      RMDKPTNCTNELYMMMRDCWHAVPSQRPTFKQLVEDLDRILTLTTNEEYL    770
                           *****:********************************************
```

Fig. S7. Protein sequence alignments of the kinase domain of FGFR2.
Amino acid sequences of the respective human and murine kinase domain of FGFR2, which is > 99% conserved. Sequences were extracted from the Uniprot data base[22] and aligned with Clustal Omega[23].

Fig. S8

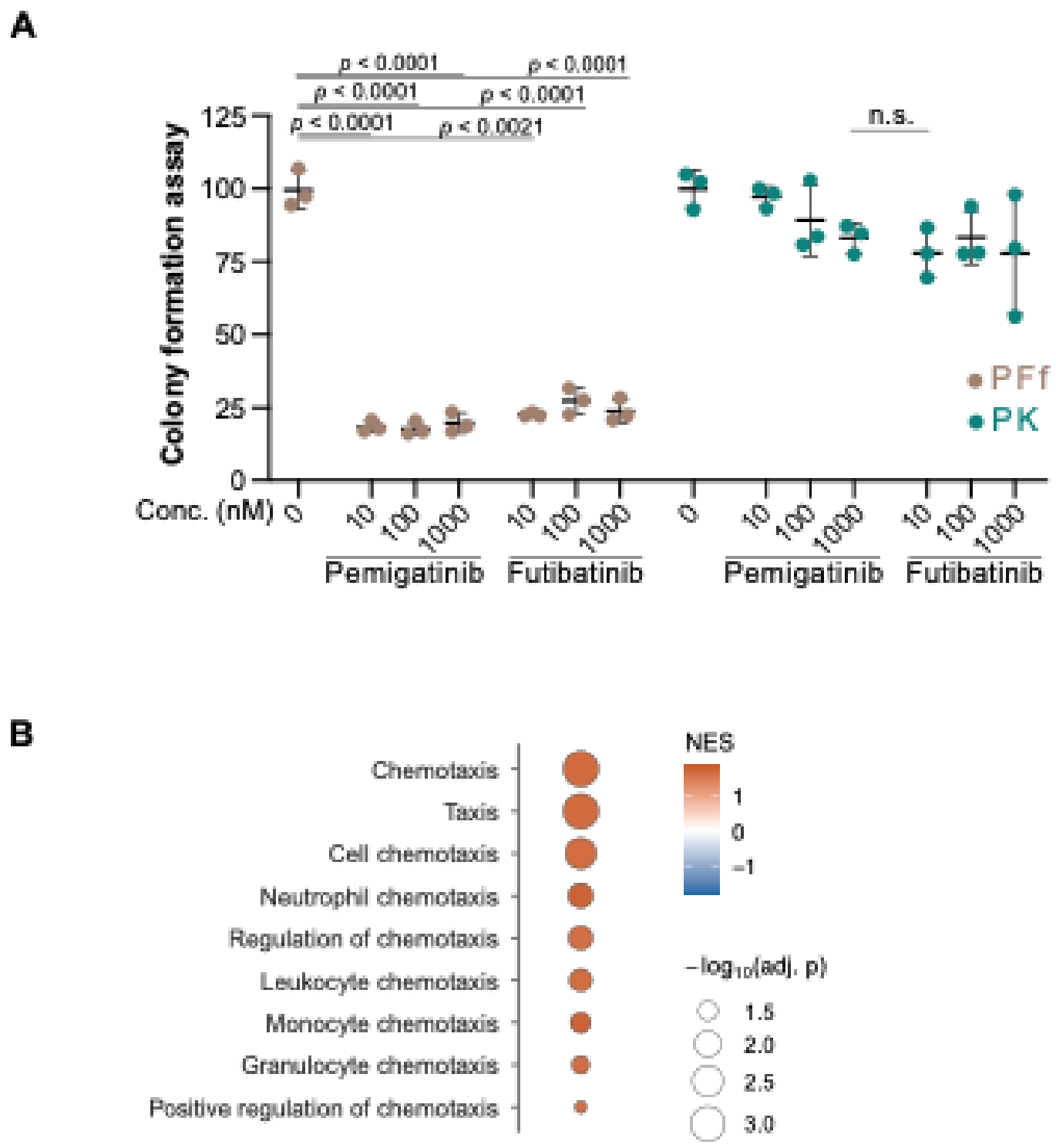

Fig. S8. FGFR inhibitor treatment *in vitro*.

(**A**) Scatter plot depicting results of colony formation of PFf and PK organoids (illustrated in **Figure 6**) treated with the indicated concentrations of pemigatinib and futibatinib normalized to DMSO control. N=3 per condition with line indicating mean±SD; exact p-values shown in figure with p≥0.05 considered not significant (One-way-ANOVA with Welch's correction). (**B**) GSEA of GO BP terms selected by semantic similarity to the chemotaxis term highlights enriched neutrophil and granulocyte migration signatures in PFf organoids.

Table S1. Lists of Oligonucleotides

sgRNAs

Targeted gene	sgRNA sequence (5´… 3´)	Source/design tool
Ahcyl1	TATTATAGGGTCTAAACAGT	Benchling [Biology Software] 2022
Bap1	GCTCTTCGATCCATTTGAAC	Artegiani et al. *Cell Stem Cell*, 2019; DOI: 10.1016/j.stem.2019.04.017
Bicc1	TGTTGCATACTCGTGATCCC	Benchling [Biology Software] 2022
Cdkn2a	ACGCCCTGAACCGCTTCGGG	Synthego CRISPR design tool
Cdkn2b	ACGCCCTGAACCGCTTCGGG	Synthego CRISPR design tool
Fgfr2	AAGTACTTACTAACTGACCC	Benchling [Biology Software] 2022
Pbrm1	GAAACCACTTCATAATAGTC	Synthego CRISPR design tool
Pten	GCTAACGATCTCTTTGATGA	Weber et al., *PNAS*, 2015 DOI: 10.1073/pnas.1512392112
Tacc3	ACAGGCACCAGTACCGACCT	Benchling [Biology Software] 2022
Trp53	GTGTAATAGCTCCTGCATGG	Platt et al., *Cell*, 2014 DOI: 10.1016/j.cell.2014.09.014

Primers for genomic PCR

Gene	Gene-ID	Primer Sequence (5´… 3´)
Ahcyl1	ENSMUSG00000027893	F: TGCAGTGTCACCCCAGCAGAGA<br>R: ACTTAGGCTAGGGGCTGGTGAGA
Bap1	ENSMUSG00000021901	F: CTGGTTTTGCAGCCGGAACCCT<br>R: AGCATCCTGGCCCGAAACAAGC
Bicc1	ENSMUSG00000014329	F: TGGCTATGTCGCAATTCTGGGTGT<br>R: TGGGTTCTGGGGCTTCGAACTCA
Cdkn2a	ENSMUSG00000044303	F: CTCCCGGCGATGTTCTACAGGA<br>R: AAAACTTTCCCCTGCGAAGGCC
Cdkn2b	ENSMUSG00000073802	F: ACGCTCACCGAAGCTACTGGGT<br>R: TTCTGTTGGGAGGGCTGTGCCT
Fgfr2	ENSMUSG00000030849	F: GTCTGCGCTTTTTGCCCCCTCT<br>R: CTGGCTGCTACCAAGGTCCCTT
Fgfr2-Ahcyl1 Fusion	-	F: GTCTGCGCTTTTTGCCCCCTCT<br>R: ACTTAGGCTAGGGGCTGGTGAGA
Fgfr2-Bicc1 Fusion	-	F: GTCTGCGCTTTTTGCCCCCTCT<br>R: AAGCTATGCTTGGGTCCGCAGC
Fgfr2-Tacc3 Fusion	-	F: GTCTGCGCTTTTTGCCCCCTCT<br>R: AGCTCCAAGTTCTCCTGCTGCA
Pbrm1	ENSMUSG00000042323	F: CACCAGCCCTGCCTTGCTCATT<br>R: CCACTGCCTGGCCCAAGAACTG
Pten	ENSMUSG00000013663	F: TCTTCTCCCCATTCCGCTGCCT<br>R: AAGGCTGGATTGACGAGCTCGC

Tacc3	ENSMUST00000079534	F: AGCCGACAGAAGCTGAGCTTGTCA<br>R: GAACAGCTGGTGGCCAAAACAGAT
Trp53	ENSMUSG00000059552	F: ATAGAGACGCTGAGTCCGGTTC<br>R: CCTAAGCCCAAGAGGAAACAGA

Primers for quantitative RT-PCR

Transcript	Transcript-ID	Primer Sequence (5´… 3´)
Ahcyl1	ENSMUST00000029490	F: CCAAGGAGATCGAGGACGCCGA<br>R: ACGAGACAAAGATCTCCGGCCA
Fgfr2	ENSMUST00000122054	F: GGCATGCTGTACCCTCACAG<br>R: GTGAGATCCAAGTATTCCTCATTGG
Fgfr2-Ahcyl1 Fusion	-	F: GACAAGCCCACCAACTGCACCA<br>R: ACGAGACAAAGATCTCCGGCCA
Gapdh	ENSMUST00000117757	F: CATCACTGCCACCCAGAAGACTG<br>R: ATGCCAGTGAGCTTCCCGTTCAG
Cxcl9	*ENSMUST00000113093.5*	*F: CCTAGTGATAAGGAATGCACGATG*<br>*R: CTAGGCAGGTTTGATCTCCGTTC*
Cxcl10	*ENSMUSG00000034855*	*F: ATCATCCCTGCGAGCCTATCCT*<br>*R: GACCTTTTTTGGCTAAACGCTTTC*
Cxcl1	*ENSMUSG00000029380*	*F: TCCAGAGCTTGAAGGTGTTGCC*<br>*R:AACCAAGGGAGCTTCAGGGTCA*
Cxcl2	*ENSMUST00000200681.3*	*F: CATCCAGAGCTTGAGTGTGACG*<br>*R: GGCTTCAGGGTCAAGGCAAACT*
Tnf	*ENSMUSG00000024401*	*F: GGTGCCTATGTCTCAGCCTCTT*<br>*R: GCCATAGAACTGATGAGAGGGAG*
Etv4	*ENSMUSG00000017724*	*F: CACAGACTTCGCCTACGACTCA*<br>*R: GCAGACATCATCTGGGAATGGTC*
Dusp6	*ENSMUSG00000019960*	*F: CTCGGATCACTGGAGCCAAAAC*<br>*R: TCTGCATGAGGTACGCCACTGT*
Rps14	*ENSMUSG00000024608*	*F: GACCAAGACCCCTGGACCT*<br>*R: CCCCTTTTCTTCGAGTGCTA*
Prf1	*ENSMUST00000035419.7*	*F: ACACAGTAGAGTGTCGCATGTAC*<br>*R: GTGGAGCTGTTAAAGTTGCGGG*

Table S2. List of antibodies

Antibodies	Source	Catalog number	RRID	Application
Anti-ACHYL1 Antibody	Proteintech	10658-3-AP	AB_2226013	1:1000,WB
Anti-mouse IgG, HRP-linked Antibody	Cell Signaling Technology	7076P2	AB_330924	1:3000, WB
Anti-rabbit IgG, HRP-linked Antibody	Cell Signaling Technology	7074P2	AB_2099233	1:3000, WB
CD8a Monoclonal Antibody (4SM15)	Thermo Fisher Scientific	14-0808-82	AB_2572861	1:100, IHC
CK19	Developmental Studies Hybridoma Bank	TROMA-III	AB_2133570	1:10, IHC
FGF receptor 2 (clone D4L2V)	Cell Signaling Technology	23328S	AB_2798862	1:500, WB
FOXP3 Monoclonal Antibody (FJK-16s)	Thermo Fisher Scientific	14-5773-82	AB_467576	1:100, IHC
Ly-6G/Ly-6C Monoclonal Antibody (RB6-8C5),	Thermo Fisher	14-5931-85	AB_467731	1:100, IHC
Rabbit Anti-Rat IgG Antibody (H+L), Biotinylated	Vector Laboratories	BA-4000	AB_2336206	1:1000, IHC
Vinculin Antibody (7F9) HRP	Santa Cruz Biotechnology	sc-73614 HRP	AB_2941767	1:1000, WB
β-Actin Antibody (C4) HRP	Santa Cruz Biotechnology	sc-47778 HRP	AB_2714189	1:5000, WB
Gmzb anti mouse monoclonal E5V2L	Cell signaling	44153	AB_2857976	1:200 IF
PD1 anti mouse Polyclonal	R&D	AF1021	AB_354541	1:100 IF
Anti mouse CD8a monoclonal D4W2Z	Cell signaling	98941S	AB_2756376	1:400 IF
Rabbit anti goat	Dako	P04490	-	1:300, IF
