# Supplementary material for: FGFR2 fusion-driven cholangiocarcinoma is characterized by a distinct neutrophil-enriched tumor microenvironment in a syngeneic murine model
Source: JHEP Rep. 2026 Jul 21;8(10):101964. doi: 10.1016/j.jhepr.2026.101964 (PMC13598188; doi:10.1016/j.jhepr.2026.101964)
Supplement: Supplementary file 4 [file mmc2.docx]

**JHEP Reports**

**CTAT methods**

Tables for a “Complete, Transparent, Accurate and Timely account” (CTAT) are now mandatory for all revised submissions. The aim is to enhance the reproducibility of methods.

- Only include the parts relevant to your study
- Refer to the CTAT in the main text as ‘Supplementary CTAT Table’
- Do not add subheadings
- Add as many rows as needed to include all information
- Only include one item per row

**If the CTAT form is not relevant to your study, please outline the reasons why:**

1.1 **Antibodies**

**WesternBlot**

| **Name** | **Citation (RRID)** | **Supplier** | **Cat no.** | **Clone no.** | **Dilution** |
| --- | --- | --- | --- | --- | --- |
| ACHYL1 | AB_2226013 | Proteintech | 10658-3-AP | polyclonal | 1:1000 |
| Anti-mouse IgG, HRP-linked Antibody | AB_330924 | Cell Signaling Technology | 7076P2 | polyclonal | 1:3000 |
| Anti-rabbit IgG, HRP-linked Antibody | AB_2099233 | Cell Signaling Technology | 7074P2 | polyclonal | 1:3000 |
| FGFR2 | AB_2798862 | Cell Signaling Technology | 23328S | D4L2V | 1:500 |
| PTEN | AB_390810 | Cell Signaling Technology | 9559 | 138G6 | 1:1000 |
| Vinculin | AB_2941767 | Santa Cruz Biotechnology | sc-73614 HRP | 7F9 | 1:1000 |
| β-Actin | AB_2714189 | Santa Cruz Biotechnology | sc-47778 HRP | C4 | 1:5000 |

**IF/IHC**

| **Name** | **Citation (RRID)** | **Supplier** | **Cat no.** | **Clone no.** | **Application** |
| --- | --- | --- | --- | --- | --- |
| Anti mouse CD8a | AB_2756376 | Cell Signaling Technology | 98941S | **D4W2Z** | 1:400 IF |
| CD11b anti human | AB_2753196 | Abcam | ab133357 | **EPR1344** | 1:4000, IF |
| CD15 anti human | AB_314194 | BioLegend | 301902 | **HI98** | 1:500, IF |
| CD8a | AB_2572861 | Thermo Fisher Scientific | 14-0808-82 | **4SM15** | 1:100, IHC |
| CK19 | AB_2133570 | Developmental Studies Hybridoma Bank | TROMA-III | TROMA-III | 1:10, IHC/IF |
| Donkey anti-Mouse IgG | AB_141607 | nvitrogen | A-21202 | **Polyclonal** | 1:1000, IF |
| Donkey anti-Rabbit IgG | AB_141637 | Invitrogen | A-21207 | **Polyclonal** | 1:1000, IF |
| FOXP3 | AB_467576 | Thermo Fisher Scientific | 14-5773-82 | **FJK-16s** | 1:100, IHC |
| ‍Gmzb anti mouse | AB_2857976 | Cell Signaling Technology | 44153 | **E5V2L** | 1:200 IF |
| Ki67 | AB_10979488 | Invitrogen | MA5-14520 | **SolA15** | 1:100, IF |
| Ly-6G/Ly-6C | AB_467731 | Thermo Fisher Scientific | 14-5931-85 | **RB6-8C5** | 1:100, IHC |
| Ly-6G/Ly-6C | AB_467731 | Thermo Fisher Scientific | 14-5931-85 | **RB6-8C5** | 1:100, IHC |
| ‍PD1 anti mouse | AB_354541 | R and D Systems | AF1021 | **Polyclonal** | 1:100 IF |
| Rabbit anti goat | AB_2617143 | Dako | P04490 | **Polyclonal** | 1:300, IF |
| Rabbit Anti-Rat IgG | AB_2336206 | Vector Laboratories | BA-4000 | **Polyclonal** | 1:1000, IHC |
| Rabbit Anti-Rat IgG | AB_2336206 | Vector Laboratories | BA-4000 | **Polyclonal** | 1:1000, IHC |

**MIBI Antibodies**

| **Name** | **Citation (RRID)** | **Supplier** | **Cat no.** | **Clone no.** | **Conc. (μg/mL)** |
| --- | --- | --- | --- | --- | --- |
| ATP5A | AB_301447 | Abcam | ab14748 | 15H4C4 | 1 |
| B220 | AB_493535 | BioLegend | 103249 | RA3-6B2 | 1 |
| cCaps3 | AB_2341188 | Cell Signaling Technology | 74860 | D3E9 | 1 |
| CD11b | AB_2753196 | Abcam | ab133357 | EPR1344 | 1 |
| CD11c | AB_2800005 | Cell Signaling Technology | 97585 | D1V9Y | 1 |
| CD163 | AB_1603775 | Abcam | ab213612 | EPR19518 | 3 |
| CD31 | AB_2161028 | R&D | AF3628 | poly | 1,5 |
| CD3e | - | Ionpath | 715904-20UG | EPR22667-12 | 1 |
| CD4 | AB_2573006 | eBioscience | 14-9766-82 | 4SM95 | 1 |
| CD44 | AB_312963 | BioLegend | 103002 | IM7 | 1 |
| CD45 | - | Ionpath | 717503-20UG | D3F8Q | 0,5 |
| CD8 | AB_2572861 | eBioscience | 14-0808-82 | 4SM15 | 3 |
| COL1A1 | AB_2799776 | Cell Signaling Technology | 72026 | E8F4L | 5 |
| CTLA4 | - | MSVA | 3451-152R-01 | MSVA-152R | 2 |
| F4/80 | AB_2799771 | Cell Signaling Technology | 70076 | D2S9R | 0,5 |
| FoxP3 | AB_467576 | Invitrogen | 14-5773-82 | FJK-16S | 2 |
| G6PD | AB_2893162 | abcam | ab231828 | EPR20668 | 0,5 |
| GLUT1 | AB_10903230 | Abcam | ab252403 | EPR3915 | 0,5 |
| HH3 | AB_331535 | Cell Signaling Technology | 60932 | D1H2 | 1 |
| HNF4a | AB_777975 | Abcam | ab41898 | K9218 | 1 |
| LDH | AB_880283 | Abcam | ab219591 | EP1566Y | 1,5 |
| Ly6G | AB_1186101 | BioLegend | 127602 | 1A8 | 1 |
| mKi67 | AB_2564285 | BioLegend | 652402 | 16A4 | 1 |
| mouse IgG (for PD1) | AB_2739144 | Abcam | ab102445 | polyclonal | 1 |
| mPD-L1 | AB_2799686 | Cell Signaling Technology | 64988 | D5V3B | N/A (1:50) |
| Na^+^/K^+^ ATPase | AB_1310692 | Abcam | ab167390 | EP1845Y | 1 |
| panCK | AB_1603775 | Abcam | ab80826 | AE1/AE3 | 2 |
| PD1/CD279 | AB_2881611 | Proteintech | 66220-1-Ig | 4H4D1 | 0,375 |
| PDGFRB | AB_2162497 | Cell Signaling Technology | 45176 | 28E1 | 1 |
| Rabbit IgG (for PD-L1) | AB_955447 | Abcam | ab7085 | poly | 1 |
| Rat IgG (for CD8a) | AB_10674954 | Abcam | ab102248 | poly | 1 |
| SMA | AB_2734735 | Cell Signaling Technology | 50513 | D4K9N | 0,25 |
| Vimentin | AB_10695459 | Cell Signaling Technology | 46173 | D21H3 | 0,5 |

1.2 **Cell lines**

| **Name** | **Citation** | **Supplier** | **Cat no.** | **Passage no.** | **Authentication test method** |
| --- | --- | --- | --- | --- | --- |
| Primary cholangiocyte organoids’’ from C57Bl/6J mice | N/A | Primary cells established in the lab | N/A | N/A | N/A |

1.3 **Organisms**

| **Name** | **Citation** | **Supplier** | **Strain** | **Sex** | **Age** | **Overall n number** |
| --- | --- | --- | --- | --- | --- | --- |
| C57BL/6J mice | RRID:IMSR_JAX:000664 | Janvier Labs | C57BL/6J | M/F | ≥8 weeks | 76 |
| NOD.Cg-*Prkdc^scid^ Il2rg^tm1Wjl^* mice | RRID:IMSR_JAX:005557 | DKFZ | NSG | M | ≥8 weeks | 5 |

1.4 **Sequence based reagents**

### **sgRNAs**

| **Targeted gene** | **Single Guide RNA sequence**  **(5´… 3´)** | **Source/design tool** |
| --- | --- | --- |
| *Ahcyl1* | TATTATAGGGTCTAAACAGT | Benchling [Biology Software] 2022 |
| *Bap1* | GCTCTTCGATCCATTTGAAC | Artegiani et al. *Cell Stem Cell*, 2019; DOI: 10.1016/j.stem.2019.04.017 |
| *Bicc1* | TGTTGCATACTCGTGATCCC | Benchling [Biology Software] 2022 |
| *Cdkn2a* | ACGCCCTGAACCGCTTCGGG | Synthego CRISPR design tool |
| *Cdkn2b* | ACGCCCTGAACCGCTTCGGG | Synthego CRISPR design tool |
| *Fgfr2* | AAGTACTTACTAACTGACCC | Benchling [Biology Software] 2022 |
| *‍Kras* | GCAGCGTTACCTCTATCGTA | Platt et al., *Cell*, 2014 DOI: 10.1016/j.cell.2014.09.014 |
| *Pbrm1* | GAAACCACTTCATAATAGTC | Synthego CRISPR design tool |
| *Pten* | GCTAACGATCTCTTTGATGA | Weber et al., *PNAS*, 2015DOI: 10.1073/pnas.1512392112 |
| *Tacc3* | ACAGGCACCAGTACCGACCT | Benchling [Biology Software] 2022 |
| *Trp53* | GTGTAATAGCTCCTGCATGG | Platt et al., *Cell*, 2014 DOI: 10.1016/j.cell.2014.09.014 |

### **Primers for genomic PCR**

| Gene | Gene-ID | Primer Sequence  (5´… 3´) |
| --- | --- | --- |
| *Ahcyl1* | ENSMUSG00000027893 | F: TGCAGTGTCACCCCAGCAGAGA  R: ACTTAGGCTAGGGGCTGGTGAGA |
| *Bap1* | ENSMUSG00000021901 | F: CTGGTTTTGCAGCCGGAACCCT  R: AGCATCCTGGCCCGAAACAAGC |
| *Bicc1* | ENSMUSG00000014329 | F: TGGCTATGTCGCAATTCTGGGTGT  R: TGGGTTCTGGGGCTTCGAACTCA |
| *Cdkn2a* | ENSMUSG00000044303 | F: CTCCCGGCGATGTTCTACAGGA  R: AAAACTTTCCCCTGCGAAGGCC |
| *Cdkn2b* | ENSMUSG00000073802 | F: ACGCTCACCGAAGCTACTGGGT  R: TTCTGTTGGGAGGGCTGTGCCT |
| *Fgfr2* | ENSMUSG00000030849 | F: GTCTGCGCTTTTTGCCCCCTCT  R: CTGGCTGCTACCAAGGTCCCTT |
| *Fgfr2-Ahcyl1 Fusion* | - | F: GTCTGCGCTTTTTGCCCCCTCT  R: ACTTAGGCTAGGGGCTGGTGAGA |
| *Fgfr2-Bicc1 Fusion* | - | F: GTCTGCGCTTTTTGCCCCCTCT  R: AAGCTATGCTTGGGTCCGCAGC |
| *Fgfr2-Tacc3 Fusion* | - | F: GTCTGCGCTTTTTGCCCCCTCT  R: AGCTCCAAGTTCTCCTGCTGCA |
| *‍Kras* | ENSMUSG00000030265 | F: TTTGTTGCCCTTTATTGCAGAA  R: ATGCAAATACAAAGCACGGATG |
| *Pbrm1* | ENSMUSG00000042323 | F: CACCAGCCCTGCCTTGCTCATT  R: CCACTGCCTGGCCCAAGAACTG |
| *Pten* | ENSMUSG00000013663 | F: TCTTCTCCCCATTCCGCTGCCT  R: AAGGCTGGATTGACGAGCTCGC |
| *Tacc3* | ENSMUSG00000037313 | F: AGCCGACAGAAGCTGAGCTTGTCA  R: GAACAGCTGGTGGCCAAAACAGAT |
| *Trp53* | ENSMUSG00000059552 | F: ATAGAGACGCTGAGTCCGGTTC  R: CCTAAGCCCAAGAGGAAACAGA |

### **Primers for RT-PCR**

| **Transcript** | **Transcript-ID** | **Primer Sequence**  **(5´… 3´)** |
| --- | --- | --- |
| *Ahcyl1* | ENSMUST00000029490 | F: CCAAGGAGATCGAGGACGCCGA  R: ACGAGACAAAGATCTCCGGCCA |
| *Fgfr2* | ENSMUST00000122054 | F: GGCATGCTGTACCCTCACAG  R: GTGAGATCCAAGTATTCCTCATTGG |
| *Fgfr2-Ahcyl1 Fusion* | - | F: GACAAGCCCACCAACTGCACCA  R: ACGAGACAAAGATCTCCGGCCA |
| *Gapdh* | ENSMUST00000117757 | F: CATCACTGCCACCCAGAAGACTG  R: ATGCCAGTGAGCTTCCCGTTCAG |
| *‍Cxcl1* | ENSMUSG00000029380 | F: TCCAGAGCTTGAAGGTGTTGCC  R: AACCAAGGGAGCTTCAGGGTCA |
| *Cxcl10* | ENSMUSG00000034855 | F: ATCATCCCTGCGAGCCTATCCT  R: GACCTTTTTTGGCTAAACGCTTTC |
| ‍*Cxcl2* | ENSMUST00000200681.3 | F: CATCCAGAGCTTGAGTGTGACG  R: GGCTTCAGGGTCAAGGCAAACT |
| *‍*Cxcl9 | ENSMUST00000113093.5 | F: CCTAGTGATAAGGAATGCACGATG  R: CTAGGCAGGTTTGATCTCCGTTC |
| *‍Dusp6* | ENSMUSG00000017724 | F: CTCGGATCACTGGAGCCAAAAC  R: TCTGCATGAGGTACGCCACTGT |
| *‍Etv4* | ENSMUSG00000017724 | F: CACAGACTTCGCCTACGACTCA  R: GCAGACATCATCTGGGAATGGTC |
| *‍Rps14* | ENSMUSG00000024608 | F: GACCAAGACCCCTGGACCT  R: CCCCTTTTCTTCGAGTGCTA |
| *‍TNF alpha* | ENSMUSG00000024401 | F: GGTGCCTATGTCTCAGCCTCTT  R: GCCATAGAACTGATGAGAGGGAG |
| *‍‍Prf1* | ENSMUST00000035419.7 | F: ACACAGTAGAGTGTCGCATGTAC  R: GTGGAGCTGTTAAAGTTGCGGG |

1.5 **Biological samples**

| **Description** | **Source** | **Identifier** |
| --- | --- | --- |
|  |  |  |

1.6 **Deposited data**

| **Name of repository** | **Identifier** | **Link** |
| --- | --- | --- |
| RNA seq | PRJEB113177 | <https://www.ebi.ac.uk/ena/browser/view/PRJEB113177> |
| ‍MIBI | S-BIAD2557 | <https://www.ebi.ac.uk/biostudies/bioimages/studies/S-BIAD2557> |

1.7 **Software**

| **Software name** | **Manufacturer** | **Version** |
| --- | --- | --- |
| Affinity Designer | Serif | 2.x |
| CFX Maestro | BIORAD | 4.1.2.433.1219 |
| ‍Design and Analysis | Thermo Fisher Scientic | 2.8.0 |
| GE ‍Amersham Imager 680 | GE Healthcare Life Sciences | V2.0.0 |
| ‍GloMax Explorer | Promaga | V3.2.3 |
| GraphPad Prism | GraphPad | 11 |
| ‍‍Living Image | PerkinElmer Health Sciences | 4.5.5 |
| QuPath | QuPath | 0.7.0-x64 |
| ‍Python |  | 3.10.19, 3.10.20, and 3.11.5 |
| ‍‍Toffy |  | 0.1.1 |
| ‍‍Ark-analysis |  | 0.7.0 |
| ‍‍Cellpose |  | 2.2.1 |
| ‍‍FlowSOM |  | 0.1.15 |
| ‍‍UELer |  | 0.3.0a0 |
| ‍‍SCIMAP |  | 2.2.11 |
| ‍‍Scanpy |  | 1.11.5 |
| ‍‍pymer4 |  | 0.9.2 |
| ‍R | R Foundation for Statistical Computing | 4.4.3 |
| DESeq2 |  | 1.46.0 |
| ‍fgsea |  | 1.32.4 |
| ‍clusterProfiler |  | 4.14.6 |
| GOSemSim |  | 2.32.0 |
| ‍ggplot2 |  | 4.0.3 |
| ‍dplyr |  | 1.2.1 |
| ‍pheatmap |  | 1.0.13 |
| ‍RColorBrewer |  | 1.1-3 |
| ‍ggrepel |  | 0.9.8 |
| ‍stringr |  | 1.6.0 |
| ‍org.Mm.eg.db |  | 3.20.0 |
| ‍AnnotationDbi |  | 1.68.0 |
| ‍BiocParallel |  | 1.40.2 |

1.8 **Other (*e.g*. drugs, proteins, vectors etc.)**

| *pSpCas9(BB)-2A-GFP (PX458) V2.0* | Addgene | # 48138 |
| --- | --- | --- |
| *pSpCas9(BB)-2A-Puro (PX459) V2.0* | Addgene | # 62988 |
| *AAV-KPL* | Addgene | # 60224 |
| Pemigatinib | MedChemExpress | #HY-109099 |
| Futibatinib | MedChemExpress | #HY-100818 |

1.9 **Please provide the details of the corresponding methods author for the manuscript:**

Michael T. Dill, MD PhD

German Cancer Research Center (DKFZ)

Im Neuenheimer Feld 280, 69120 Heidelberg, Germany

Tel.: +49 6221 56 34064

E-Mail: michael.dill@med.uni-heidelberg.de

ORCID: 0000-0002-1926-4204

**2.0	Please confirm for randomised controlled trials all versions of the clinical protocol are included in the submission. These will be published online as supplementary information.**

N/A
